# Development of Spheroid-FPOP: An In-Cell Protein Footprinting Method for 3D Tumor Spheroids

**DOI:** 10.1021/jasms.2c00307

**Published:** 2023-01-26

**Authors:** Raquel L. Shortt, Yijia Wang, Amanda B. Hummon, Lisa M. Jones

**Affiliations:** † Department of Pharmaceutical Sciences, University of Maryland, Baltimore, Maryland 21201, United States; ‡ Department of Chemistry and Biochemistry, The Ohio State University, Columbus, Ohio 43210, United States; § Department of Chemistry and Biochemistry, University of California San Diego, La Jolla, California 92093, United States

## Abstract

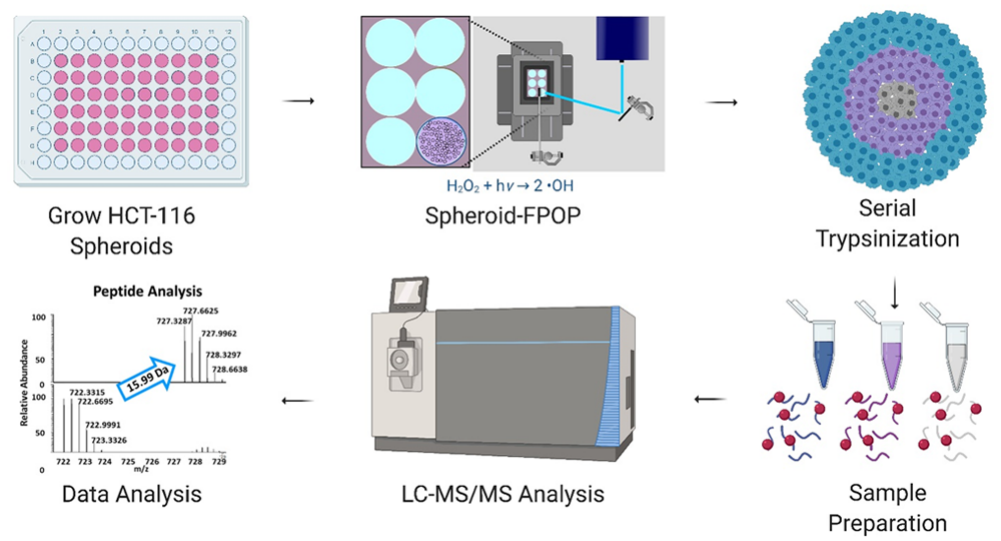

Many cancer drugs fail at treating solid epithelial tumors with hypoxia and insufficient drug penetration thought to be contributing factors to the observed chemoresistance. Owing to this, it is imperative to evaluate potential cancer drugs in conditions as close to *in vivo* as possible, which is not always done. To address this, we developed a mass spectrometry-based protein footprinting method for exploring the impact of hypoxia on protein in 3D colorectal cancer cells. Our group has previously extended the protein footprinting method fast photochemical oxidation of proteins (FPOP) for live cell analysis (IC-FPOP); however, this is the first application of IC-FPOP in a 3D cancer model. In this study, we perform IC-FPOP on intact spheroids (Spheroid-FPOP) using a modified version of the static platform incubator with an XY movable stage (PIXY) FPOP platform. We detected modification in each of three spheroid layers, even the hypoxic core. Pathway analysis revealed protein modifications in over 10 distinct protein pathways, including some involved in protein ubiquitination; a process modulated in cancer pathologies. These results demonstrate the feasibility of Spheroid-FPOP to be utilized as a tool to interrogate protein interactions within a native tumor microenvironment.

## Introduction

Cancer remains one of the leading causes of death worldwide, emphasizing the need for scientists to invest in understanding its biological mechanisms. Traditionally, two-dimensional (2D) cell culture methods have been used to model the disease; however, they are limited in that they do not reflect the human tumor microenvironment. In 2D cultures, cells grow in a monolayer which alters the cellular cytoskeleton, limits exposure to the extracellular matrix, disables cell–cell/cell–matrix interactions, and results in the loss of tissue specific architecture.^1^ For these reasons, researchers have moved to three-dimensional (3D) cell culture systems, where cells are maintained in an environment that enables the formation of cellular aggregates known to mimic the tumor microenvironment (TME).^2^

A 3D model capable of studying cancer disease states is multicellular tumor spheroids. Spheroids exhibit a complex architectural structure, hypoxia toward the core, an extracellular matrix, nutrient and pH gradients, and dynamic cell–cell/cell-matrix interactions, similar to that of human tumors.^1,3^ An advantage of employing 3D cancer models is their well-defined geometry, enabling scientists to relate structure to function and gene expression. Furthermore, spheroids can be easily propagated with high reproducibility, making them an attractive system for studying cancer pathogenesis and developing therapeutics. The spheroid model system has been widely used to study regulation of proliferation, metabolism, differentiation, cell death, invasion, angiogenesis, and immune response.^3^ More recently, scientists have employed the spheroid model to study the cellular interactions that contribute to therapeutic resistance in cancer.^3^

Chemoresistance is a prominent issue in cancer drug failure. There are multiple means for target resistance against cancer therapeutics including pharmacokinetic-based resistance that includes efflux by ABC transporters and drug metabolizing enzymes.^4^ Studies have determined that some resistance can arise from the effects of hypoxic conditions in the interior of tumors and poor drug penetration, issues that cannot be studied in monolayer cell cultures. In contrast, spheroids display an oxygen gradient similar to tumors making them a suitable model for studying the effect of hypoxia on therapeutic efficacy. Hypoxia in tumors has been shown to alter cellular functions, enzymatic reactions, protein conformation, and drug metabolism, which can make it difficult to identify therapeutic targets and develop reliable treatments.^5,6^ The ability to study spheroids either as a whole or by layer provides an excellent system to tease out the effect of these altered mechanisms.^7,8^ These aspects are critical for the development of reliable biopharmaceuticals. Spheroid research can interrogate the relationship between hypoxia in tumors and protein conformations within the radial heterogeneous population of cells within the mass. However, this requires innovative methods to examine protein heterogeneity of the tumor spheroid proteome.

Mass spectrometry (MS)-based protein footprinting methods, such as hydroxyl radical protein footprinting (HRPF) and hydrogen–deuterium exchange (HDX), have been used to interrogate the higher order structure (HOS) of proteins and associated dynamics.^9^ HRPF probes protein structure by imparting an irreversible covalent label on solvent accessible amino acid side chains. The resultant labeling pattern is a footprint of the structure and interactions of the protein at the time of the labeling event. Differential experiments comparing the labeling modifications are used to evaluate the effect of an experimental condition (i.e., ligand bound versus ligand free or drug treatment versus no drug treatment). Therefore, experiments can be designed to investigate specific biological questions. Detection of the modifications are typically performed with bottom-up proteomics which allows for quantitation on the extent of modification at the peptide or residue-level.^10^ The HRPF method fast photochemical oxidation of proteins (FPOP) uses an excimer laser (248 nm) to generate hydroxyl radicals via hydrogen peroxide (H_2_O_2_) photolysis. The most prevalent modification result is a +16 Da mass shift, although other modifications can occur.^11^ The FPOP platform labels proteins on the microsecond time scale, making it a useful structural biology tool for numerous biological applications. For instance, FPOP has been used to study membrane proteins,^12^ map epitopes, and probe protein folding.^13−16^ Recently, FPOP has been extended in cells (IC-FPOP) and its efficacy has been validated across many cell lines, and has been applied in *C. elegans* in a technique entitled *in vivo*-FPOP.^17−19^ IC-FPOP is a valuable structural biology tool as information gathered from its studies reflects that of native environments. Although FPOP has had a myriad of applications, its extension in 3D cancer models is advantageous for progressing therapeutic development.

Another MS-based technique that has been used to probe spheroids is mass spectrometry imaging (MSI). MSI is a label-free method used to localize molecules in various biological systems.^20^ By taking advantage of multichannel MS measurements pixel by pixel on a sample surface, MSI allows the analysis of biological molecules simultaneously on the surface of biological samples. Each independent measurement (*m*/*z*) evaluates the spatial distribution of a molecular species in the sample, which facilitates the generation of images respectively for dozens to hundreds of compounds.^21^ Among the MSI methods, matrix-assisted laser desorption/ionization (MALDI) MSI has remained a popular ionization method since its first application to localize proteins and peptides in different rat tissues by Caprioli and coauthors in 1997.^22^ This technique is widely used due to its relatively low sample requirements, high sensitivity and large mass range. Numerous molecular species can be detected at the same time, including metabolites,^23^ lipids,^24^ and peptides.^25^ Since that first study, the types of biological systems imaged have been expanded from biological tissues to smaller samples, such as 3D cell cultures. In 2011, Li and Hummon first used MALDI-MSI to map several proteins in HCT 116 spheroids.^26^ The spatial distribution provided by MALDI-MSI is critical to study the heterogeneity of spheroid TME.

**Figure 1:**
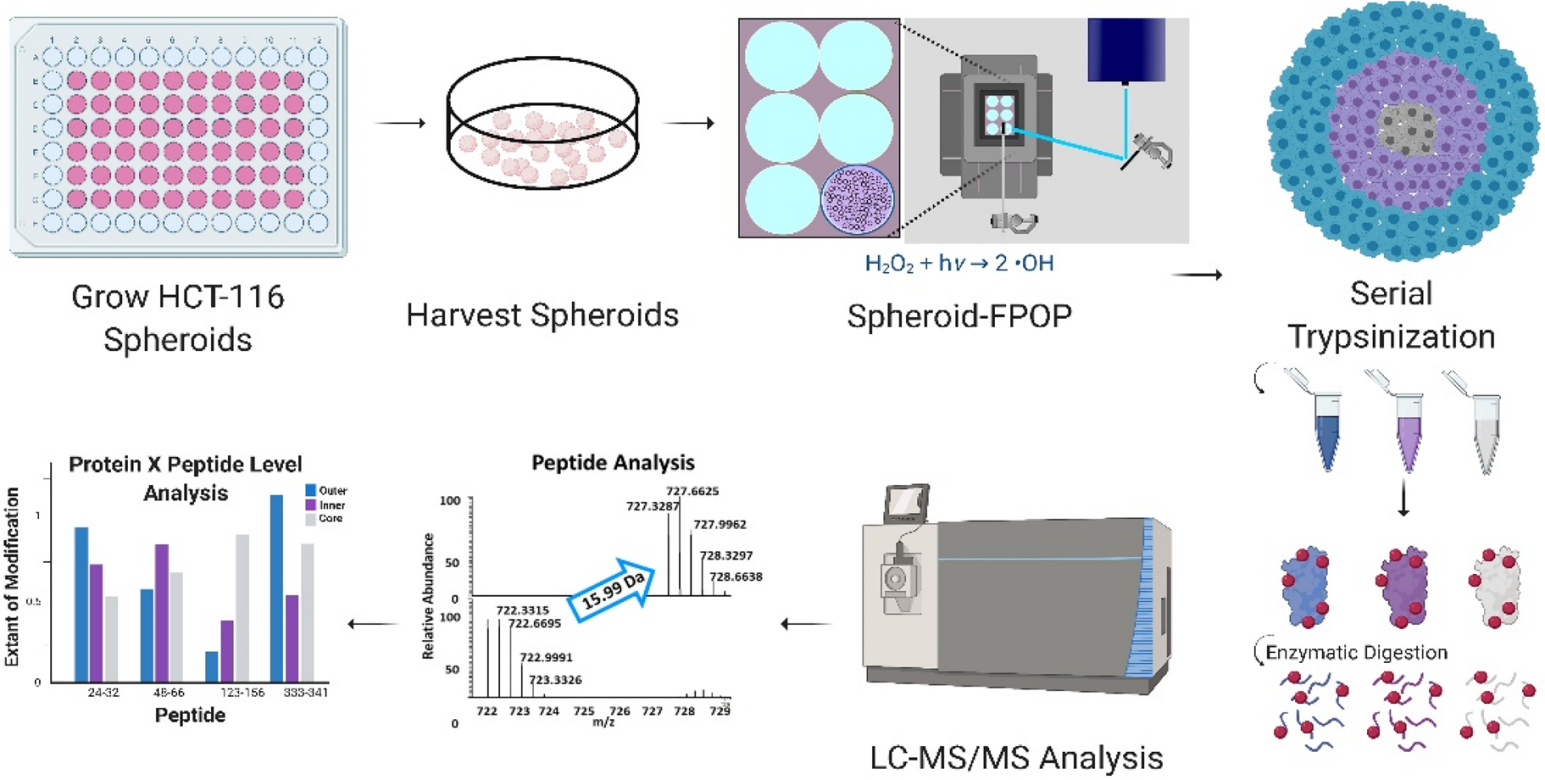
Spheroid FPOP workflow. Each step in the Spheroid-FPOP workflow is visualized where spheroids are first grown, harvested, and subjected to FPOP. Following hydroxyl radical labeling and reaction quenching, the spheroids were serial trypsinized. The cell lysates were then prepared for bottom-up proteomics analysis.

In this report, we describe the development of an in-cell footprinting workflow amenable with 3D tumor spheroids to examine protein interactions within a native TME (Figure 1). To confirm peroxide diffusion throughout the spheroid, MSI experiments were performed to detect changes in lipid chemistry upon hydrogen peroxide incubation with the spheroids. Minor augmentations were made to the IC-FPOP platform in developing this system. These include alterations to the static platform incubator with an XY movable stage (PIXY) system,^27^ since the traditional microflow system was not compatible with spheroids. Post-FPOP labeling, serial trypsinization was used to separate three individual layers of the spheroids. Serial trypsinization is a method used to sequentially peel cells from the outermost radial layers in spheroids, analogous to “peeling an onion”.^28^ Previously, this method was used in the Hummon lab to spatially quantify the chemotherapeutic and its metabolites in spheroids^29,30^ and to study the proteomic changes and identify protein biomarkers in different layers of SILAC spheroids.^31,32^ We demonstrate the feasibility of Spheroid-FPOP for protein structural analysis within the context of a native TME. We explore the distribution of FPOP modifications throughout three distinct spheroid layers, even the hypoxic core, and analyze global and residue-level extent and occurrence of modification. This novel technique enables the study of the relationship between protein structure and function in the context of a cancer model system. Protein modification via IC-FPOP on spheroids is a practical solution to explore structural heterogeneity of proteins in 3D models, which is likely a contributor to therapeutic inefficiency. This is due to the method’s versatile applications and the nature of the hydroxyl radical label. IC-FPOP has been evaluated on several cell lines and in *C. elegans*.^18,19^ So, expanding the applications of the novel method into 3D model systems advances the field of structural proteomics and illuminates its potential to progress biotheraputic development. Additionally, FPOP modifications are nonspecific and irreversible, making the technique an ideal candidate to uncover native structural information on the proteins in a variety of biological systems, as time nor residue is a limitation. When this technology is coupled with techniques that increase spatial resolution; like serial trypsinization, the structural data obtained is correlated with its radial location within the tumor model. In this way, the data can be used to relate the protein structure to its function. Understanding this relationship can unlock biological innovations and solutions. In all, the advantage of in vivo FPOP over other proteomics techniques is its ability to irreversibly label proteins in their native cellular within complex model systems, without time or specificity constraints. Spheroid-FPOP has the capacity to expand on what is currently known about the role of hypoxia on proteins within tumors.

## Experimental Section

### Chemicals and Reagents

Agarose, diluted trypsin (0.25%), RIPA buffer, *N*-*tert*-butyl-α-phenylnitrone (PBN), *N*,*N*′-dimethylthiourea (DMTU), and 1-palmitoyl-*sn*-glycero-3-phosphocholine (LysoPC(16:0)) were purchased from Millipore Sigma (St. Louis MO). Hydrogen peroxide, Pierce’s gold standard BCA assay, trypsin, tris base, hydrochloric acid, acetone, formic acid, Pierce colorometric peptide quantification assay, MS grade 0.1% formic acid in water, MS grade 0.1% formic acid in acetonitrile, and MS grade water were purchased from Thermo Fisher Scientific (Waltham, MA). McCoy’s 5A, fetal bovine serum (FBS), l-glutamine, phosphate-buffered saline (PBS), and 0.05% porcine trypsin with 0.1% EDTA were purchased from Gibco (Gaithersburg, MD). Iodoacetamide (IAA) was acquired from ACROS Organics. Dithiothreitol (DTT) was purchased from American Bio (Canton, MA). Dimethyl sulfoxide (DMSO) was purchased from Invitrogen (Carlsbad, CA).

### Two-Dimensional Cell Culture

HCT 116 colorectal carcinoma cells were purchased from the American Type Culture Collection (ATCC, Manassas VA). Cells were grown in a 175 cm^2^ flask and passaged at 90% confluency. The cell culture media consisted of McCoy’s 5A media supplemented with 10% fetal bovine serum (FBS) and 5% l-glutamine. Exogenous growth conditions were 37 °C with 5% CO_2_. Cells between passages 3–20 were utilized to initiate spheroid cell culture.

### 3D Cell Culture Plate Preparation

Spheroids were grown as previously described by Friedrich *et al.*(33) using 96-well flat-bottomed microplates (Thermo Fisher Scientific, Rochester NY) filled with an agar meniscus. Agarose weighing 0.15 mg was added to 10 mL of McCoy’s 5A media and heated to 180 °C for 20 min under high pressure (0.6 bar). Agar was kept in a water bath at ∼90 °C to prevent it from solidifying (Tayama Appliance, CA). To circumvent the outermost wells from drying out, a phenomenon known as “the edge effect”, only the inner 60 wells were used to grow spheroids while the remaining outer 36 wells were filled with PBS. After agar-media sterilization, 65 μL of agar was plated in the inner 60 wells of the 96-well plate angling the pipet tips to the plate so as the agar solidified it created a meniscus. The plates were kept at room temperature until the agar solidified. Unused agar plates were stored in a 4 °C refrigerator for up to 1 week.

### HCT 116 3D Cell Growth Formation

Trypsin-EDTA (25%) was used to enzymatically dissociate confluent (∼2.8 × 10^6^ cells) HCT 116 cells from a T-25 cm^2^ culture flask (Corning, Corning NY). All cells were collected and washed via centrifugation at 12,000*g* for 5 min with PBS × 3. The cell pellet was dissolved in 10 mL of complete media, and the cell suspension was combined with 140 mL of complete media for a total of 150 mL (∼18600 cells/mL). A volume of 200 μL of cell suspension was added to each of the inner 60 wells of 12 96-well plates. Plates were stored in a cell culture incubator with 5% CO_2_ for 14 days with media change occurring on the tenth and twelfth day.

### MALDI-MSI

Spheroids treated with different FPOP conditions were embedded in gelatin and cryosectioned to 12 μm thick and thaw-mounted to indium tin oxide (ITO) slides. Matrix solution was prepared by dissolving 2,5-dihydroxybenzoic acid (DHB) (Sigma-Aldrich, St. Louis, MO) in 50:50 acetonitrile–water with 0.1% formic acid (FA) at a concentration of 10 mg/mL. Matrix solution was sprayed onto the slides using a TM sprayer. A 15T Solarix FT-ICR (Bruker Daltonics, Billerica, MA) was used to detect molecules at an *m*/*z* range of 92 to 1000 in positiveion mode. The laser spot size was set to small with the raster of 50 μm along both the x and y axes. The images and intensity plots were processed using SCiLS Lab 2021c (Bruker Daltonics, Billerica, MA). All spectra were normalized against total ion count, using peak intensity divided by the sum of all signal intensities in the mass spectra.

### Spheroid FPOP

Spheroid FPOP was done in technical triplicates and two biological replicates. The laser energy required for the FPOP experiment was produced using a GAM 248 nm KrF excimer laser. The optical lenses of the laser were oriented to ensure 100% coverage of the 35 × 10 mm surface area. Laser frequency and energy were adjusted to ensure a laser intensity of at least ∼120 mJ which was verified using an Ophir laser sensor (Ophir Spiricon, North Logan, Utah). To quench the FPOP reaction, a solution of PBN and DMTU at a concentration of 125 mM and 1% DMSO was prepared.

Twelve plates of spheroids were grown for each condition, namely 100 and 200 mM H_2_O_2_. Spheroids were harvested from 12 96-well plates by placing ∼120 spheroids into a 35 × 10 mm low attachment dish (Greiner Bio-One, Monroe North Carolina) containing 1 mL of McCoy’s 5A media. Before FPOP labeling, McCoy’s 5A media was removed and the spheroids were rinsed briefly with PBS. After rinsing the spheroids and discarding the PBS, 1 mL of PBS was added to the 35 × 10 mm disc followed by 1 mL of either 200 or 400 mM H_2_O_2_ for a final concentration of 100 or 200 mM H_2_O_2_, respectively. The spheroids were incubated with hydrogen peroxide for 1 min and exposed to one laser pulse of 50 Hz at 27 kV. 2 mL of the quench solution was immediately added after labeling. Controls included a group of spheroids that were grown, harvested, and prepared in that same fashion as the sample/FPOP group, except they were not exposed to laser irradiation..

### Serial Trypsinization

After spheroid-FPOP, samples and control spheroids were serial trypsinized.^6^ The quench solution was removed from the Petri dish containing the spheroids. Chilled 0.05% trypsin was placed in the dish and oscillated for 3 min to allow the manual dissociation of the outermost layer of the spheroids. To halt enzymatic proteolysis, chilled complete media was added to the well and again oscillated for 3 min. The cell suspension was collected on ice and chilled. McCoy’s 5A was introduced to the spheroids to remove any traces of FBS and oscillated for 3 min. The cells were gathered with the first aliquot, and the three-step procedure was completed two more times to acquire the outer layer of the spheroids. The inner layer was obtained via the same three-step process carried out in triplicate. The result is a cell suspension of outer and inner layers. The cores were briefly rinsed with PBS and collected in microcentrifuge tubes.

### Protein Extraction and Digestion

All spheroid layers were washed with PBS three times by agitating the cell suspension in PBS then spinning at 16,000*g* for 10 min removing the supernatant and repeating. RIPA buffer was used to lyse the cells followed by protein quantification via Pierce’s gold standard BCA assay. Samples containing 100 μg/mL of protein were then reduced with DTT and alkylated with IAA. Following alkylation, an overnight acetone precipitation was performed, and the precipitate was dissolved in 10 mM Tris pH 8. Samples were then enzymatically digested overnight with trypsin at a 1:20 protein to enzyme ratio. Trypsin was quenched using formic acid at 5% of the total sample volume. Samples were concentrated using a speed-vac and dissolved in 10 mM Tris buffer prior to peptide quantification via colorimetric assay. Sample containing 10 μg of peptide was aliquoted into separate tubes and concentrated to remove Tris buffer. After sample concentration, 10 μg of peptide was dissolved in 0.1% FA in water and transferred in fresh autosampler vials.

### Liquid Chromatography–Tandem Mass Spectrometry (LC–MS/MS)

Peptides were loaded unto an Acquity C18 nanotrap column with a Waters Acquity M-class UPLC. Peptide separation was achieved through a custom-packed silica capillary column filled with RP C18 material. The gradient flow rate was set at 0.3 μL/min from 10 to 45% solvent B (0.1% FA in acetonitrile) for 100 min, 100% Solvent B for 5 min, and re-equilibrated to 97% Solvent A 3% Solvent B. The total gradient run time was 130 min. Mass spectrometric analysis was performed on an Orbitrap Fusion Lumos in data dependent acquisition mode using Xcalibur software (Thermo-Fisher Scientific, Waltham, MA). Mass spectra of the peptides were analyzed over a 375–1500 *m*/*z* range at normal mass resolving power with a 60 s dynamic exclusion (Orbitrap Resolution 60000). The most abundant multiply charged ions were fragmented by higher-energy collisional dissociation (HCD). Singly charged ions were rejected, and monoisotopic ion selection was applied. The AGC target was 5.5e5 with a maximum injection time of 50 ms with a 5.0e4 intensity threshold. Ions having charge states of 2–6 were excluded. MS2 ions were exposed to high-energy collisional dissociation (HCD) (32% normalized collision energy) and detected with 15000 resolution in the orbitrap and a 5.0e4 AGC target.

### Data Analysis

Proteome Discoverer 2.2 (Thermo Fisher Scientific, Waltham, MA) analyzed all MS/MS samples using the Sequest algorithm. The files were searched against a SwissProt *Homo sapiens* database. A unique multilevel workflow was used for database searching.^34^ This workflow contains five search algorithm levels where the commonly observed FPOP modifications were dispersed across the individual search levels. The tolerance for fragment ions was set at 0.02 Da and the precursor ion tolerance at 10 ppm. The enzyme specificity was set to trypsin and considered only one missed cleavage. Peptides were dissociated and filtered for the false discovery rate (FDR) of 5%. Proteins were accepted if at least two distinct peptides were identified with the 5% FDR filter. The resultant consensus files were exported and analyzed by Microsoft Excel and PowerPivot add-in. The fractional oxidation per peptide or residue was calculated according to the following equation: (∑ EIC area modified)/ (∑ EIC area). For residue-level analysis, the EIC area modified represents EIC area of a PSM for a particular modified residue. Meanwhile, EIC area is the EIC area of a PSM with sequences identical to those with the modification. From this, quantitation of the variances in protein expression, proteome coverage and extent of modification in individual layers of spheroids were determined.

## Results and Discussion

### MALDI-MSI to Determine H_2_O_2_ Penetration

**Figure 2:**
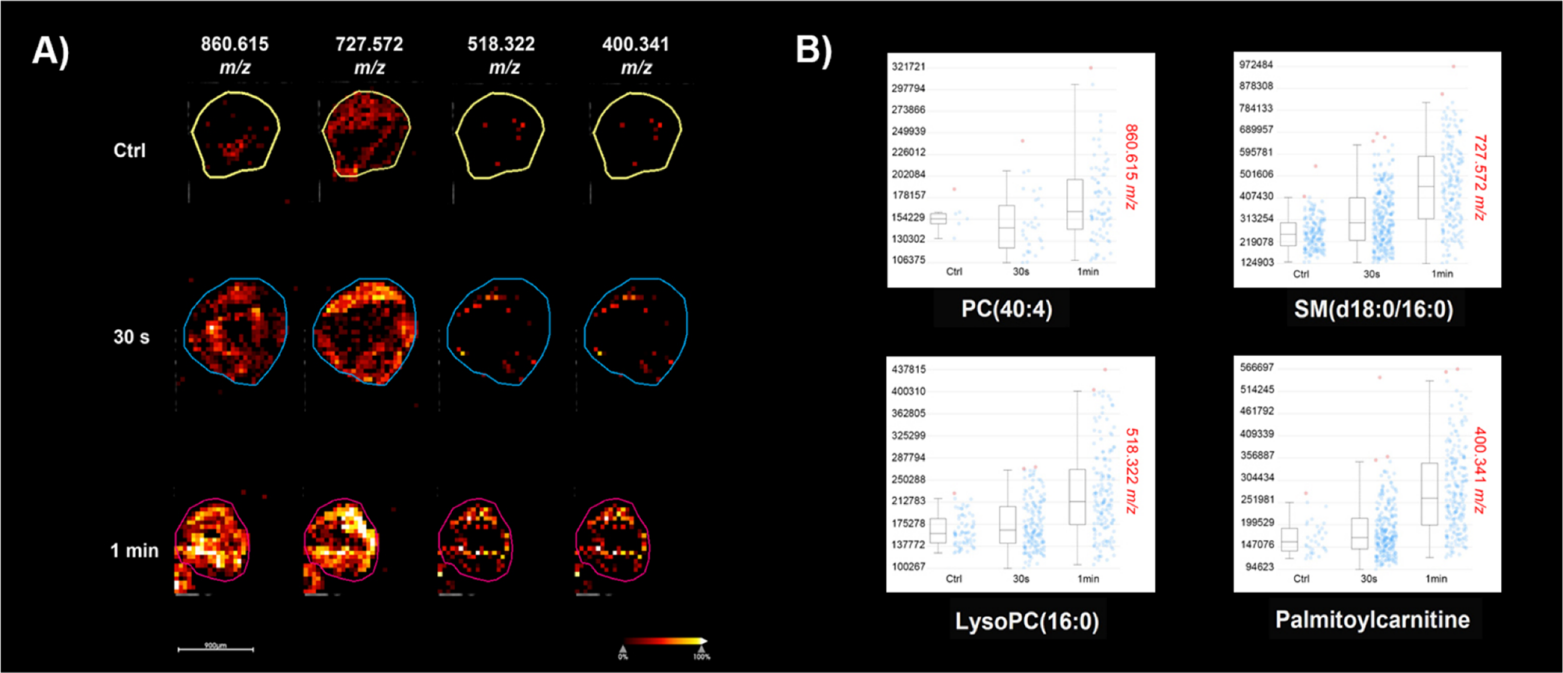
MALDI MSI showing time-dependent lipid penetration in hydrogen peroxide treated spheroids as a surrogate for hydrogen peroxide penetration. (A) MSI data acquired on a 15 T FTICR showing time-dependent penetration of four representative lipids within 1 min treatment. (B) Intensity plots of the images showing increasing signal intensity with treatment time and the putative identifications based on database search through METASPACE. For all four lipids, the signal intensity increased with hydrogen peroxide treatment.

The effectiveness of IC-FPOP to study spheroids is dependent on the ability to modify proteins in all areas of the spheroids including the core. This requires diffusion of H_2_O_2_ throughout the spheroid in a short time span. To assess the penetration of small molecules, we developed an MSI method that measures changes in lipid chemistry that occur from exposure to the hydrogen peroxide. Hydrogen peroxide is too small to be easily detected by MALDI because of matrix interference at low mass range. However, hydrogen peroxide is a strong oxidant and can cause both oxidation^35^ and peroxidation^36^ of lipids in cells. Although the products from the oxidations are complex, making it difficult to detect the modified lipids directly, the secondary effect of this process can cause lipid penetration and these molecules could be used as a surrogate for direct measurement of hydrogen peroxide. HCT 116 spheroids were exposed to 100 mM H_2_O_2_ for different time points within 10 min. Spheroids treated for greater than 5 min lost structural integrity and could not be sectioned for MSI analysis. For this reason, we used shorter treatment times of 30 and 60 s. The corresponding MALDI-MSI data show that incubation with hydrogen peroxide caused alterations in lipid composition throughout the spheroids within 1 min (Figure 2A). These representative *m*/*z* values were selected based on several steps. A discriminative analysis was first performed in SciLS Lab between the control and 1 min treated spheroids, and the imaging data of a representative 1 min sample were loaded to the METASPACE platform (https://metaspace2020.eu) to search in four databases, HMDB v4, LipidMaps 2017-12-12, CoreMetabolome v3, and PAMDB v1.0, for more molecule information. Those *m*/*z* values in the discriminative spectrum were selected as the markers to show the penetration of hydrogen peroxide. From this correlation, these four *m*/*z* were identified as PC(40:4), SM(d18:0/16:0), LysoPC(16:0), and palmitoylcarnitine (Figure 2B). Further identification of one of these, LysoPC(16:0), was confirmed by tandem MS and use of a standard (Figure S1). MS/MS analysis of a 1 min treated sample was compared with a standard lipid solution of LysoPC(16:0), with the precursor peak (518.3213 *m*/*z* in the spheroid sample and 518.3211 *m*/*z* in the standard solution, Δppm 0.4) and two fragments (459.2480 *m*/*z* in the spheroid sample and 459.2474 *m*/*z* in the standard solution, Δppm 1.3; 307.7591 *m*/*z* in the spheroid sample and 307.7571 *m*/*z* in the standard solution, Δppm 6.5) matching well with each other (Table S1). Confirmation of additional identified lipids is ongoing but the observed lipid alterations demonstrate complete H_2_O_2_ diffusion throughout the spheroid within 1 min of incubation time.

### Optimization of the Spheroid-FPOP Platform

Given the observed pervasiveness of H_2_O_2_ in the MSI experiments, an incubation time of 1 min with 100 mM H_2_O_2_ was used for subsequent FPOP studies. Spheroid-FPOP experiments were attempted using the IC-FPOP microflow system where previous work demonstrated the capability to footprint various cell lines.^17,37^ The flow system uses a network of silica tubing for hydrodynamic focusing to achieve single-cell flow. A similar approach was taken for Spheroid-FPOP but, to accommodate the larger size of the spheroids, a flow tube with an internal diameter of 1815 μm was used (Figure S2). During the flow system studies, the required flow rate caused undesirable sheering which resulted in disintegration of the spheroids inside of the capillary. To circumvent the sheer forces of the flow system, we employed the use of PIXY, a static FPOP platform. IC-FPOP via the PIXY platform successfully modified more proteins than the traditional microflow system, in a fraction of the time, in both monolayer cells and *C. elegans*.^27^ In the PIXY platform, two 50 mm optical mirrors are aligned to project the laser beam down onto a stage top incubator. In the initial study of IC- and IV-FPOP on PIXY, a set of peristaltic pumps controlled the flow of FPOP reagents followed by manual firing of the excimer laser to initiate FPOP labeling. For Spheroid-FPOP using PIXY, neither the incubator nor the peristaltic pumps were necessary. All reagents were manually added to preserve spheroid structure. Although the use of PIXY increased the spheroid recovery rate for FPOP labeling, spheroid adherence to the plastic culture dish once they were harvested from culture media was observed. This issue was even more prominent post-FPOP. To mitigate the observed adhesion, spheroids were harvested in a low bind 35 mm Petri dish and remained in the same dish until MS sample preparation. We also maintained the spheroids in complete media until immediately before FPOP labeling to prevent premature aggregation or adhesion. To perform FPOP, the Petri dish was placed directly on top of the PIXY platform. Reagent addition, firing of the laser pulse, and reagent removal was done manually, foregoing the use of the automated platform movement and peristaltic pumps. This modification to FPOP on the PIXY platform, ensured high recovery rate of intact spheroids during experimentation. The post labeling procedures commenced without delay. Post-FPOP labeling, we utilized a serial trypsinization method to separate out the spheroid layers.^7,33^ This provided spatial resolution so we could determine whether proteins in the different spheroid layers were modified. It is completed by incubating spheroids in dilute trypsin for a short time. When the trypsin reaction is quenched by the addition of media with FBS, the cell suspensions are collected, and the remaining spheroids are washed for further trypsin proteolysis. Serial trypsinization was used to separate three distinct layers, an outer, inner, and core layer. Lysates from each layer were analyzed separately by MS analysis.

### Protein Modification by Spheroid-FPOP

**Figure 3:**
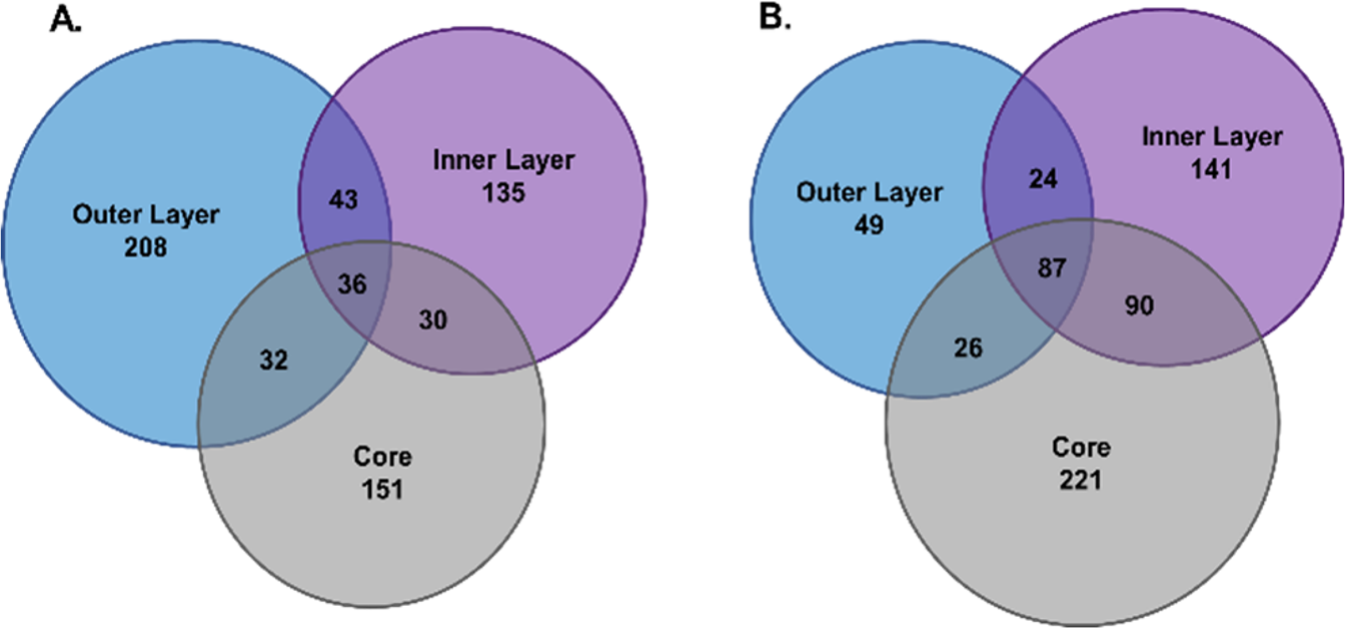
(A) Total number of modified proteins for 100 mM H_2_O_2_ 1 min exposure: 635. Outer layer: 319, inner layer: 244, and core: 249. 2B. Total number of modified proteins for 200 mM H_2_O_2_ 1 min exposure: 638. Outer layer: 186, inner layer: 342, and core: 424.

Global level oxidation of spheroids subjected to FPOP using PIXY revealed that 635 proteins were modified in total throughout the spheroid. Analysis of modifications in the three layers shows that 319 of the modified proteins occurred in the outer layer, 244 in the inner layer, and 249 in the core between two biological replicates (BR) (Figure 3A). The number of modified proteins was similar for each layer within the two BR, indicating that there was not significant sample variability for 100 mM H_2_O_2_ 1 min exposure (Figure S3). Modifications in the core indicate that H_2_O_2_ is reaching the hypoxic regions of the spheroids, a result that was also validated by the hydrogen peroxide-induced changes in the lipids detected by MSI. Interestingly, the number of modified proteins was not markedly lower in the core compared to the outer layer of the spheroid. This not only confirms the penetration of H_2_O_2_ into the spheroid core within the 1 min incubation time but also demonstrates penetration of the laser into the core. FPOP utilizes a laser wavelength, 248 nm, in the ultraviolet (UV) region for H_2_O_2_ photolysis. Wavelengths in this region tend to have shorter penetration depths in biological tissues than longer wavelength light. Optical absorption depth of collagen-based soft tissues varies from ≤0.5 μm at λ = 190 nm to ∼200–400 μm at λ = 400 nm.^38^ The diameters of spheroids used in this study are on average ∼1000 μm so a penetration depth of at least 500 μm is required to label proteins in the core. The spheroids in this study were seeded in an agar-based meniscus without the addition of collagen. This results in a lower collagen content which may increase the depth of laser penetration. We increased the H_2_O_2_ concentration to 200 mM for spheroid-FPOP to observe what changes this made to protein modification. Under these conditions, 638 proteins were modified (Figure 3B) across two biological replicates which is not a significant increase from the 100 mM condition. There were 186 proteins modified in the outermost layer, 342 proteins modified in the inner layer, and 424 in the core. The number of proteins modified per layer were not similar across BRs as one BR displayed many more modifications in each layer (Figure S4). This variability could be due to varying dissociation rates of the spheroids during serial trypsinization since the use of 200 mM H_2_O_2_ caused the spheroid layers to shed slightly faster than those exposed to 100 mM H_2_O_2_. This introduced inconsistency in that portion of our experimental workflow indicating 200 mM H_2_O_2_ is not an optimal concentration for spheroid FPOP studies.

**Figure 4:**
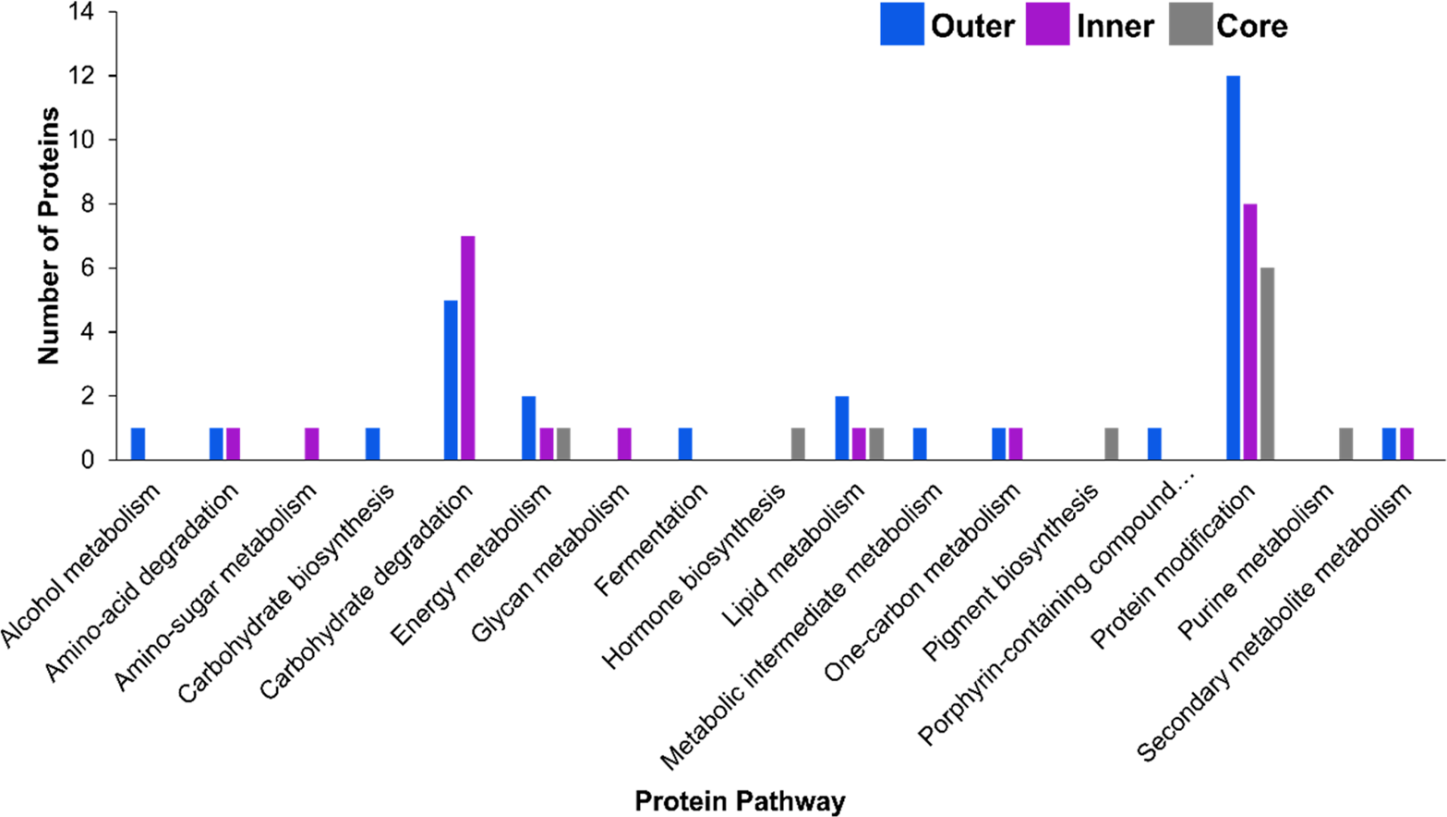
Number of proteins modified in each layer associated with a pathway by Uniprot The list of unique proteins that were modified by FPOP were submitted through the BLAST searching method available at Unirprot.org/blast. The resultant list of proteins and their associated pathways were counted and analyzed.

We calculated the global-level extent of oxidation on the 37 proteins that were modified in all three layers to compare the extent of oxidation (Table S2, Figure S5). Interestingly, there is not a clear pattern in the extent of modification such as the highest level of oxidation always being on proteins in the outer layer. In some cases, the most abundant modifications were found in the core layer. These differences are presumably due to differences in interaction networks or conformations in the different layers. This indicates that hydrogen peroxide diffusion and laser penetration depth does not alter the ability of FPOP to modify proteins based on solvent accessibility. For spheroid-FPOP to be useful as a method for studying protein HOS in cancer models, the method should modify proteins in pathways related to the disease state. UniProt pathway analysis was performed on the proteins modified in the 100 mM H_2_O_2_ condition (Figure 4). The most abundant pathway represented by modified proteins was protein modification, specifically, proteins that play a role in ubiquitination and deglycosylation (Table S3). Spheroid-FPOP was successful in modifying several ubiquitin ligase enzymes including E3 ubiquitin-protein ligase RNF216 and E3 ubiquitin-protein ligase RNFT1. Ubiquitination regulates both tumor-suppressing and tumor promoting pathways and dysregulation of ubiquitination is a contributing factor in cancer development.^39^ Glycosylation is also altered in cancer cells leading to an increase in branched-glycan structures that interfere with cell adhesion and promotes tumor invasion.^40^ The second most abundant pathway represented was carbohydrate degradation including proteins in the glycolytic pathway such as fructose-bisphosphate aldolase C and pyruvate kinase (4). Cancer cells reprogram their metabolic pathways including the upregulation of glycolysis as part of the Warburg effect.^41^ Understanding the roles of ubiquitination, glycosylation, and metabolic reprogramming in cancer can lead to the development of new treatments. The ability of spheroid-FPOP to modify proteins involved with these processes means the method can be used to study cancer related biological events.

### Residue-Level Modifications in Proteins

**Figure 5:**
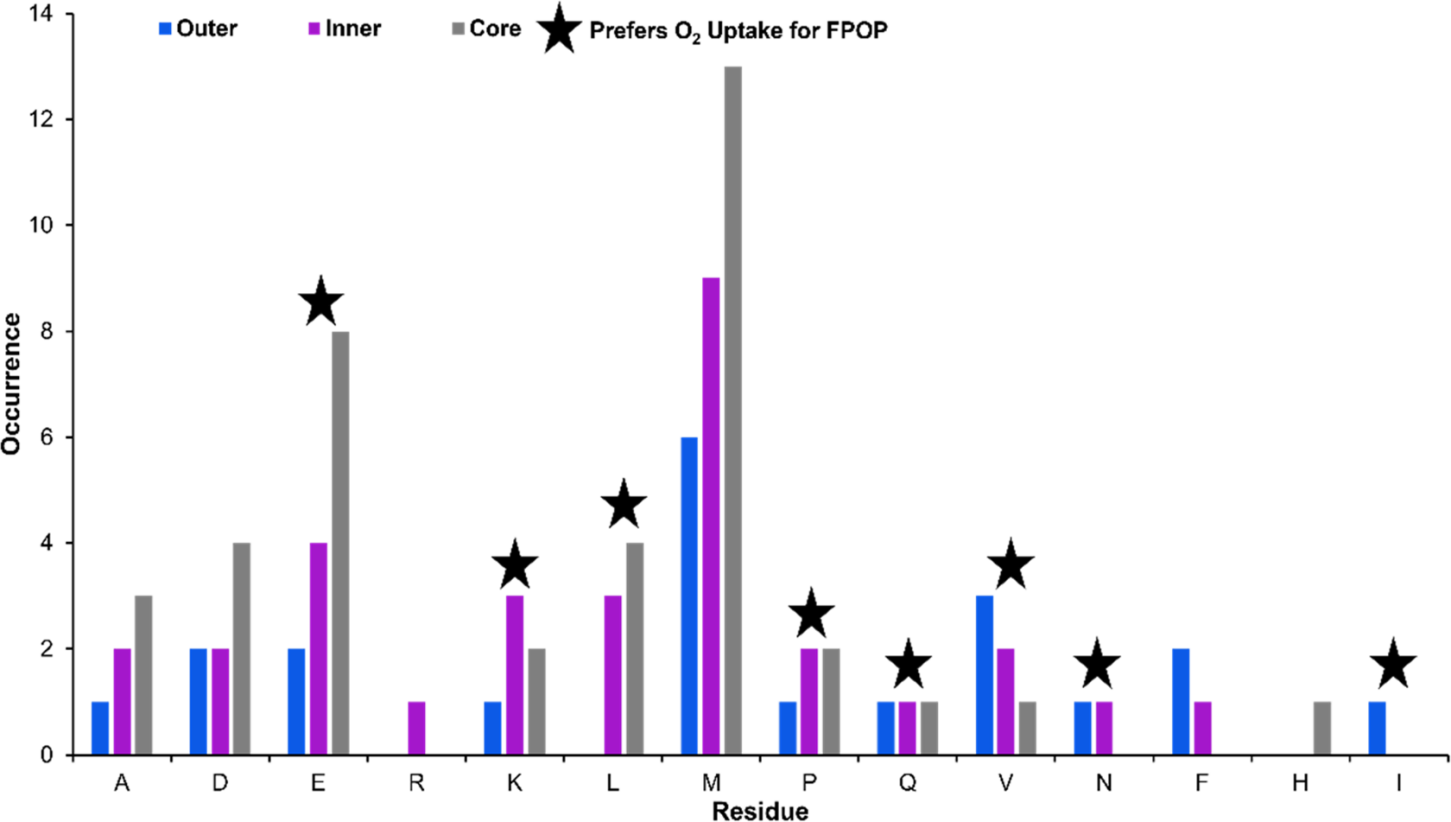
Residue modifications per layer. Residue level data analysis was performed and the number of times each residue was modified was graphed. Residues A, D, E, R, K, L, M, P, Q, V, N, F, H, and I were found to be modified.

To further interrogate spheroid-FPOP labeling, we probed the residue-level data to identify any patterns that would indicate how FPOP modifications are distributed among the three spheroid layers. This would deepen our understanding of the mechanisms of FPOP under hypoxic conditions, an important aspect considering many of the HRPF reactions require oxygen.^11^ A recent publication by Gross and co-workers described the use of isotopically labeled H_2_O_2_ and oxygen (O_2_) to determine hydroxyl radical labeling pathways on the FPOP platform.^42^ From this experiment, the researchers established oxidation pathways for 13 residues and revealed three distinct classes of amino acids based on their oxygen uptake preference. Class 1 residues preferentially uptake oxygen from H_2_O_2_, most likely via addition of hydroxyl radicals, class 2 residues from both H_2_O_2_ and dissolved O_2_, and class 3 residues from dissolved O_2_. Considering the oxygen gradient in spheroids, it would be interesting to correlate the residues modified by spheroid-FPOP with their oxygen uptake preference, particularly in the hypoxic core. We examined the number of times a residue was modified within the group of proteins detected in each layer (Figure 5, Figures S6–19). Not surprisingly, methionine, owing to its high reactivity with hydroxyl radicals, was the most abundant modified residue in all three layers. Methionine is a class 2 residue that can take oxygen from H_2_O_2_ and O_2_ so modification of this residue in the core is not necessarily indicative of the effect of hypoxia on FPOP modification. However, spheroid-FPOP was able to modify eight of the class 3 residues, which almost exclusively take oxygen from O_2_. Of these residues, Asp, Glu, Lys, Leu, Pro, Gln, and Val were modified in all three layers of the spheroid (Figure 4). The lone exception was Ile, which is only modified in the outer layer. In the cases of Asp, Glu, and Leu, a higher number of modifications were observed in the hypoxic core than the other layers. Phe and Gln have similar numbers of modified residues in the inner layer and core. This indicates the capability of spheroid-FPOP to modify O_2_-dependent residues even in the hypoxic spheroid core. Class 1 residues Arg, Phe, and His were also modified by spheroid-FPOP. The ability of spheroid-FPOP to modify class 3 residues could be due to the addition of H_2_O_2_. Endogenous catalase catalyzes the decomposition of H_2_O_2_ to water and oxygen, thus generating the oxygen required for class 3 residues to be modified.

## Conclusion

In this work, we describe the development of spheroid-FPOP, a new structural proteomics platform to interrogate the heterogeneous tumor microenvironment. With some adjustments to the established PIXY platform, the method was successful in modifying hundreds of proteins throughout the spheroid. Interestingly, oxygen-dependent amino acids were modified in the hypoxic core of the spheroids. Failure to modify these amino acids would severely limit the applications of spheroid-FPOP. Serial trypsinization allowed for some spatial resolution of modified proteins. Here, we collected three layers, but a higher number of distinct layers can be separated to increase the resolution. The ability to analyze proteins in the core separately from the outer layer postlabeling increases the application of spheroid-FPOP to studying the effects of the oxygen, nutrient, and proliferation gradients in spheroids.

Despite the potential of this novel method, there are some constraints. For example, the 1 min incubation time for complete diffusion can lead to high background oxidation. In the control samples, where the spheroids were not exposed to laser irradiation, the outer, inner, and core layers had 597, 802, and 942 modified proteins, respectively. After subtraction of the background oxidation, 319, 244, and 249 modified proteins were in the outer, inner, and core layers, respectively. A reduced H_2_O_2_ incubation time would limit the background oxidation possibly leading to a higher yield of FPOP-modified proteins. Further optimization of the method is needed to determine whether a shorter incubation time can be used for spheroid-FPOP. An additional constraint is the use of a UV laser that has a reduced penetration depth. The presence of collagen in tissue samples reduces the penetration depth of UV lasers.^38^ Type 1 collagen is the major structural protein of ECM in the tumor microenvironment.^43^ To recreate this in spheroid models, cells are embedded within collagen-containing hydrogel. The spheroids used in this FPOP study were not embedded in a collagen-containing hydrogel but rather self-assembled in an agarose-based meniscus. Owing to this, the collagen content in the ECM of these spheroids is reduced. This lower collagen content aided the labeling of core proteins which required a laser penetration depth of at least 500 μm. The use of a UV laser for FPOP does limit the applications for three-dimensional model systems. Lastly, a low number of modified amino acids per protein was observed in this data set. This precludes the ability to correlated residue labeling to solvent accessible surface area to determine whether spheroid-FPOP accurately probes solvent accessibility. Further optimizations to the method need to be performed to increase the number of modified residues. Nonetheless, this work demonstrates the potential of spheroid-FPOP in probing protein structures and interactions within a native tumor environment.

## Supporting Information Available

Additional figures and tables as noted in the text (PDF)
