# Supplementary material for: Development of Spheroid-FPOP: An In-Cell Protein Footprinting Method for 3D Tumor Spheroids
Source: J Am Soc Mass Spectrom. 2023 Jan 26;34(3):417–25. doi: 10.1021/jasms.2c00307 (PMC9983004; doi:10.1021/jasms.2c00307)
Supplement: Supplementary file 1 — js2c00307_si_001.pdf [file js2c00307_si_001.pdf]

Supporting Information

Development of Spheroid-FPOP: An In-Cell Protein Footprinting Method for 3D Tumor Spheroids

Raquel Shortt,† Yijia Wang,‡ Amanda Hummon,‡, Lisa M. Jones*§

†Department of Pharmaceutical Sciences, University of Maryland, Baltimore, MD 21201, United States

‡Department of Chemistry and Biochemistry, The Ohio State University, Columbus, OH 43210, United States

§Department of Chemistry and Biochemistry, University of California San Diego, La Jolla, CA 92093, United States

*Correspondence to:

Lisa M. Jones

Department of Chemistry and Biochemistry

University of California San Diego, La Jolla, CA 92093

Phone number: 858-534-6455

Email: lijones@ucsd.edu

Table of Contents

Figure S1: Identification of LysoPC(16:0) by tandem MS with a standard. 4

Table S1: Parent and fragments detected to confirm identification of LysoPC(16:0) including accuracy in ppm 5

Figure S2: HCT116 Spheroid 6

Figure S3: Total Number of Protein Modified for 100 mM H_2O_2 A. BR1 B. BR2 7

Figure S4. Total Number of Protein Modified for 200 mM H_2O_2 BR1 and BR2. A. BR1 B. BR2 8

Table S2: Total unique proteins and their associated Uniprot pathways for 100 mM H_2O_2 that were modified by FPOP ..9

Table S3: Total unique proteins modified by FPOP using 200 mM H_2O_2....89

Figure S5: Tandem Mass Spectra of a Modified Peptide on Protein Q9P0K7 from the Outer Layer. B- and y- ions are visualized along with a +16 FPOP Modification on Glutamine. 123

Figure S6: Tandem Mass Spectra of a Modified Peptide on Protein P10809 from the Outer Layer. B- and y- ions are visualized along with a +16 FPOP Modification on Valine. 124

Figure S7: Tandem Mass Spectra of a Modified Peptide on Protein O60814 from the Outer Layer. B- and y- ions are visualized along with a +16 FPOP Modification on Isoleucine. 125

Figure S8: Tandem Mass Spectra of a Modified Peptide on Protein P49257 from the Inner Layer. B- and y- ions are visualized along with a +16 FPOP Modification on Proline. 126

Figure S9: Tandem Mass Spectra of a Modified Peptide on Protein O43707 from the Inner Layer. B- and y- ions are visualized along with a +16 FPOP Modification on Leucine. 127

Figure S10: Tandem Mass Spectra of a Modified Peptide on Protein Q9Y5S9 from the Inner Layer. B- and y- ions are visualized along with a +16 FPOP Modification on Arginine. 128

Figure S11: Tandem Mass Spectra of a Modified Peptide on Protein P52306 from the Inner Layer. B- and y- ions are visualized along with a +16 FPOP Modification on Asparagine. 129

Figure S12: Tandem Mass Spectra of a Modified Peptide on Protein Q8NBX0 from the Inner Layer. B- and y- ions are visualized along with a +14 FPOP Modification on Lysine. 130

Figure S13: Tandem Mass Spectra of a Modified Peptide on Protein P08238 from the Inner Layer. B- and y- ions are visualized along with a +16 FPOP Modification on Alanine. 131
Figure S14: Tandem Mass Spectra of a Modified Peptide on Protein P35998 from the Inner Layer. B- and y- ions are visualized along with a +16 FPOP Modification on Phenylalanine. 132
Figure S15: Tandem Mass Spectra of a Modified Peptide on Protein P07602 from the Core Layer. B- and y- ions are visualized along with a +16 FPOP Modification on Methionine. 133
Figure S16: Tandem Mass Spectra of a Modified Peptide on Protein P10809 from the Core Layer. B- and y- ions are visualized along with a -10 FPOP Modification on Histidine. 134
Figure S17: Tandem Mass Spectra of a Modified Peptide on Protein P10809 from the Core Layer. B- and y- ions are visualized along with a +16 FPOP Modification on Aspartic Acid. 135
Figure S18: Tandem Mass Spectra of a Modified Peptide on Protein P10809 from the Core Layer. B- and y- ions are visualized along with a -30 FPOP Modification on Glutamic Acid. 136

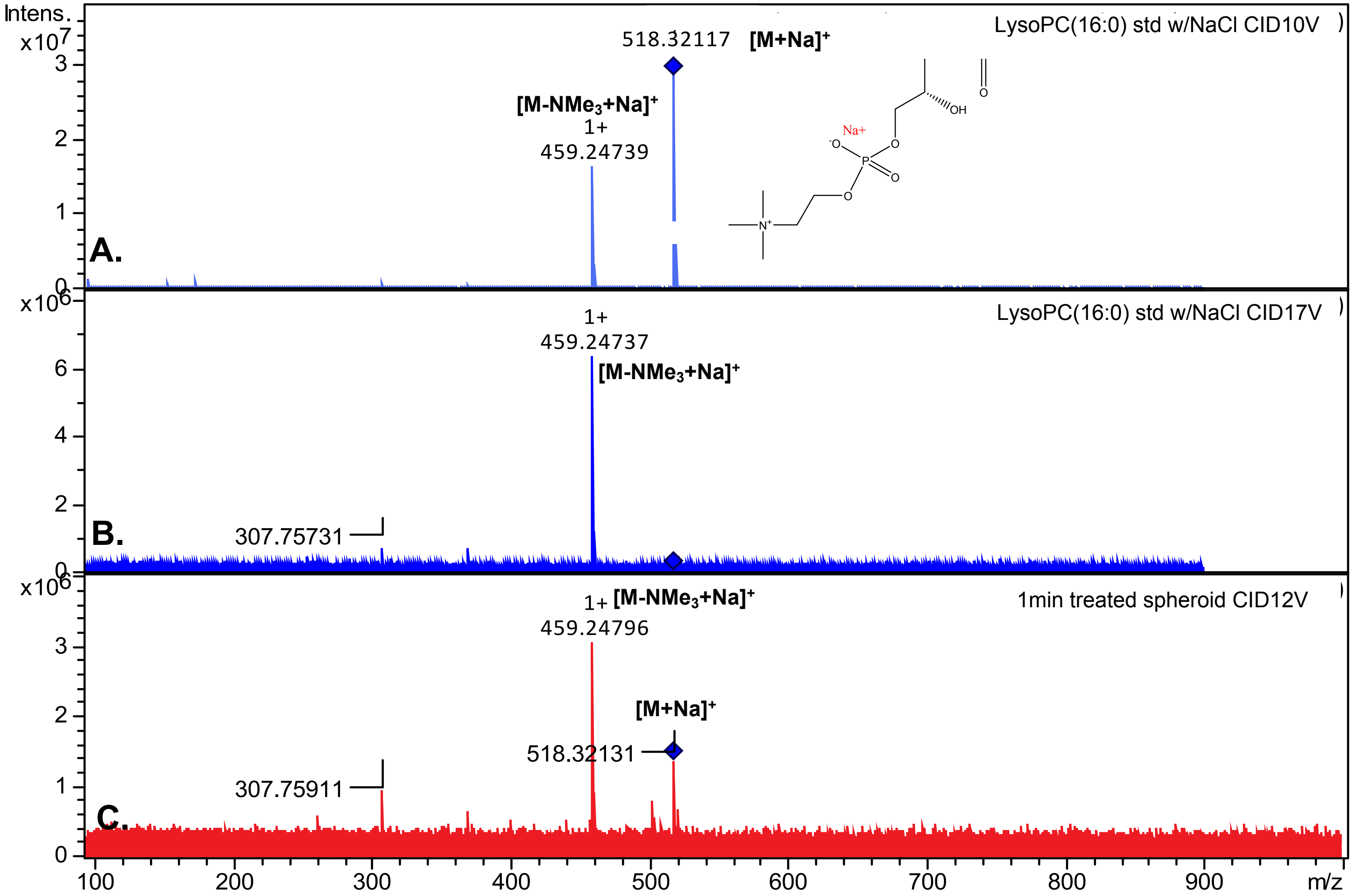

Figure S1: Identification of LysoPC(16:0) by tandem MS with a standard. Isolation window was set at 518.32 *m/z*. A) Standard solution with NaCl, collision energy 10eV; B) Standard solution with NaCl, collision energy 17eV; C) 1-minute treated spheroid sample, collision energy 12eV. The parent peak was detected at 518.3213 *m/z* in the spheroid sample and 518.3211 *m/z* in the standard sample, Δppm 0.4. Two matched fragments were detected at 459.2480 m/z and

307.7591 *m/z* in the spheroid sample and 459.2474 *m/z* and 307.7571 *m/z* in the standard solution, with Δppm 1.3 and 6.5 separately.

	Parent peak	Fragments			
Spheroid sample	518.3213	459.2480	369.3460	307.7591	261.4907
Std w/ NaCl	518.3211	459.2474	369.2572	307.7571	261.4799
Δppm	0.4	1.3	240	6.5	41

Table S1: Parent and fragments detected to confirm identification of LysoPC(16:0) including accuracy in ppm

Extent of Modification of Common Proteins

Master Protein Accession	Outer	Inner	Core
O14950	0.0089	0.1715	0.4934
O60506	0.9507	0.0715	0.4480
O60664	0.1447	0.0321	0.0604
P02545	0.0141	0.0124	0.4932
P05787	0.1558	0.0178	0.0067
P06748	0.0004	0.0025	0.0017
P07437	0.2100	0.0126	0.0062
P07900	0.0370	0.1135	0.7031
P08238	0.1261	0.0615	0.0454
P08727	0.2384	0.0005	0.0023
P0DMV9	0.0119	0.0198	0.0712
P0DP25	0.0231	0.0198	0.0388
P10809	0.0045	0.0033	0.0112
P13073	0.0109	0.0381	0.0293
P14625	0.2698	0.0226	0.0454
P18859	0.0032	0.0037	0.0290
P22307	0.2594	0.2946	0.2460
P27797	0.1273	0.2041	0.1066
P30044	0.5728	0.1082	0.5124
P60709	0.2503	0.0021	0.0080
P62805	0.0079	0.0005	0.0043
P62937	0.0017	0.0463	0.0001
P67936	0.0183	0.0031	0.0043
P68032	0.0004	0.0041	0.0090
P68371	0.0725	0.0136	0.0052
Q09666	0.3543	0.0338	0.2733
Q13885	0.2100	0.0591	0.0058

Q6DN03	0.5017	0.0040	0.0003
Q8NC51	0.0026	0.0209	0.0499
Q92597	0.0072	0.0010	0.0017
Q99497	0.0843	0.0919	0.2751
Q9BUF5	0.6078	0.5825	0.3507
P04350	0.6260	0.2103	0.2712
O60814	0.0030	0.0002	0.0024

Table S2. Global Level Oxidation on Proteins Modified in All Three Spheroid Layers

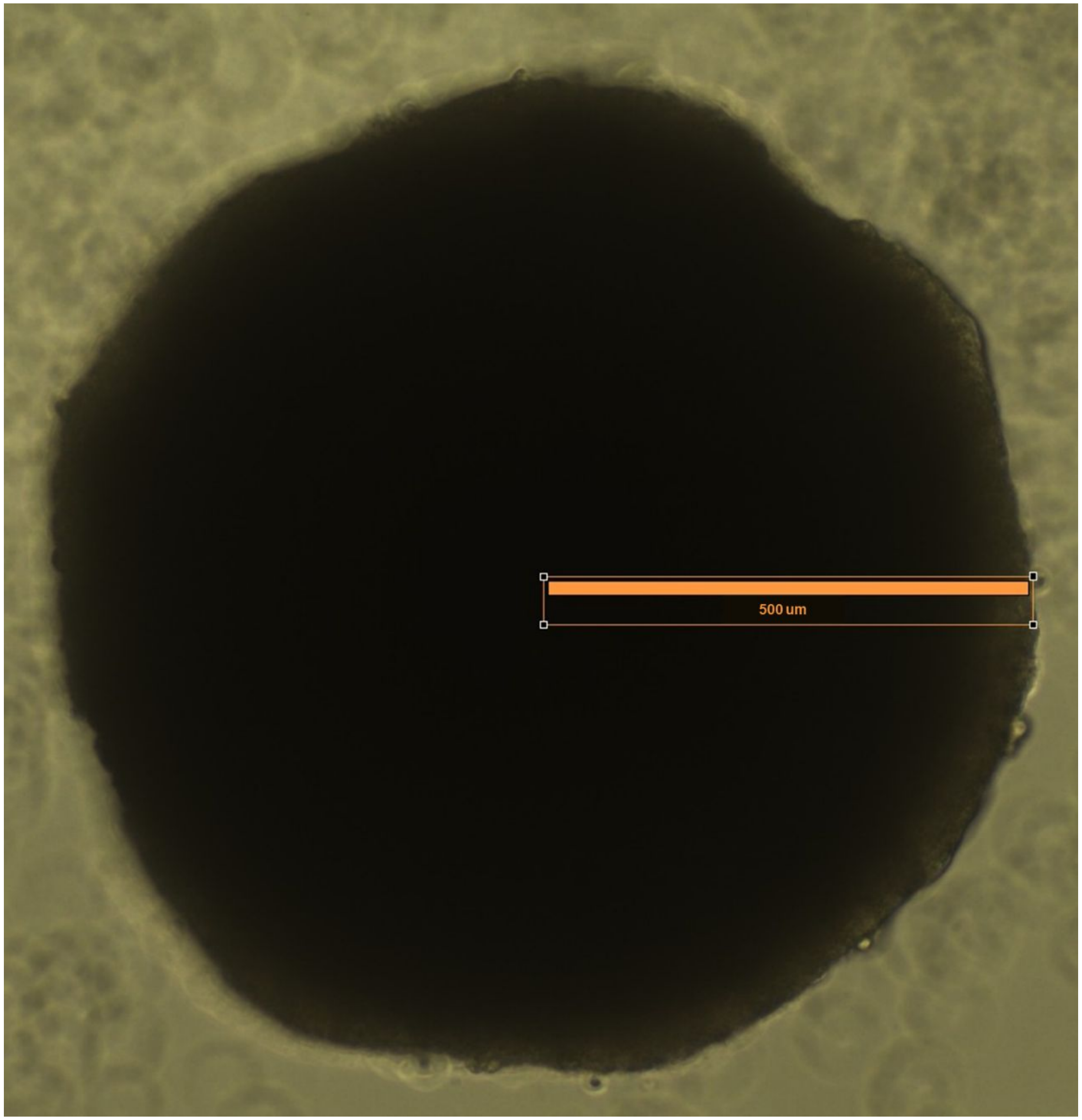

Figure S2: Image of a HCT116 Spheroid Taken on a Confocal Microscope

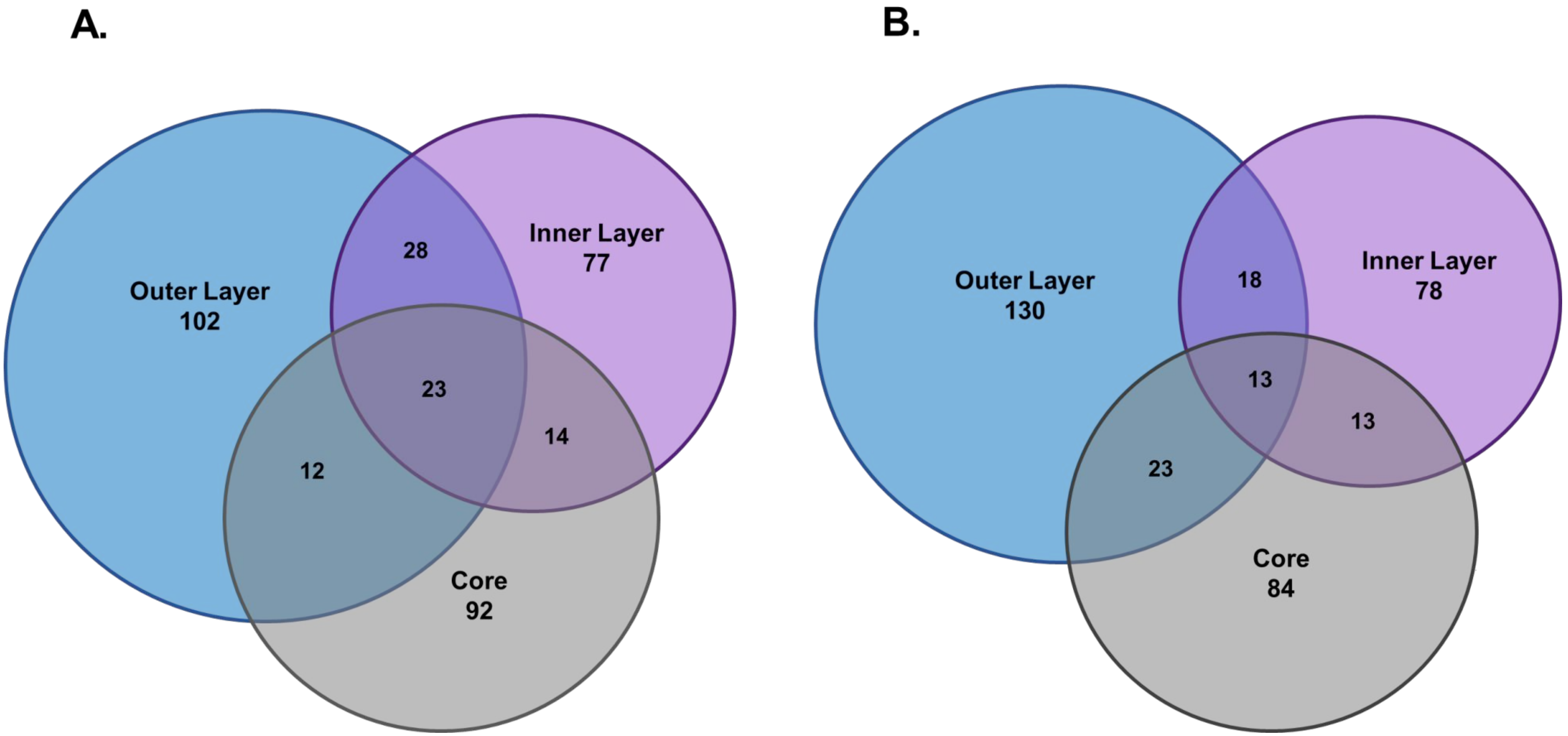

Figure S3: Total Number of Protein Modified for 100 mM H_2O_2 A. BR1 B. BR2

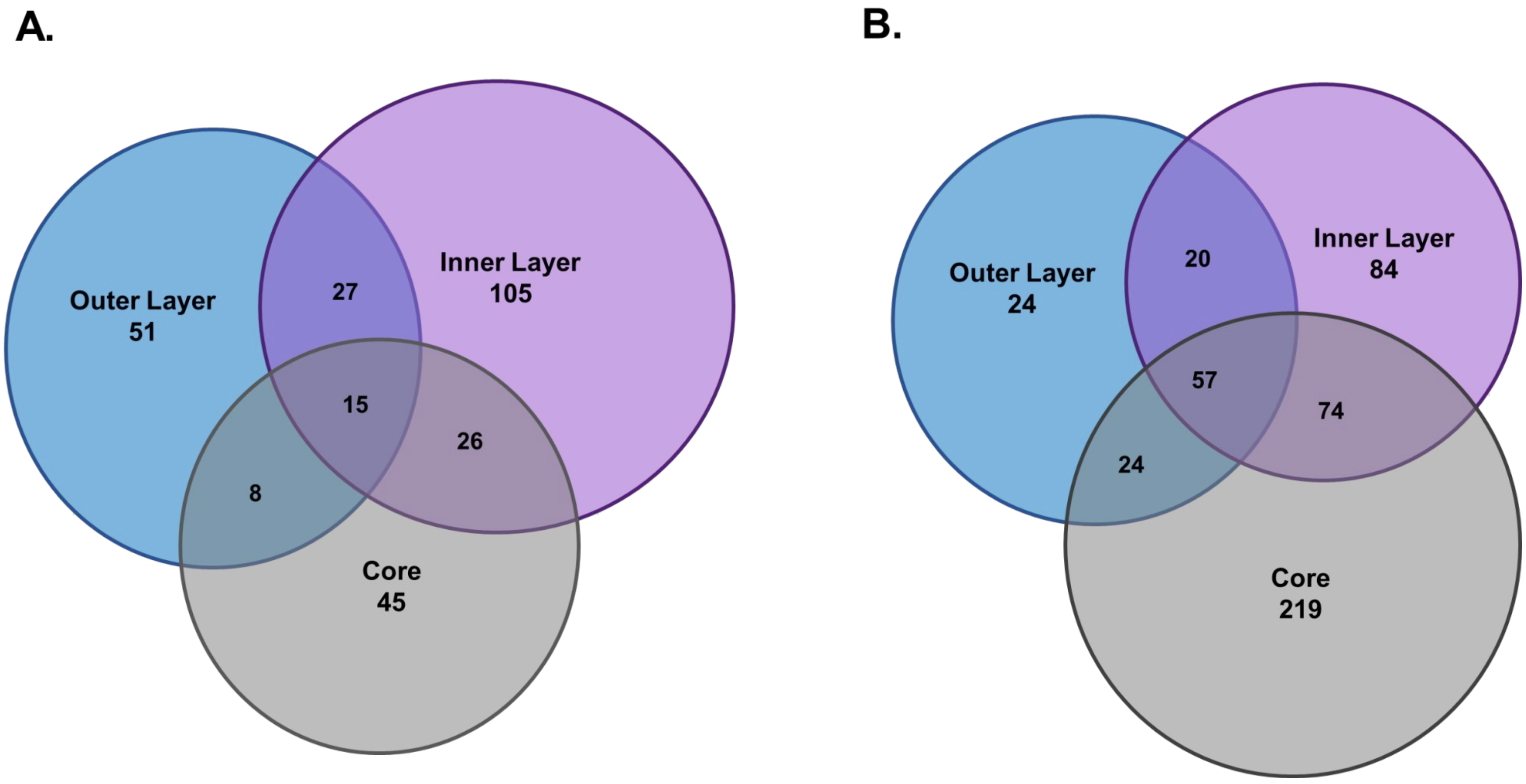

Figure S4. Total Number of Protein Modified for 200 mM H_2O_2 BR1 and BR2. A. BR1 B. BR2

Entry	Protein names	Pathway
A0A075B6N1	T cell receptor beta variable 19	
A6NCM1	IQ and AAA domain-containing protein 1-like (IQ and AAA domain-containing protein 1 pseudogene 1)	
A6NDY2	Putative protein FAM90A10	
A6NET4	Olfactory receptor 5K3	
A6NHR9	Structural maintenance of chromosomes flexible hinge domain-containing protein 1 (SMC hinge domain-containing protein 1) (EC 3.6.1.-)	
B7ZAP0	Rab GTPase-activating protein 1-like, isoform 10	
O00139	Kinesin-like protein KIF2A (Kinesin-2) (hK2)	
O00555	Voltage-dependent P/Q-type calcium channel subunit alpha-1A (Brain calcium channel I) (BI) (Calcium channel, L type, alpha-1 polypeptide isoform 4) (Voltage-gated calcium channel subunit alpha Cav2.1)	
O14618	Copper chaperone for superoxide dismutase (Superoxide dismutase copper chaperone)	

Entry	Protein names	Pathway
O14745	Na(+)/H(+) exchange regulatory cofactor NHE-RF1 (NHERF-1) (Ezrin-radixin-moesin-binding phosphoprotein 50) (EBP50) (Regulatory cofactor of Na(+)/H(+) exchanger) (Sodium-hydrogen exchanger regulatory factor 1) (Solute carrier family 9 isoform A3 regulatory factor 1)	
O14950	Myosin regulatory light chain 12B (MLC-2A) (MLC-2) (Myosin regulatory light chain 2-B, smooth muscle isoform) (Myosin regulatory light chain 20 kDa) (MLC20) (Myosin regulatory light chain MRLC2) (SHUJUN-1)	
O15041	Semaphorin-3E	
P30837	Aldehyde dehydrogenase X, mitochondrial (EC 1.2.1.3) (Aldehyde dehydrogenase 5) (Aldehyde dehydrogenase family 1 member B1)	PATHWAY: Alcohol metabolism; ethanol degradation; acetate from ethanol: step 2/2.
O43157	Plexin-B1 (Semaphorin receptor SEP)	
O43314	Inositol hexakisphosphate and diphosphoinositol-pentakisphosphate kinase 2 (EC 2.7.4.21) (EC 2.7.4.24) (Diphosphoinositol pentakisphosphate kinase 2) (Histidine acid phosphatase domain-containing protein 1) (InsP6 and PP-IP5 kinase 2) (VIP1 homolog 2) (hsVIP2)	

Entry	Protein names	Pathway
O43504	Ragulator complex protein LAMTOR5 (Hepatitis B virus X-interacting protein) (HBV X-interacting protein) (HBX-interacting protein) (Late endosomal/lysosomal adaptor and MAPK and MTOR activator 5)	
P26440	Isovaleryl-CoA dehydrogenase, mitochondrial (IVD) (EC 1.3.8.4) (Butyryl-CoA dehydrogenase) (EC 1.3.8.1)	PATHWAY: Amino-acid degradation; L-leucine degradation; (S)-3-hydroxy-3-methylglutaryl-CoA from 3-isovaleryl-CoA: step 1/3.
O43865	S-adenosylhomocysteine hydrolase-like protein 1 (DC-expressed AHCY-like molecule) (IP(3)Rs binding protein released with IP(3)) (IRBIT) (Putative adenosylhomocysteinase 2) (S-adenosyl-L-homocysteine hydrolase 2) (AdoHcyase 2)	
O60506	Heterogeneous nuclear ribonucleoprotein Q (hnRNP Q) (Glycine- and tyrosine-rich RNA-binding protein) (GRY-RBP) (NS1-associated protein 1) (Synaptotagmin-binding, cytoplasmic RNA-interacting protein)	
O60664	Perilipin-3 (47 kDa mannose 6-phosphate receptor-binding protein) (47 kDa MPR-binding protein) (Cargo selection protein TIP47) (Mannose-6-phosphate receptor-binding protein 1) (Placental protein 17) (PP17)	

Entry	Protein names	Pathway
P35558	Phosphoenolpyruvate carboxykinase, cytosolic [GTP] (PEPCK-C) (EC 4.1.1.32) (Serine-protein kinase PCK1) (EC 2.7.11.-)	PATHWAY: Carbohydrate biosynthesis; gluconeogenesis. {ECO:0000269|PubMed:24863970, ECO:0000269|PubMed:26971250, ECO:0000269|PubMed:28216384, ECO:0000269|PubMed:30193097}.
O60814	Histone H2B type 1-K (H2B K) (HIRA-interacting protein 1)	
O60826	Coiled-coil domain-containing protein 22	
O75093	Slit homolog 1 protein (Slit-1) (Multiple epidermal growth factor-like domains protein 4) (Multiple EGF-like domains protein 4)	
O75143	Autophagy-related protein 13	
O75147	Obscurin-like protein 1	
O75155	Cullin-associated NEDD8-dissociated protein 2 (Cullin-associated and neddylation-dissociated protein 2) (Epididymis tissue protein Li 169) (TBP-interacting protein of 120 kDa B) (TBP-interacting protein 120B) (p120 CAND2)	
O75880	Protein SCO1 homolog, mitochondrial	
O94762	ATP-dependent DNA helicase Q5 (EC 3.6.4.12) (DNA helicase, RecQ-like type 5) (RecQ5) (RecQ protein-like 5)	

Entry	Protein names	Pathway
Q96G03	Phosphoglucomutase-2 (PGM 2) (EC 5.4.2.2) (Glucose phosphomutase 2) (Phosphodeoxyribomutase) (Phosphopentomutase) (EC 5.4.2.7)	PATHWAY: Carbohydrate degradation; 2-deoxy-D-ribose 1-phosphate degradation; D-glyceraldehyde 3-phosphate and acetaldehyde from 2-deoxy-alpha-D-ribose 1-phosphate: step 1/2. {ECO:0000305|PubMed:17804405}.
O95707	Ribonuclease P protein subunit p29 (hPOP4)	
P04075	Fructose-bisphosphate aldolase A (EC 4.1.2.13) (Lung cancer antigen NY-LU-1) (Muscle-type aldolase)	PATHWAY: Carbohydrate degradation; glycolysis; D-glyceraldehyde 3-phosphate and glycerone phosphate from D-glucose: step 4/4.
P04406	Glyceraldehyde-3-phosphate dehydrogenase (GAPDH) (EC 1.2.1.12) (Peptidyl-cysteine S-nitrosylase GAPDH) (EC 2.6.99.-)	PATHWAY: Carbohydrate degradation; glycolysis; pyruvate from D-glyceraldehyde 3-phosphate: step 1/5.

Entry	Protein names	Pathway
P01024	Complement C3 (C3 and PZP-like alpha-2-macroglobulin domain-containing protein 1) [Cleaved into: Complement C3 beta chain; C3-beta-c (C3bc); Complement C3 alpha chain; C3a anaphylatoxin; Acylation stimulating protein (ASP) (C3adesArg); Complement C3b alpha' chain; Complement C3c alpha' chain fragment 1; Complement C3dg fragment; Complement C3g fragment; Complement C3d fragment; Complement C3f fragment; Complement C3c alpha' chain fragment 2]	
P02533	Keratin, type I cytoskeletal 14 (Cytokeratin-14) (CK-14) (Keratin-14) (K14)	
P02545	Prelamin-A/C [Cleaved into: Lamin-A/C (70 kDa lamin) (Renal carcinoma antigen NY-REN-32)]	
P02771	Alpha-fetoprotein (Alpha-1-fetoprotein) (Alpha-fetoglobulin)	
P00558	Phosphoglycerate kinase 1 (EC 2.7.2.3) (Cell migration-inducing gene 10 protein) (Primer recognition protein 2) (PRP 2)	PATHWAY: Carbohydrate degradation; glycolysis; pyruvate from D-glyceraldehyde 3-phosphate: step 2/5. {ECO:0000269|PubMed:30323285, ECO:0000269|PubMed:7391028}.

Entry	Protein names	Pathway
P04264	Keratin, type II cytoskeletal 1 (67 kDa cytokeratin) (Cytokeratin-1) (CK-1) (Hair alpha protein) (Keratin-1) (K1) (Type-II keratin Kb1)	
P04350	Tubulin beta-4A chain (Tubulin 5 beta) (Tubulin beta-4 chain)	
P06733	Alpha-enolase (EC 4.2.1.11) (2-phospho-D-glycerate hydro-lyase) (C-myc promoter-binding protein) (Enolase 1) (MBP-1) (MPB-1) (Non-neural enolase) (NNE) (Phosphopyruvate hydratase) (Plasminogen-binding protein)	PATHWAY: Carbohydrate degradation; glycolysis; pyruvate from D-glyceraldehyde 3-phosphate: step 4/5. {ECO:0000305|PubMed:1369209}.
P04437	T cell receptor alpha variable 29/delta variable 5	
P13073	Cytochrome c oxidase subunit 4 isoform 1, mitochondrial (Cytochrome c oxidase polypeptide IV) (Cytochrome c oxidase subunit IV isoform 1) (COX IV-1)	PATHWAY: Energy metabolism; oxidative phosphorylation. {ECO:0000250|UniProtKB:P00424}.
P20674	Cytochrome c oxidase subunit 5A, mitochondrial (Cytochrome c oxidase polypeptide Va)	PATHWAY: Energy metabolism; oxidative phosphorylation. {ECO:0000250|UniProtKB:P00427}.
P05783	Keratin, type I cytoskeletal 18 (Cell proliferation-inducing gene 46 protein) (Cytokeratin-18) (CK-18) (Keratin-18) (K18)	
P05787	Keratin, type II cytoskeletal 8 (Cytokeratin-8) (CK-8) (Keratin-8) (K8) (Type-II keratin Kb8)	
P06576	ATP synthase subunit beta, mitochondrial (EC 7.1.2.2) (ATP synthase F1 subunit beta)	

Entry	Protein names	Pathway
P06732	Creatine kinase M-type (EC 2.7.3.2) (Creatine kinase M chain) (Creatine phosphokinase M-type) (CPK-M) (M-CK)	
P00338	L-lactate dehydrogenase A chain (LDH-A) (EC 1.1.1.27) (Cell proliferation-inducing gene 19 protein) (LDH muscle subunit) (LDH-M) (Renal carcinoma antigen NY-REN-59)	PATHWAY: Fermentation; pyruvate fermentation to lactate; (S)-lactate from pyruvate: step 1/1.
P06748	Nucleophosmin (NPM) (Nucleolar phosphoprotein B23) (Nucleolar protein NO38) (Numatrin)	
P07237	Protein disulfide-isomerase (PDI) (EC 5.3.4.1) (Cellular thyroid hormone-binding protein) (Prolyl 4-hydroxylase subunit beta) (p55)	
P07437	Tubulin beta chain (Tubulin beta-5 chain)	
P07476	Involucrin	
P07478	Trypsin-2 (EC 3.4.21.4) (Anionic trypsinogen) (Serine protease 2) (Trypsin II)	
P07711	Procathepsin L (EC 3.4.22.15) (Cathepsin L1) (Major excreted protein) (MEP) [Cleaved into: Cathepsin L; Cathepsin L heavy chain; Cathepsin L light chain]	
P07900	Heat shock protein HSP 90-alpha (EC 3.6.4.10) (Heat shock 86 kDa) (HSP 86) (HSP86) (Lipopolysaccharide-associated protein 2) (LAP-2) (LPS-associated protein 2) (Renal carcinoma antigen NY-REN-38)	

Entry	Protein names	Pathway
P08238	Heat shock protein HSP 90-beta (HSP 90) (Heat shock 84 kDa) (HSP 84) (HSP84)	
P08574	Cytochrome c1, heme protein, mitochondrial (EC 7.1.1.8) (Complex III subunit 4) (Complex III subunit IV) (Cytochrome b-c1 complex subunit 4) (Ubiquinol-cytochrome-c reductase complex cytochrome c1 subunit) (Cytochrome c-1)	
P08575	Receptor-type tyrosine-protein phosphatase C (EC 3.1.3.48) (Leukocyte common antigen) (L-CA) (T200) (CD antigen CD45)	
P08727	Keratin, type I cytoskeletal 19 (Cytokeratin-19) (CK-19) (Keratin-19) (K19)	
P09455	Retinol-binding protein 1 (Cellular retinol-binding protein) (CRBP) (Cellular retinol-binding protein I) (CRBP-I)	
P0CG38	POTE ankyrin domain family member I	
P0CG39	POTE ankyrin domain family member J	
P0CJ78	Zinc finger protein 865	
P0DMV9	Heat shock 70 kDa protein 1B (Heat shock 70 kDa protein 2) (HSP70-2) (HSP70.2)	
P0DP25	Calmodulin-3	
P01889	HLA class I histocompatibility antigen, B alpha chain (Human leukocyte antigen B) (HLA-B)	

Entry	Protein names	Pathway
P10809	60 kDa heat shock protein, mitochondrial (EC 5.6.1.7) (60 kDa chaperonin) (Chaperonin 60) (CPN60) (Heat shock protein 60) (HSP-60) (Hsp60) (HuCHA60) (Mitochondrial matrix protein P1) (P60 lymphocyte protein)	
P11021	Endoplasmic reticulum chaperone BiP (EC 3.6.4.10) (78 kDa glucose-regulated protein) (GRP-78) (Binding-immunoglobulin protein) (BiP) (Heat shock protein 70 family protein 5) (HSP70 family protein 5) (Heat shock protein family A member 5) (Immunoglobulin heavy chain-binding protein)	
P11142	Heat shock cognate 71 kDa protein (EC 3.6.4.10) (Heat shock 70 kDa protein 8) (Lipopolysaccharide-associated protein 1) (LAP-1) (LPS-associated protein 1)	
P11277	Spectrin beta chain, erythrocytic (Beta-I spectrin)	
P11532	Dystrophin	

Entry	Protein names	Pathway
P51659	Peroxisomal multifunctional enzyme type 2 (MFE-2) (17-beta-hydroxysteroid dehydrogenase 4) (17-beta-HSD 4) (D-bifunctional protein) (DBP) (Multifunctional protein 2) (MFP-2) (Short chain dehydrogenase/reductase family 8C member 1) [Cleaved into: (3R)-hydroxyacyl-CoA dehydrogenase (EC 1.1.1.n12); Enoyl-CoA hydratase 2 (EC 4.2.1.107) (EC 4.2.1.119) (3-alpha,7-alpha,12-alpha-trihydroxy-5-beta-cholest-24-enoyl-CoA hydratase)]	PATHWAY: Lipid metabolism; fatty acid beta-oxidation. {ECO:0000269|PubMed:10706581, ECO:0000269|PubMed:15060085, ECO:0000269|PubMed:9089413, ECO:0000269|PubMed:9482850}.
P13631	Retinoic acid receptor gamma (RAR-gamma) (Nuclear receptor subfamily 1 group B member 3)	
P13674	Prolyl 4-hydroxylase subunit alpha-1 (4-PH alpha-1) (EC 1.14.11.2) (Procollagen-proline,2-oxoglutarate-4-dioxygenase subunit alpha-1)	
P13994	Probable splicing factor YJU2B (Coiled-coil domain-containing protein 130)	
P14625	Endoplasmin (94 kDa glucose-regulated protein) (GRP-94) (Heat shock protein 90 kDa beta member 1) (Tumor rejection antigen 1) (gp96 homolog)	

Entry	Protein names	Pathway
P15822	Zinc finger protein 40 (Cirhin interaction protein) (CIRIP) (Gate keeper of apoptosis-activating protein) (GAAP) (Human immunodeficiency virus type I enhancer-binding protein 1) (HIV-EP1) (Major histocompatibility complex-binding protein 1) (MBP-1) (Positive regulatory domain II-binding factor 1) (PRDII-BF1)	
P16070	CD44 antigen (CDw44) (Epican) (Extracellular matrix receptor III) (ECMR-III) (GP90 lymphocyte homing/adhesion receptor) (HUTCH-I) (Heparan sulfate proteoglycan) (Hermes antigen) (Hyaluronate receptor) (Phagocytic glycoprotein 1) (PGP-1) (Phagocytic glycoprotein I) (PGP-I) (CD antigen CD44)	
P17035	Zinc finger protein 28 (Zinc finger protein KOX24)	
P18859	ATP synthase-coupling factor 6, mitochondrial (ATPase subunit F6) (ATP synthase peripheral stalk subunit F6)	
Q13085	Acetyl-CoA carboxylase 1 (ACC1) (EC 6.4.1.2) (Acetyl-Coenzyme A carboxylase alpha) (ACC-alpha)	PATHWAY: Lipid metabolism; malonyl-CoA biosynthesis; malonyl-CoA from acetyl-CoA: step 1/1. {ECO:0000305|PubMed:20457939, ECO:0000305|PubMed:20952656}.

Entry	Protein names	Pathway
P21281	V-type proton ATPase subunit B, brain isoform (V-ATPase subunit B 2) (Endomembrane proton pump 58 kDa subunit) (HO57) (Vacuolar proton pump subunit B 2)	
P21399	Cytoplasmic aconitate hydratase (Aconitase) (EC 4.2.1.3) (Citrate hydro-lyase) (Ferritin repressor protein) (Iron regulatory protein 1) (IRP1) (Iron-responsive element-binding protein 1) (IRE-BP 1)	
P22033	Methylmalonyl-CoA mutase, mitochondrial (MCM) (EC 5.4.99.2) (Methylmalonyl-CoA isomerase)	
P22307	Sterol carrier protein 2 (SCP-2) (Acetyl-CoA C-myristoyltransferase) (EC 2.3.1.155) (Non-specific lipid-transfer protein) (NSL-TP) (Propanoyl-CoA C-acyltransferase) (EC 2.3.1.176) (SCP-2/3-oxoacyl-CoA thiolase) (SCP-2/thiolase) (EC 2.3.1.16) (SCP-chi) (SCPX) (Sterol carrier protein X) (SCP-X) (Straight-chain acyl-CoA oxidase) (SCOX)	
P23528	Cofilin-1 (18 kDa phosphoprotein) (p18) (Cofilin, non-muscle isoform)	
P24821	Tenascin (TN) (Cytotactin) (GMEM) (GP 150-225) (Glioma-associated-extracellular matrix antigen) (Hexabrachion) (JI) (Myotendinous antigen) (Neuronectin) (Tenascin-C) (TN-C)	

Entry	Protein names	Pathway
P25092	Guanylyl cyclase C (GC-C) (EC 4.6.1.2) (Heat-stable enterotoxin receptor) (STA receptor) (hSTAR) (Intestinal guanylate cyclase)	
P25705	ATP synthase subunit alpha, mitochondrial (ATP synthase F1 subunit alpha)	
P25940	Collagen alpha-3(V) chain	
P05166	Propionyl-CoA carboxylase beta chain, mitochondrial (PCCase subunit beta) (EC 6.4.1.3) (Propanoyl-CoA:carbon dioxide ligase subunit beta)	PATHWAY: Metabolic intermediate metabolism; propanoyl-CoA degradation; succinyl-CoA from propanoyl-CoA: step 1/3. {ECO:0000269|PubMed:15890657, ECO:0000269|PubMed:6765947}.
P26641	Elongation factor 1-gamma (EF-1-gamma) (eEF-1B gamma)	
P27348	14-3-3 protein theta (14-3-3 protein T-cell) (14-3-3 protein tau) (Protein HS1)	
P27797	Calreticulin (CRP55) (Calregulin) (Endoplasmic reticulum resident protein 60) (ERp60) (HACBP) (grp60)	
P27824	Calnexin (IP90) (Major histocompatibility complex class I antigen-binding protein p88) (p90)	
P29274	Adenosine receptor A2a	

Entry	Protein names	Pathway
P29320	Ephrin type-A receptor 3 (EC 2.7.10.1) (EPH-like kinase 4) (EK4) (hEK4) (HEK) (Human embryo kinase) (Tyrosine-protein kinase TYRO4) (Tyrosine-protein kinase receptor ETK1) (Eph-like tyrosine kinase 1)	
P29374	AT-rich interactive domain-containing protein 4A (ARID domain-containing protein 4A) (Retinoblastoma-binding protein 1) (RBBP-1)	
P29692	Elongation factor 1-delta (EF-1-delta) (Antigen NY-CO-4)	
P30041	Peroxiredoxin-6 (EC 1.11.1.27) (1-Cys peroxiredoxin) (1-Cys PRX) (24 kDa protein) (Acidic calcium-independent phospholipase A2) (aiPLA2) (EC 3.1.1.4) (Antioxidant protein 2) (Glutathione-dependent peroxiredoxin) (Liver 2D page spot 40) (Lysophosphatidylcholine acyltransferase 5) (LPC acyltransferase 5) (LPCAT-5) (Lyso-PC acyltransferase 5) (EC 2.3.1.23) (Non-selenium glutathione peroxidase) (NSGPx) (Red blood cells page spot 12)	
P30044	Peroxiredoxin-5, mitochondrial (EC 1.11.1.24) (Alu corepressor 1) (Antioxidant enzyme B166) (AOEB166) (Liver tissue 2D-page spot 71B) (PLP) (Peroxiredoxin V) (Prx-V) (Peroxisomal antioxidant enzyme) (TPx type VI) (Thioredoxin peroxidase PMP20) (Thioredoxin-dependent peroxiredoxin 5)	

Entry	Protein names	Pathway
P04439	HLA class I histocompatibility antigen, A alpha chain (Human leukocyte antigen A) (HLA-A)	
P34897	Serine hydroxymethyltransferase, mitochondrial (SHMT) (EC 2.1.2.1) (Glycine hydroxymethyltransferase) (Serine methylase)	PATHWAY: One-carbon metabolism; tetrahydrofolate interconversion. {ECO:0000305|PubMed:25619277, ECO:0000305|PubMed:33015733}.
P31946	14-3-3 protein beta/alpha (Protein 1054) (Protein kinase C inhibitor protein 1) (KCIP-1) [Cleaved into: 14-3-3 protein beta/alpha, N-terminally processed]	
P31947	14-3-3 protein sigma (Epithelial cell marker protein 1) (Stratifin)	
P33991	DNA replication licensing factor MCM4 (EC 3.6.4.12) (CDC21 homolog) (P1-CDC21)	
P36551	Oxygen-dependent coproporphyrinogen-III oxidase, mitochondrial (COX) (Coprogen oxidase) (Coproporphyrinogenase) (EC 1.3.3.3)	PATHWAY: Porphyrin-containing compound metabolism; protoporphyrin-IX biosynthesis; protoporphyrinogen-IX from coproporphyrinogen-III (O2 route): step 1/1. {ECO:0000269|PubMed:8159699}.

Entry	Protein names	Pathway
Q9NYU2	UDP-glucose:glycoprotein glucosyltransferase 1 (UGT1) (hUGT1) (EC 2.4.1.-) (UDP--Glc:glycoprotein glucosyltransferase) (UDP-glucose ceramide glucosyltransferase-like 1)	PATHWAY: Protein modification; protein glycosylation. {ECO:0000269|PubMed:10694380, ECO:0000269|PubMed:15815621}.
P35579	Myosin-9 (Cellular myosin heavy chain, type A) (Myosin heavy chain 9) (Myosin heavy chain, non-muscle IIa) (Non-muscle myosin heavy chain A) (NMMHC-A) (Non-muscle myosin heavy chain IIa) (NMMHC II-a) (NMMHC-IIA)	
P35637	RNA-binding protein FUS (75 kDa DNA-pairing protein) (Oncogene FUS) (Oncogene TLS) (POMp75) (Translocated in liposarcoma protein)	
O15294	UDP-N-acetylglucosamine--peptide N-acetylglucosaminyltransferase 110 kDa subunit (EC 2.4.1.255) (O-GlcNAc transferase subunit p110) (O-linked N-acetylglucosamine transferase 110 kDa subunit) (OGT)	PATHWAY: Protein modification; protein glycosylation. {ECO:0000269|PubMed:15361863, ECO:0000269|PubMed:21240259, ECO:0000269|PubMed:21285374, ECO:0000269|PubMed:23103939, ECO:0000269|PubMed:26678539}.

Entry	Protein names	Pathway
P38935	DNA-binding protein SMUBP-2 (EC 3.6.4.12) (EC 3.6.4.13) (ATP-dependent helicase IGHMBP2) (Glial factor 1) (GF-1) (Immunoglobulin mu-binding protein 2)	
P40200	T-cell surface protein tactile (Cell surface antigen CD96) (T cell-activated increased late expression protein) (CD antigen CD96)	
P41236	Protein phosphatase inhibitor 2 (IPP-2)	
P42345	Serine/threonine-protein kinase mTOR (EC 2.7.11.1) (FK506-binding protein 12-rapamycin complex-associated protein 1) (FKBP12-rapamycin complex-associated protein) (Mammalian target of rapamycin) (mTOR) (Mechanistic target of rapamycin) (Rapamycin and FKBP12 target 1) (Rapamycin target protein 1)	
P43320	Beta-crystallin B2 (Beta-B2 crystallin) (Beta-crystallin Bp)	
P43403	Tyrosine-protein kinase ZAP-70 (EC 2.7.10.2) (70 kDa zeta-chain associated protein) (Syk-related tyrosine kinase)	
P45974	Ubiquitin carboxyl-terminal hydrolase 5 (EC 3.4.19.12) (Deubiquitinating enzyme 5) (Isopeptidase T) (Ubiquitin thioesterase 5) (Ubiquitin-specific-processing protease 5)	
P48735	Isocitrate dehydrogenase [NADP], mitochondrial (IDH) (EC 1.1.1.42) (ICD-M) (IDP) (NADP(+)-specific ICDH) (Oxalosuccinate decarboxylase)	

Entry	Protein names	Pathway
P51148	Ras-related protein Rab-5C (EC 3.6.5.2) (L1880) (RAB5L)	
P04844	Dolichyl-diphosphooligosaccharide--protein glycosyltransferase subunit 2 (Dolichyl-diphosphooligosaccharide--protein glycosyltransferase 63 kDa subunit) (RIBIIR) (Ribophorin II) (RPN-II) (Ribophorin-2)	PATHWAY: Protein modification; protein glycosylation. {ECO:0000269|PubMed:31831667}.
P51685	C-C chemokine receptor type 8 (C-C CKR-8) (CC-CKR-8) (CCR-8) (CC chemokine receptor CHEMR1) (CMKBRL2) (Chemokine receptor-like 1) (CKR-L1) (GPR-CY6) (GPRCY6) (TER1) (CD antigen CDw198)	
P51858	Hepatoma-derived growth factor (HDGF) (High mobility group protein 1-like 2) (HMG-1L2)	
P52701	DNA mismatch repair protein Msh6 (hMSH6) (G/T mismatch-binding protein) (GTBP) (GTMBP) (MutS protein homolog 6) (MutS-alpha 160 kDa subunit) (p160)	
P53621	Coatomer subunit alpha (Alpha-coat protein) (Alpha-COP) (HEP-COP) (HEPCOP) [Cleaved into: Xenin (Xenopsin-related peptide); Proxenin]	
P55771	Paired box protein Pax-9	
P55895	V(D)J recombination-activating protein 2 (RAG-2)	
P56373	P2X purinoceptor 3 (P2X3) (ATP receptor) (Purinergic receptor)	

Entry	Protein names	Pathway
P59923	Zinc finger protein 445 (ZFP445) (Zinc finger protein 168) (Zinc finger protein with KRAB and SCAN domains 15)	
P60709	Actin, cytoplasmic 1 (Beta-actin) [Cleaved into: Actin, cytoplasmic 1, N-terminally processed]	
P61604	10 kDa heat shock protein, mitochondrial (Hsp10) (10 kDa chaperonin) (Chaperonin 10) (CPN10) (Early-pregnancy factor) (EPF)	
P61978	Heterogeneous nuclear ribonucleoprotein K (hnRNP K) (Transformation up-regulated nuclear protein) (TUNP)	
P61981	14-3-3 protein gamma (Protein kinase C inhibitor protein 1) (KCIP-1) [Cleaved into: 14-3-3 protein gamma, N-terminally processed]	
P62258	14-3-3 protein epsilon (14-3-3E)	
P62310	U6 snRNA-associated Sm-like protein LSm3	
P62805	Histone H4	
P62937	Peptidyl-prolyl cis-trans isomerase A (PPIase A) (EC 5.2.1.8) (Cyclophilin A) (Cyclosporin A-binding protein) (Rotamase A) [Cleaved into: Peptidyl-prolyl cis-trans isomerase A, N-terminally processed]	
P62979	Ubiquitin-40S ribosomal protein S27a (Ubiquitin carboxyl extension protein 80) [Cleaved into: Ubiquitin; 40S ribosomal protein S27a (Small ribosomal subunit protein eS31)]	

Entry	Protein names	Pathway
P63104	14-3-3 protein zeta/delta (Protein kinase C inhibitor protein 1) (KCIP-1)	
P67936	Tropomyosin alpha-4 chain (TM30p1) (Tropomyosin-4)	
P68032	Actin, alpha cardiac muscle 1 (Alpha-cardiac actin) [Cleaved into: Actin, alpha cardiac muscle 1, intermediate form]	
P68371	Tubulin beta-4B chain (Tubulin beta-2 chain) (Tubulin beta-2C chain)	
P69905	Hemoglobin subunit alpha (Alpha-globin) (Hemoglobin alpha chain)	
Q00610	Clathrin heavy chain 1 (Clathrin heavy chain on chromosome 17) (CLH-17)	
Q01082	Spectrin beta chain, non-erythrocytic 1 (Beta-II spectrin) (Fodrin beta chain) (Spectrin, non-erythroid beta chain 1)	
Q03701	CCAAT/enhancer-binding protein zeta (CCAAT-box-binding transcription factor) (CBF) (CCAAT-binding factor)	
O60762	Dolichol-phosphate mannosyltransferase subunit 1 (EC 2.4.1.83) (Dolichol-phosphate mannose synthase subunit 1) (DPM synthase subunit 1) (Dolichyl-phosphate beta-D-mannosyltransferase subunit 1) (Mannose-P-dolichol synthase subunit 1) (MPD synthase subunit 1)	PATHWAY: Protein modification; protein glycosylation. {ECO:0000305|PubMed:10835346}.
Q04917	14-3-3 protein eta (Protein AS1)	

Entry	Protein names	Pathway
Q06830	Peroxiredoxin-1 (EC 1.11.1.24) (Natural killer cell-enhancing factor A) (NKEF-A) (Proliferation-associated gene protein) (PAG) (Thioredoxin peroxidase 2) (Thioredoxin-dependent peroxide reductase 2) (Thioredoxin-dependent peroxiredoxin 1)	
Q07866	Kinesin light chain 1 (KLC 1)	
Q09666	Neuroblast differentiation-associated protein AHNAK (Desmoyokin)	
Q12799	Deleted.	
Q12913	Receptor-type tyrosine-protein phosphatase eta (Protein-tyrosine phosphatase eta) (R-PTP-eta) (EC 3.1.3.48) (Density-enhanced phosphatase 1) (DEP-1) (HPTP eta) (Protein-tyrosine phosphatase receptor type J) (R-PTP-J) (CD antigen CD148)	
O43766	Lipoyl synthase, mitochondrial (EC 2.8.1.8) (Lipoate synthase) (LS) (Lip-syn) (Lipoic acid synthase)	PATHWAY: Protein modification; protein lipoylation via endogenous pathway; protein N(6)-(lipoyl)lysine from octanoyl-[acyl-carrier-protein]: step 2/2. {ECO:0000255|HAMAP-Rule:MF_03123}.
Q13129	Zinc finger protein Rlf (Rearranged L-myc fusion gene protein) (Zn-15-related protein)	

Entry	Protein names	Pathway
Q13263	Transcription intermediary factor 1-beta (TIF1-beta) (E3 SUMO-protein ligase TRIM28) (EC 2.3.2.27) (KRAB-associated protein 1) (KAP-1) (KRAB-interacting protein 1) (KRIP-1) (Nuclear corepressor KAP-1) (RING finger protein 96) (RING-type E3 ubiquitin transferase TIF1-beta) (Tripartite motif-containing protein 28)	PATHWAY: Protein modification; protein sumoylation.
Q13459	Unconventional myosin-IXb (Unconventional myosin-9b)	
Q13562	Neurogenic differentiation factor 1 (NeuroD) (NeuroD1) (Class A basic helix-loop-helix protein 3) (bHLHa3)	
Q13885	Tubulin beta-2A chain (Tubulin beta class IIa)	
O95071	E3 ubiquitin-protein ligase UBR5 (EC 2.3.2.26) (E3 ubiquitin-protein ligase, HECT domain-containing 1) (HECT-type E3 ubiquitin transferase UBR5) (Hyperplastic discs protein homolog) (hHYD) (Progestin-induced protein)	PATHWAY: Protein modification; protein ubiquitination.
Q14565	Meiotic recombination protein DMC1/LIM15 homolog	
Q14582	Max dimerization protein 4 (Max dimerizer 4) (Class C basic helix-loop-helix protein 12) (bHLHc12) (Max-associated protein 4) (Max-interacting transcriptional repressor MAD4)	

Entry	Protein names	Pathway
Q14679	Tubulin monoglutamylase TTLL4 (EC 6.3.2.-) (Protein monoglutamylase TTLL4) (Tubulin--tyrosine ligase-like protein 4)	
Q14980	Nuclear mitotic apparatus protein 1 (Nuclear matrix protein-22) (NMP-22) (Nuclear mitotic apparatus protein) (NuMA protein) (SP-H antigen)	
Q14993	Collagen alpha-1(XIX) chain (Collagen alpha-1(Y) chain)	
Q14C86	GTPase-activating protein and VPS9 domain-containing protein 1 (GAPex-5) (Rab5-activating protein 6)	
Q15059	Bromodomain-containing protein 3 (RING3-like protein)	
Q15075	Early endosome antigen 1 (Endosome-associated protein p162) (Zinc finger FYVE domain-containing protein 2)	
Q14527	Helicase-like transcription factor (EC 2.3.2.27) (EC 3.6.4.-) (DNA-binding protein/plasminogen activator inhibitor 1 regulator) (HIP116) (RING finger protein 80) (RING-type E3 ubiquitin transferase HLTF) (SWI/SNF-related matrix-associated actin-dependent regulator of chromatin subfamily A member 3) (Sucrose nonfermenting protein 2-like 3)	PATHWAY: Protein modification; protein ubiquitination.
Q15436	Protein transport protein Sec23A (hSec23A) (SEC23-related protein A)	

Entry	Protein names	Pathway
Q15437	Protein transport protein Sec23B (hSec23B) (SEC23-related protein B)	
Q15782	Chitinase-3-like protein 2 (Chondrocyte protein 39) (YKL-39)	
Q15878	Voltage-dependent R-type calcium channel subunit alpha-1E (Brain calcium channel II) (BII) (Calcium channel, L type, alpha-1 polypeptide, isoform 6) (Voltage-gated calcium channel subunit alpha Cav2.3)	
Q16623	Syntaxin-1A (Neuron-specific antigen HPC-1)	
Q16629	Serine/arginine-rich splicing factor 7 (Splicing factor 9G8) (Splicing factor, arginine/serine-rich 7)	
Q16666	Gamma-interferon-inducible protein 16 (Ifi-16) (Interferon-inducible myeloid differentiation transcriptional activator)	
Q16881	Thioredoxin reductase 1, cytoplasmic (TR) (EC 1.8.1.9) (Gene associated with retinoic and interferon-induced mortality 12 protein) (GRIM-12) (Gene associated with retinoic and IFN-induced mortality 12 protein) (KM-102-derived reductase-like factor) (Peroxidase TXNRD1) (EC 1.11.1.2) (Thioredoxin reductase TR1)	
P10321	HLA class I histocompatibility antigen, C alpha chain (HLA-C) (HLA-Cw) (Human leukocyte antigen C)	

Entry	Protein names	Pathway
Q2VIQ3	Chromosome-associated kinesin KIF4B (Chromokinesin-B)	
Q3KR37	Protein Aster-B (GRAM domain-containing protein 1B)	
Q3MIT2	tRNA pseudouridine synthase Pus10 (Hup10) (EC 5.4.99.25) (Coiled-coil domain-containing protein 139) (tRNA pseudouridine 55 synthase) (Psi55 synthase) (tRNA pseudouridylate synthase) (tRNA-uridine isomerase)	
Q4VNC0	Probable cation-transporting ATPase 13A5 (EC 7.2.2.-) (P5-ATPase isoform 5)	
Q562R1	Beta-actin-like protein 2 (Kappa-actin)	
Q58FF7	Putative heat shock protein HSP 90-beta-3 (Heat shock protein 90-beta c) (Heat shock protein 90Bc)	
Q5JTH9	RRP12-like protein	
Q5S007	Leucine-rich repeat serine/threonine-protein kinase 2 (EC 2.7.11.1) (EC 3.6.5.-) (Dardarin)	
Q5TBA9	Protein furry homolog	
Q5TZJ5	Spermatogenesis-associated protein 31A1 (Protein FAM75A1)	
Q5VVW2	GTPase-activating Rap/Ran-GAP domain-like protein 3	
Q68DK7	Male-specific lethal 1 homolog (MSL-1) (Male-specific lethal 1-like 1) (MSL1-like 1) (Male-specific lethal-1 homolog 1)	

Entry	Protein names	Pathway
Q6DKK2	Tetratricopeptide repeat protein 19, mitochondrial (TPR repeat protein 19)	
Q6DN03	Putative histone H2B type 2-C (H2B-clustered histone 20 pseudogene) (Histone H2B.t) (H2B/t)	
Q6FI13	Histone H2A type 2-A (H2A-clustered histone 18) (H2A-clustered histone 19) (Histone H2A.2) (Histone H2A/o)	
Q6IA17	Single Ig IL-1-related receptor (Single Ig IL-1R-related molecule) (Single immunoglobulin domain-containing IL1R-related protein) (Toll/interleukin-1 receptor 8) (TIR8)	
Q6IEE7	Transmembrane protein 132E	
Q6J272	Protein FAM166A	
Q6NSZ9	Zinc finger and SCAN domain-containing protein 25 (Zinc finger protein 498)	
Q6NUI6	Chondroadherin-like protein	
Q6NZI2	Caveolae-associated protein 1 (Cavin-1) (Polymerase I and transcript release factor)	
Q6P4F2	Ferredoxin-2, mitochondrial (Adrenodoxin-like protein) (Ferredoxin-1-like protein)	
Q6Q788	Apolipoprotein A-V (Apo-AV) (ApoA-V) (Apolipoprotein A5) (Regeneration-associated protein 3)	
Q6UXD1	Histidine-rich carboxyl terminus protein 1	

Entry	Protein names	Pathway
Q6V1P9	Protocadherin-23 (Cadherin-27) (Cadherin-like protein CDHJ) (Cadherin-like protein VR8) (Protein dachsous homolog 2) (Protocadherin PCDHJ)	
Q6ZRP7	Sulfhydryl oxidase 2 (EC 1.8.3.2) (Neuroblastoma-derived sulfhydryl oxidase) (Quiescin Q6-like protein 1)	
Q6ZS30	Neurobeachin-like protein 1 (Amyotrophic lateral sclerosis 2 chromosomal region candidate gene 16 protein) (Amyotrophic lateral sclerosis 2 chromosomal region candidate gene 17 protein)	
Q6ZU80	Centrosomal protein of 128 kDa (Cep128)	
Q7Z3H4	Sterile alpha motif domain-containing protein 7 (SAM domain-containing protein 7)	
Q7Z4S6	Kinesin-like protein KIF21A (Kinesin-like protein KIF2) (Renal carcinoma antigen NY-REN-62)	
Q7Z699	Sprouty-related, EVH1 domain-containing protein 1 (Spred-1) (hSpred1)	
Q7Z7M0	Multiple epidermal growth factor-like domains protein 8 (Multiple EGF-like domains protein 8) (Epidermal growth factor-like protein 4) (EGF-like protein 4)	
Q86UE4	Protein LYRIC (3D3/LYRIC) (Astrocyte elevated gene-1 protein) (AEG-1) (Lysine-rich CEACAM1 co-isolated protein) (Metadherin) (Metastasis adhesion protein)	

Entry	Protein names	Pathway
Q86UV5	Ubiquitin carboxyl-terminal hydrolase 48 (EC 3.4.19.12) (Deubiquitinating enzyme 48) (Ubiquitin thioesterase 48) (Ubiquitin-specific peptidase 48) (Ubiquitin-specific protease 48) (Ubiquitin-specific-processing protease 48)	
Q86VI1	Exocyst complex component 3-like protein (Protein Jiangli)	
Q86Y22	Collagen alpha-1(XXIII) chain	
Q8IVF2	Protein AHNAK2	
Q8IWR1	Tripartite motif-containing protein 59 (RING finger protein 104) (Tumor suppressor TSBF-1)	
Q8IYP2	Serine protease 58 (EC 3.4.21.4) (Trypsin-X3)	
Q8IZT6	Abnormal spindle-like microcephaly-associated protein (Abnormal spindle protein homolog) (Asp homolog)	
Q8N141	Zinc finger protein 82 homolog (Zfp-82) (Zinc finger protein 545)	
Q8N1F8	Serine/threonine-protein kinase 11-interacting protein (LKB1-interacting protein 1)	
Q8N2C7	Protein unc-80 homolog	
Q8N7W2	BEN domain-containing protein 7	
Q8NBS9	Thioredoxin domain-containing protein 5 (Endoplasmic reticulum resident protein 46) (ER protein 46) (ERp46) (Thioredoxin-like protein p46)	

Entry	Protein names	Pathway
Q8NC51	Plasminogen activator inhibitor 1 RNA-binding protein (PAI1 RNA-binding protein 1) (PAI-RBP1) (SERPINE1 mRNA-binding protein 1)	
Q8NC69	BTB/POZ domain-containing protein KCTD6 (KCASH3 protein) (Potassium channel tetramerization domain-containing protein 6)	PATHWAY: Protein modification; protein ubiquitination.
Q8ND23	Capping protein, Arp2/3 and myosin-I linker protein 3 (Capping protein regulator and myosin 1 linker protein 3) (Leucine-rich repeat-containing protein 16B)	
Q8NDA2	Hemicentin-2	
Q8NDI1	EH domain-binding protein 1	
Q8NE65	Zinc finger protein 738	
Q8NFC6	Biorientation of chromosomes in cell division protein 1-like 1	
Q8NFP9	Neurobeachin (Lysosomal-trafficking regulator 2) (Protein BCL8B)	
Q8NHQ1	Centrosomal protein of 70 kDa (Cep70) (p10-binding protein)	
Q8NHQ8	Ras association domain-containing protein 8 (Carcinoma-associated protein HOJ-1)	
Q8NHY3	GAS2-like protein 2 (GAS2-related protein on chromosome 17) (Growth arrest-specific protein 2-like 2)	

Entry	Protein names	Pathway
Q8TBX8	Phosphatidylinositol 5-phosphate 4-kinase type-2 gamma (EC 2.7.1.149) (Phosphatidylinositol 5-phosphate 4-kinase type II gamma) (PI(5)P 4-kinase type II gamma) (PIP4KII-gamma)	
Q8TC20	Cancer-associated gene 1 protein (CAGE-1) (Cancer/testis antigen 3) (CT3)	
Q8TCQ1	E3 ubiquitin-protein ligase MARCHF1 (EC 2.3.2.27) (Membrane-associated RING finger protein 1) (Membrane-associated RING-CH protein I) (MARCH-I) (RING finger protein 171) (RING-type E3 ubiquitin transferase MARCHF1)	PATHWAY: Protein modification; protein ubiquitination.
Q8TEQ0	Sorting nexin-29 (RUN domain-containing protein 2A)	
Q8TF45	Zinc finger protein 418	
Q8WTT2	Nucleolar complex protein 3 homolog (NOC3 protein homolog) (Factor for adipocyte differentiation 24) (NOC3-like protein) (Nucleolar complex-associated protein 3-like protein)	
Q8WXA3	RUN and FYVE domain-containing protein 2 (Rab4-interacting protein related)	
Q92597	Protein NDRG1 (Differentiation-related gene 1 protein) (DRG-1) (N-myc downstream-regulated gene 1 protein) (Nickel-specific induction protein Cap43) (Reducing agents and tunicamycin-responsive protein) (RTP) (Rit42)	

Entry	Protein names	Pathway
Q92797	Symplekin	
Q92817	Envoplakin (210 kDa cornified envelope precursor protein) (210 kDa paraneoplastic pemphigus antigen) (p210)	
Q92851	Caspase-10 (CASP-10) (EC 3.4.22.63) (Apoptotic protease Mch-4) (FAS-associated death domain protein interleukin-1B-converting enzyme 2) (FLICE2) (ICE-like apoptotic protease 4) [Cleaved into: Caspase-10 subunit p23/17; Caspase-10 subunit p12]	
Q92922	SWI/SNF complex subunit SMARCC1 (BRG1-associated factor 155) (BAF155) (SWI/SNF complex 155 kDa subunit) (SWI/SNF-related matrix-associated actin-dependent regulator of chromatin subfamily C member 1)	
Q92945	Far upstream element-binding protein 2 (FUSE-binding protein 2) (KH type-splicing regulatory protein) (KSRP) (p75)	
Q969G5	Caveolae-associated protein 3 (Cavin-3) (Protein kinase C delta-binding protein) (Serum deprivation response factor-related gene product that binds to C-kinase) (hSRBC)	
Q969Q5	Ras-related protein Rab-24	

Entry	Protein names	Pathway
Q96A70	Antizyme inhibitor 2 (AzI2) (Arginine decarboxylase) (ADC) (ARGDC) (Ornithine decarboxylase-like protein) (ODC-like protein) (ornithine decarboxylase paralog) (ODC-p)	
Q96AV8	Transcription factor E2F7 (E2F-7)	
Q96BJ3	Axin interactor, dorsalization-associated protein (Axin interaction partner and dorsalization antagonist)	
Q96DN5	TBC1 domain family member 31 (WD repeat-containing protein 67)	
Q9C040	Tripartite motif-containing protein 2 (EC 2.3.2.27) (E3 ubiquitin-protein ligase TRIM2) (RING finger protein 86) (RING-type E3 ubiquitin transferase TRIM2)	PATHWAY: Protein modification; protein ubiquitination.
Q96GA3	Protein LTV1 homolog	
Q96JA3	Pleckstrin homology domain-containing family A member 8 (PH domain-containing family A member 8) (Phosphatidylinositol-four-phosphate adapter protein 2) (FAPP-2) (Phosphoinositol 4-phosphate adapter protein 2) (hFAPP2) (Serologically defined breast cancer antigen NY-BR-86)	
Q96MG7	Non-structural maintenance of chromosomes element 3 homolog (Non-SMC element 3 homolog) (Hepatocellular carcinoma-associated protein 4) (MAGE-G1 antigen) (Melanoma-associated antigen G1) (Necdin-like protein 2)	

Entry	Protein names	Pathway
Q96P65	Pyroglutamylated RF-amide peptide receptor (AQ27) (G-protein coupled receptor 103) (Orexigenic neuropeptide QRFP receptor) (SP9155)	
Q96PH1	NADPH oxidase 5 (EC 1.6.3.-)	
Q96PY6	Serine/threonine-protein kinase Nek1 (EC 2.7.11.1) (Never in mitosis A-related kinase 1) (NimA-related protein kinase 1) (Renal carcinoma antigen NY-REN-55)	
Q96ST8	Centrosomal protein of 89 kDa (Cep89) (Centrosomal protein 123) (Cep123) (Coiled-coil domain-containing protein 123)	
Q99497	Parkinson disease protein 7 (Maillard deglycase) (Oncogene DJ1) (Parkinsonism-associated deglycase) (Protein DJ-1) (DJ-1) (Protein/nucleic acid deglycase DJ-1) (EC 3.1.2.-) (EC 3.5.1.-) (EC 3.5.1.124)	
Q99525	Histone H4-like protein type G (H4-clustered histone 7)	
Q99547	M-phase phosphoprotein 6	
Q99856	AT-rich interactive domain-containing protein 3A (ARID domain-containing protein 3A) (B-cell regulator of IgH transcription) (Bright) (Dead ringer-like protein 1) (E2F-binding protein 1)	
Q99928	Gamma-aminobutyric acid receptor subunit gamma-3 (GABA(A) receptor subunit gamma-3)	

Entry	Protein names	Pathway
Q9BQ75	Protein CMSS1 (Cms1 ribosomal small subunit homolog)	
Q9BUF5	Tubulin beta-6 chain (Tubulin beta class V)	
Q9BUK6	Protein misato homolog 1	
Q9BXT4	Tudor domain-containing protein 1 (Cancer/testis antigen 41.1) (CT41.1)	
Q9BXX2	Ankyrin repeat domain-containing protein 30B (Serologically defined breast cancer antigen NY-BR-1.1)	
Q9BZW7	Testis-specific gene 10 protein (Testis development protein NYD-SP7)	
Q15369	Elongin-C (EloC) (Elongin 15 kDa subunit) (RNA polymerase II transcription factor SIII subunit C) (SIII p15) (Transcription elongation factor B polypeptide 1)	PATHWAY: Protein modification; protein ubiquitination. {ECO:0000269|PubMed:26138980, ECO:0000269|PubMed:29775578, ECO:0000269|PubMed:29779948, ECO:0000269|PubMed:30166453}.
Q9C0A1	Zinc finger homeobox protein 2 (Zinc finger homeodomain protein 2) (ZFH-2)	
Q9C0C2	182 kDa tankyrase-1-binding protein	
Q9H0B3	IQ domain-containing protein N	
Q9H0U4	Ras-related protein Rab-1B (EC 3.6.5.2)	
Q9H2E6	Semaphorin-6A (Semaphorin VIA) (Sema VIA) (Semaphorin-6A-1) (SEMA6A-1)	

Entry	Protein names	Pathway
Q9H6R4	Nucleolar protein 6 (Nucleolar RNA-associated protein) (Nrap)	
Q9H892	Tetratricopeptide repeat protein 12 (TPR repeat protein 12)	
Q9HCG8	Pre-mRNA-splicing factor CWC22 homolog (Nucampholin homolog) (fSAPb)	
Q9HCH5	Synaptotagmin-like protein 2 (Breast cancer-associated antigen SGA-72M) (Exophilin-4)	
Q9HCN8	Stromal cell-derived factor 2-like protein 1 (SDF2-like protein 1) (PWP1-interacting protein 8)	
Q9NQC3	Reticulon-4 (Foocen) (Neurite outgrowth inhibitor) (Nogo protein) (Neuroendocrine-specific protein) (NSP) (Neuroendocrine-specific protein C homolog) (RTN-x) (Reticulon-5)	
Q9NR19	Acetyl-coenzyme A synthetase, cytoplasmic (EC 6.2.1.1) (Acetate--CoA ligase) (Acetyl-CoA synthetase) (ACS) (AceCS) (Acetyl-CoA synthetase 1) (AceCS1) (Acyl-CoA synthetase short-chain family member 2) (Acyl-activating enzyme) (Propionate--CoA ligase) (EC 6.2.1.17)	
Q9NRB3	Carbohydrate sulfotransferase 12 (EC 2.8.2.5) (Chondroitin 4-O-sulfotransferase 2) (Chondroitin 4-sulfotransferase 2) (C4ST-2) (C4ST2) (Sulfotransferase Hlo)	
Q9NRC6	Spectrin beta chain, non-erythrocytic 5 (Beta-V spectrin)	

Entry	Protein names	Pathway
Q9NRY4	Rho GTPase-activating protein 35 (Glucocorticoid receptor DNA-binding factor 1) (Glucocorticoid receptor repression factor 1) (GRF-1) (Rho GAP p190A) (p190-A)	
Q9NS69	Mitochondrial import receptor subunit TOM22 homolog (hTom22) (1C9-2) (Translocase of outer membrane 22 kDa subunit homolog)	
Q9NUL7	Probable ATP-dependent RNA helicase DDX28 (EC 3.6.4.13) (Mitochondrial DEAD box protein 28)	
Q04760	Lactoylglutathione lyase (EC 4.4.1.5) (Aldoketomutase) (Glyoxalase I) (Glx I) (Ketone-aldehyde mutase) (Methylglyoxalase) (S-D-lactoylglutathione methylglyoxal lyase)	PATHWAY: Secondary metabolite metabolism; methylglyoxal degradation; (R)-lactate from methylglyoxal: step 1/2.
Q9NZ09	Ubiquitin-associated protein 1 (UBAP-1) (Nasopharyngeal carcinoma-associated gene 20 protein)	
Q9NZJ4	Sacsin (DnaJ homolog subfamily C member 29) (DNAJC29)	
Q9P0U4	CXXC-type zinc finger protein 1 (CpG-binding protein) (PHD finger and CXXC domain-containing protein 1)	
Q9P2E3	NFX1-type zinc finger-containing protein 1	
Q9P2H5	Ubiquitin carboxyl-terminal hydrolase 35 (EC 3.4.19.12) (Deubiquitinating enzyme 35) (Ubiquitin thioesterase 35) (Ubiquitin-specific-processing protease 35)	

Entry	Protein names	Pathway
Q9UGI9	5'-AMP-activated protein kinase subunit gamma-3 (AMPK gamma3) (AMPK subunit gamma-3)	
Q9UHD2	Serine/threonine-protein kinase TBK1 (EC 2.7.11.1) (NF-kappa-B-activating kinase) (T2K) (TANK-binding kinase 1)	
Q9UHV7	Mediator of RNA polymerase II transcription subunit 13 (Activator-recruited cofactor 250 kDa component) (ARC250) (Mediator complex subunit 13) (Thyroid hormone receptor-associated protein 1) (Thyroid hormone receptor-associated protein complex 240 kDa component) (Trap240) (Vitamin D3 receptor-interacting protein complex component DRIP250) (DRIP250)	
Q9UJ99	Cadherin-22 (Pituitary and brain cadherin) (PB-cadherin)	
Q9UKK9	ADP-sugar pyrophosphatase (EC 3.6.1.13) (8-oxo-dGDP phosphatase) (EC 3.6.1.58) (Nuclear ATP-synthesis protein NUDIX5) (EC 2.7.7.96) (Nucleoside diphosphate-linked moiety X motif 5) (Nudix motif 5) (hNUDT5) (YSA1H)	
Q9ULD9	Zinc finger protein 608 (Renal carcinoma antigen NY-REN-36)	
Q9ULR3	Protein phosphatase 1H (EC 3.1.3.16)	
Q9UMS6	Synaptopodin-2 (Genethonin-2) (Myopodin)	

Entry	Protein names	Pathway
Q9UPM8	AP-4 complex subunit epsilon-1 (AP-4 adaptor complex subunit epsilon) (Adaptor-related protein complex 4 subunit epsilon-1) (Epsilon subunit of AP-4) (Epsilon-adaptin)	
Q9UPN3	Microtubule-actin cross-linking factor 1, isoforms 1/2/3/5 (620 kDa actin-binding protein) (ABP620) (Actin cross-linking family protein 7) (Macrophin-1) (Trabeculin-alpha)	
Q9UQV4	Lysosome-associated membrane glycoprotein 3 (LAMP-3) (Lysosomal-associated membrane protein 3) (DC-lysosome-associated membrane glycoprotein) (DC LAMP) (Protein TSC403) (CD antigen CD208)	
Q9Y230	RuvB-like 2 (EC 3.6.4.12) (48 kDa TATA box-binding protein-interacting protein) (48 kDa TBP-interacting protein) (51 kDa erythrocyte cytosolic protein) (ECP-51) (INO80 complex subunit J) (Repressing pontin 52) (Reptin 52) (TIP49b) (TIP60-associated protein 54-beta) (TAP54-beta)	
Q9Y253	DNA polymerase eta (EC 2.7.7.7) (RAD30 homolog A) (Xeroderma pigmentosum variant type protein)	
Q9Y2H0	Disks large-associated protein 4 (DAP-4) (PSD-95/SAP90-binding protein 4) (SAP90/PSD-95-associated protein 4) (SAPAP-4)	

Entry	Protein names	Pathway
Q9Y3A2	Probable U3 small nucleolar RNA-associated protein 11 (U3 snoRNA-associated protein 11) (UTP11-like protein)	
Q9Y3C4	EKC/KEOPS complex subunit TPRKB (PRPK-binding protein) (TP53RK-binding protein)	
Q9Y597	BTB/POZ domain-containing protein KCTD3 (Renal carcinoma antigen NY-REN-45)	
Q9Y5H9	Protocadherin alpha-2 (PCDH-alpha-2)	
Q9Y6Q5	AP-1 complex subunit mu-2 (AP-mu chain family member mu1B) (Adaptor protein complex AP-1 subunit mu-2) (Adaptor-related protein complex 1 subunit mu-2) (Clathrin assembly protein complex 1 mu-2 medium chain 2) (Golgi adaptor HA1/AP1 adaptin mu-2 subunit) (Mu-adaptin 2) (Mu1B-adaptin)	
Q9Y6V0	Protein piccolo (Aczonin)	
A6NFN3	RNA binding protein fox-1 homolog 3 (Fox-1 homolog C) (Neuronal nuclei antigen) (NeuN antigen)	
A6NKC0	Putative protein FAM90A7	
B2RC85	Radial spoke head 10 homolog B2	
B8ZZ34	Protein shisa-8 (Shisa family member 8)	
H0YKK7	Putative golgin subfamily A member 6-like protein 19	

Entry	Protein names	Pathway
O14576	Cytoplasmic dynein 1 intermediate chain 1 (Cytoplasmic dynein intermediate chain 1) (Dynein intermediate chain 1, cytosolic) (DH IC-1)	
O14908	PDZ domain-containing protein GIPC1 (GAIP C-terminus-interacting protein) (RGS-GAIP-interacting protein) (RGS19-interacting protein 1) (Synectin) (Tax interaction protein 2) (TIP-2)	
O15417	Trinucleotide repeat-containing gene 18 protein (Long CAG trinucleotide repeat-containing gene 79 protein)	
O15504	Nucleoporin NUP42 (NLP-1) (NUP42 homolog) (Nucleoporin hCG1) (Nucleoporin-42) (Nucleoporin-like protein 2)	
O43169	Cytochrome b5 type B (Cytochrome b5 outer mitochondrial membrane isoform)	
O43399	Tumor protein D54 (hD54) (Tumor protein D52-like 2)	
O43567	E3 ubiquitin-protein ligase RNF13 (EC 2.3.2.27) (RING finger protein 13) (RING-type E3 ubiquitin transferase RNF13)	PATHWAY: Protein modification; protein ubiquitination.
O60934	Nibrin (Cell cycle regulatory protein p95) (Nijmegen breakage syndrome protein 1)	
O75145	Liprin-alpha-3 (Protein tyrosine phosphatase receptor type f polypeptide-interacting protein alpha-3) (PTPRF-interacting protein alpha-3)	

Entry	Protein names	Pathway
O75380	NADH dehydrogenase [ubiquinone] iron-sulfur protein 6, mitochondrial (Complex I-13kD-A) (CI-13kD-A) (NADH-ubiquinone oxidoreductase 13 kDa-A subunit)	
O75417	DNA polymerase theta (EC 2.7.7.7) (DNA polymerase eta)	
O75899	Gamma-aminobutyric acid type B receptor subunit 2 (GABA-B receptor 2) (GABA-B-R2) (GABA-BR2) (GABABR2) (Gb2) (G-protein coupled receptor 51) (HG20)	
O95613	Pericentrin (Kendrin) (Pericentrin-B)	
O95897	Noelin-2 (Olfactomedin-2)	
O95954	Formimidoyltransferase-cyclodeaminase (Formiminotransferase-cyclodeaminase) (FTCD) (LCHC1) [Includes: Glutamate formimidoyltransferase (EC 2.1.2.5) (Glutamate formiminotransferase) (Glutamate formyltransferase); Formimidoyltetrahydrofolate cyclodeaminase (EC 4.3.1.4) (Formiminotetrahydrofolate cyclodeaminase)]	PATHWAY: Amino-acid degradation; L-histidine degradation into L-glutamate; L-glutamate from N-formimidoyl-L-glutamate (transferase route): step 1/1. {ECO:0000269|PubMed:12815595}.

Entry	Protein names	Pathway
P01019	Angiotensinogen (Serpin A8) [Cleaved into: Angiotensin-1 (Angiotensin 1-10) (Angiotensin I) (Ang I); Angiotensin-2 (Angiotensin 1-8) (Angiotensin II) (Ang II); Angiotensin-3 (Angiotensin 2-8) (Angiotensin III) (Ang III) (Des-Asp[1]-angiotensin II); Angiotensin-4 (Angiotensin 3-8) (Angiotensin IV) (Ang IV); Angiotensin 1-9; Angiotensin 1-7; Angiotensin 1-5; Angiotensin 1-4]	
P06753	Tropomyosin alpha-3 chain (Gamma-tropomyosin) (Tropomyosin-3) (Tropomyosin-5) (hTM5)	
P07108	Acyl-CoA-binding protein (ACBP) (Diazepam-binding inhibitor) (DBI) (Endozepine) (EP)	
P07602	Prosaposin (Proactivator polypeptide) [Cleaved into: Saposin-A (Protein A); Saposin-B-Val; Saposin-B (Cerebroside sulfate activator) (CSAct) (Dispersin) (Sphingolipid activator protein 1) (SAP-1) (Sulfatide/GM1 activator); Saposin-C (A1 activator) (Co-beta-glucosidase) (Glucosylceramidase activator) (Sphingolipid activator protein 2) (SAP-2); Saposin-D (Component C) (Protein C)]	

Entry	Protein names	Pathway
P07814	Bifunctional glutamate/proline--tRNA ligase (Bifunctional aminoacyl-tRNA synthetase) (Cell proliferation-inducing gene 32 protein) (Glutamatyl-prolyl-tRNA synthetase) [Includes: Glutamate--tRNA ligase (EC 6.1.1.17) (Glutamyl-tRNA synthetase) (GluRS); Proline--tRNA ligase (EC 6.1.1.15) (Prolyl-tRNA synthetase)]	
P09382	Galectin-1 (Gal-1) (14 kDa laminin-binding protein) (HLBP14) (14 kDa lectin) (Beta-galactoside-binding lectin L-14-I) (Galaptin) (HBL) (HPL) (Lactose-binding lectin 1) (Lectin galactoside-binding soluble 1) (Putative MAPK-activating protein PM12) (S-Lac lectin 1)	
P09493	Tropomyosin alpha-1 chain (Alpha-tropomyosin) (Tropomyosin-1)	
P09972	Fructose-bisphosphate aldolase C (EC 4.1.2.13) (Brain-type aldolase)	PATHWAY: Carbohydrate degradation; glycolysis; D-glyceraldehyde 3-phosphate and glycerone phosphate from D-glucose: step 4/4.
P0C221	Coiled-coil domain-containing protein 175	
P0CB38	Polyadenylate-binding protein 4-like (PABP-4-like) (Poly(A)-binding protein 4-like)	
P11831	Serum response factor (SRF)	

Entry	Protein names	Pathway
P14314	Glucosidase 2 subunit beta (80K-H protein) (Glucosidase II subunit beta) (Protein kinase C substrate 60.1 kDa protein heavy chain) (PKCSH)	PATHWAY: Glycan metabolism; N-glycan metabolism. {ECO:0000269|PubMed:10929008}.
P15531	Nucleoside diphosphate kinase A (NDK A) (NDP kinase A) (EC 2.7.4.6) (Granzyme A-activated DNase) (GAAD) (Metastasis inhibition factor nm23) (NM23-H1) (Tumor metastatic process-associated protein)	
P16109	P-selectin (CD62 antigen-like family member P) (Granule membrane protein 140) (GMP-140) (Leukocyte-endothelial cell adhesion molecule 3) (LECAM3) (Platelet activation dependent granule-external membrane protein) (PADGEM) (CD antigen CD62P)	
P18669	Phosphoglycerate mutase 1 (EC 5.4.2.11) (EC 5.4.2.4) (BPG-dependent PGAM 1) (Phosphoglycerate mutase isozyme B) (PGAM-B)	
P22314	Ubiquitin-like modifier-activating enzyme 1 (EC 6.2.1.45) (Protein A1S9) (Ubiquitin-activating enzyme E1)	PATHWAY: Protein modification; protein ubiquitination. {ECO:0000269|PubMed:1447181, ECO:0000269|PubMed:1606621}.

Entry	Protein names	Pathway
P30048	Thioredoxin-dependent peroxide reductase, mitochondrial (EC 1.11.1.24) (Antioxidant protein 1) (AOP-1) (HBC189) (Peroxiredoxin III) (Prx-III) (Peroxiredoxin-3) (Protein MER5 homolog) (Thioredoxin-dependent peroxiredoxin 3)	
P30085	UMP-CMP kinase (EC 2.7.4.14) (Deoxycytidylate kinase) (CK) (dCMP kinase) (Nucleoside-diphosphate kinase) (EC 2.7.4.6) (Uridine monophosphate/cytidine monophosphate kinase) (UMP/CMP kinase) (UMP/CMPK)	
P30101	Protein disulfide-isomerase A3 (EC 5.3.4.1) (58 kDa glucose-regulated protein) (58 kDa microsomal protein) (p58) (Disulfide isomerase ER-60) (Endoplasmic reticulum resident protein 57) (ER protein 57) (ERp57) (Endoplasmic reticulum resident protein 60) (ER protein 60) (ERp60)	
P30533	Alpha-2-macroglobulin receptor-associated protein (Alpha-2-MRAP) (Low density lipoprotein receptor-related protein-associated protein 1) (RAP)	
P30613	Pyruvate kinase PKLR (EC 2.7.1.40) (Pyruvate kinase 1) (Pyruvate kinase isozymes L/R) (R-type/L-type pyruvate kinase) (Red cell/liver pyruvate kinase)	PATHWAY: Carbohydrate degradation; glycolysis; pyruvate from D-glyceraldehyde 3-phosphate: step 5/5.
P30874	Somatostatin receptor type 2 (SS-2-R) (SS2-R) (SS2R) (SRIF-1)	

Entry	Protein names	Pathway
P30876	DNA-directed RNA polymerase II subunit RPB2 (EC 2.7.7.6) (DNA-directed RNA polymerase II 140 kDa polypeptide) (DNA-directed RNA polymerase II subunit B) (RNA polymerase II subunit 2) (RNA polymerase II subunit B2)	
P30988	Calcitonin receptor (CT-R)	
P31942	Heterogeneous nuclear ribonucleoprotein H3 (hnRNP H3) (Heterogeneous nuclear ribonucleoprotein 2H9) (hnRNP 2H9)	
P34896	Serine hydroxymethyltransferase, cytosolic (SHMT) (EC 2.1.2.1) (Glycine hydroxymethyltransferase) (Serine methylase)	PATHWAY: One-carbon metabolism; tetrahydrofolate interconversion. {ECO:0000305|PubMed:24698160}.
P35527	Keratin, type I cytoskeletal 9 (Cytokeratin-9) (CK-9) (Keratin-9) (K9)	
P35613	Basigin (5F7) (Collagenase stimulatory factor) (Extracellular matrix metalloproteinase inducer) (EMMPRIN) (Hepatoma-associated antigen) (HAb18G) (Leukocyte activation antigen M6) (OK blood group antigen) (Tumor cell-derived collagenase stimulatory factor) (TCSF) (CD antigen CD147)	
P35749	Myosin-11 (Myosin heavy chain 11) (Myosin heavy chain, smooth muscle isoform) (SMMHC)	

Entry	Protein names	Pathway
P43629	Killer cell immunoglobulin-like receptor 3DL1 (CD158 antigen-like family member E) (HLA-BW4-specific inhibitory NK cell receptor) (Natural killer-associated transcript 3) (NKAT-3) (p70 natural killer cell receptor clones CL-2/CL-11) (p70 NK receptor CL-2/CL-11) (CD antigen CD158e)	
P49321	Nuclear autoantigenic sperm protein (NASP)	
P49368	T-complex protein 1 subunit gamma (TCP-1-gamma) (CCT-gamma) (hTRiC5)	
P52306	Rap1 GTPase-GDP dissociation stimulator 1 (Exchange factor smgGDS) (SMG GDS protein) (SMG P21 stimulatory GDP/GTP exchange protein)	
P54819	Adenylate kinase 2, mitochondrial (AK 2) (EC 2.7.4.3) (ATP-AMP transphosphorylase 2) (ATP:AMP phosphotransferase) (Adenylate monophosphate kinase) [Cleaved into: Adenylate kinase 2, mitochondrial, N-terminally processed]	
P54829	Tyrosine-protein phosphatase non-receptor type 5 (EC 3.1.3.48) (Neural-specific protein-tyrosine phosphatase) (Striatum-enriched protein-tyrosine phosphatase) (STEP)	

Entry	Protein names	Pathway
P60174	Triosephosphate isomerase (TIM) (EC 5.3.1.1) (Methylglyoxal synthase) (EC 4.2.3.3) (Triose-phosphate isomerase)	PATHWAY: Carbohydrate degradation; glycolysis; D-glyceraldehyde 3-phosphate from glycerone phosphate: step 1/1. {ECO:0000255|PROSITE-ProRule:PRU10127}.; PATHWAY: Carbohydrate biosynthesis; gluconeogenesis. {ECO:0000255|PROSITE-ProRule:PRU10127}.
P61586	Transforming protein RhoA (EC 3.6.5.2) (Rho cDNA clone 12) (h12)	
P63241	Eukaryotic translation initiation factor 5A-1 (eIF-5A-1) (eIF-5A1) (Eukaryotic initiation factor 5A isoform 1) (eIF-5A) (Rev-binding factor) (eIF-4D)	
P68104	Elongation factor 1-alpha 1 (EF-1-alpha-1) (Elongation factor Tu) (EF-Tu) (Eukaryotic elongation factor 1 A-1) (eEF1A-1) (Leukocyte receptor cluster member 7)	
P84103	Serine/arginine-rich splicing factor 3 (Pre-mRNA-splicing factor SRP20) (Splicing factor, arginine/serine-rich 3)	
Q00839	Heterogeneous nuclear ribonucleoprotein U (hnRNP U) (GRIP120) (Nuclear p120 ribonucleoprotein) (Scaffold-attachment factor A) (SAF-A) (p120) (pp120)	

Entry	Protein names	Pathway
Q03001	Dystonin (230 kDa bullous pemphigoid antigen) (230/240 kDa bullous pemphigoid antigen) (Bullous pemphigoid antigen 1) (BPA) (Bullous pemphigoid antigen) (Dystonia musculorum protein) (Hemidesmosomal plaque protein)	
Q12830	Nucleosome-remodeling factor subunit BPTF (Bromodomain and PHD finger-containing transcription factor) (Fetal Alz-50 clone 1 protein) (Fetal Alzheimer antigen)	
Q13435	Splicing factor 3B subunit 2 (Pre-mRNA-splicing factor SF3b 145 kDa subunit) (SF3b145) (Spliceosome-associated protein 145) (SAP 145)	
Q13509	Tubulin beta-3 chain (Tubulin beta-4 chain) (Tubulin beta-III)	
Q14194	Dihydropyrimidinase-related protein 1 (DRP-1) (Collapsin response mediator protein 1) (CRMP-1) (Inactive dihydropyrimidinase) (Unc-33-like phosphoprotein 3) (ULIP-3)	
Q14749	Glycine N-methyltransferase (EC 2.1.1.20)	
Q14974	Importin subunit beta-1 (Importin-90) (Karyopherin subunit beta-1) (Nuclear factor p97) (Pore targeting complex 97 kDa subunit) (PTAC97)	
Q15139	Serine/threonine-protein kinase D1 (EC 2.7.11.13) (Protein kinase C mu type) (Protein kinase D) (nPKC-D1) (nPKC-mu)	

Entry	Protein names	Pathway
Q15147	1-phosphatidylinositol 4,5-bisphosphate phosphodiesterase beta-4 (EC 3.1.4.11) (Phosphoinositide phospholipase C-beta-4) (Phospholipase C-beta-4) (PLC-beta-4)	
Q15646	2'-5'-oligoadenylate synthase-like protein (2'-5'-OAS-related protein) (2'-5'-OAS-RP) (59 kDa 2'-5'-oligoadenylate synthase-like protein) (Thyroid receptor-interacting protein 14) (TR-interacting protein 14) (TRIP-14) (p59 OASL) (p59OASL)	
Q16348	Solute carrier family 15 member 2 (Kidney H(+)/peptide cotransporter) (Oligopeptide transporter, kidney isoform) (Peptide transporter 2)	
Q16595	Frataxin, mitochondrial (EC 1.16.3.1) (Friedreich ataxia protein) (Fxn) [Cleaved into: Frataxin intermediate form (i-FXN); Frataxin(56-210) (m56-FXN); Frataxin(78-210) (d-FXN) (m78-FXN); Frataxin mature form (Frataxin(81-210)) (m81-FXN)]	
Q3KQU3	MAP7 domain-containing protein 1 (Arginine/proline-rich coiled-coil domain-containing protein 1) (Proline/arginine-rich coiled-coil domain-containing protein 1)	
Q3KQV9	UDP-N-acetylhexosamine pyrophosphorylase-like protein 1 (EC 2.7.7.-)	

Entry	Protein names	Pathway
Q4G0T1	Scavenger receptor cysteine-rich domain-containing protein SCART1 (Scavenger receptor family member expressed on T cells 1)	
Q4KWH8	1-phosphatidylinositol 4,5-bisphosphate phosphodiesterase eta-1 (EC 3.1.4.11) (Phosphoinositide phospholipase C-eta-1) (Phospholipase C-eta-1) (PLC-eta-1) (Phospholipase C-like protein 3) (PLC-L3)	
Q5IJ48	Protein crumbs homolog 2 (Crumbs-like protein 2)	
Q5M7Z0	E3 ubiquitin-protein ligase RNFT1 (EC 2.3.2.27) (Protein PTD016) (RING finger and transmembrane domain-containing protein 1)	PATHWAY: Protein modification; protein ubiquitination. {ECO:0000269|PubMed:27485036}.
Q5QJE6	Deoxynucleotidyltransferase terminal-interacting protein 2 (Estrogen receptor-binding protein) (LPTS-interacting protein 2) (LPTS-RP2) (Terminal deoxynucleotidyltransferase-interacting factor 2) (TdIF2) (TdT-interacting factor 2)	
Q5T9S5	Coiled-coil domain-containing protein 18 (Sarcoma antigen NY-SAR-24)	
Q5TEZ4	Putative uncharacterized protein encoded by LINC01590 (Long intergenic non-protein coding RNA 1590)	
Q5TG30	Rho GTPase-activating protein 40 (Rho-type GTPase-activating protein 40)	
Q6PIF6	Unconventional myosin-VIIb	

Entry	Protein names	Pathway
Q6WRI0	Immunoglobulin superfamily member 10 (IgSF10) (Calvaria mechanical force protein 608) (CMF608)	
Q6XZB0	Lipase member I (LIPI) (EC 3.1.1.-) (Cancer/testis antigen 17) (CT17) (LPD lipase) (Membrane-associated phosphatidic acid-selective phospholipase A1-beta) (mPA-PLA1 beta)	
Q6Y7W6	GRB10-interacting GYF protein 2 (PERQ amino acid-rich with GYF domain-containing protein 2) (Trinucleotide repeat-containing gene 15 protein)	
Q6YHK3	CD109 antigen (150 kDa TGF-beta-1-binding protein) (C3 and PZP-like alpha-2-macroglobulin domain-containing protein 7) (Platelet-specific Gov antigen) (p180) (r150) (CD antigen CD109)	
Q6ZTA4	Tripartite motif-containing protein 67 (TRIM9-like protein)	
Q6ZV29	Patatin-like phospholipase domain-containing protein 7 (EC 3.1.1.-) (EC 3.1.1.5)	
Q7L1Q6	eIF5-mimic protein 2 (Basic leucine zipper and W2 domain-containing protein 1) (Protein Orf)	
Q7L2J0	7SK snRNA methylphosphate capping enzyme (MePCE) (EC 2.1.1.-) (Bicoid-interacting protein 3 homolog) (Bin3 homolog)	

Entry	Protein names	Pathway
Q7Z2W7	Transient receptor potential cation channel subfamily M member 8 (Long transient receptor potential channel 6) (LTrpC-6) (LTrpC6) (Transient receptor potential p8) (Trp-p8)	
Q7Z3Y9	Keratin, type I cytoskeletal 26 (Cytokeratin-26) (CK-26) (Keratin-25B) (K25B) (Keratin-26) (K26) (Type I inner root sheath-specific keratin-K25irs2)	
Q7Z5P9	Mucin-19 (MUC-19)	
Q86UB9	Transmembrane protein 135 (Peroxisomal membrane protein 52) (PMP52)	
Q86V42	Protein FAM124A	
Q8IU81	Interferon regulatory factor 2-binding protein 1 (IRF-2-binding protein 1) (IRF-2BP1) (Probable E3 ubiquitin-protein ligase IRF2BP1) (EC 2.3.2.27) (Probable RING-type E3 ubiquitin transferase IRF2BP1)	
Q8IUY3	GRAM domain-containing protein 2A	
Q8IWA4	Mitofusin-1 (EC 3.6.5.-) (Fzo homolog) (Transmembrane GTPase MFN1)	
Q8IX07	Zinc finger protein ZFPM1 (Friend of GATA protein 1) (FOG-1) (Friend of GATA 1) (Zinc finger protein 89A) (Zinc finger protein multitype 1)	
Q8IZJ3	C3 and PZP-like alpha-2-macroglobulin domain-containing protein 8	
Q8IZP7	Heparan-sulfate 6-O-sulfotransferase 3 (HS6ST-3) (EC 2.8.2.-)	

Entry	Protein names	Pathway
Q8N143	B-cell CLL/lymphoma 6 member B protein (Bcl6-associated zinc finger protein) (Zinc finger protein 62)	
Q8N5V2	Ephexin-1 (Eph-interacting exchange protein) (Neuronal guanine nucleotide exchange factor)	
Q8N8J6	Zinc finger protein 615	
Q8ND61	Uncharacterized protein C3orf20	
Q8TAQ2	SWI/SNF complex subunit SMARCC2 (BRG1-associated factor 170) (BAF170) (SWI/SNF complex 170 kDa subunit) (SWI/SNF-related matrix-associated actin-dependent regulator of chromatin subfamily C member 2)	
Q8TD57	Dynein axonemal heavy chain 3 (Axonemal beta dynein heavy chain 3) (HsADHC3) (Ciliary dynein heavy chain 3) (Dnahc3-b)	
Q8TE73	Dynein axonemal heavy chain 5 (Axonemal beta dynein heavy chain 5) (Ciliary dynein heavy chain 5)	
Q8WV93	AFG1-like ATPase (Lactation elevated protein 1) (EC 3.6.-.-) (Protein AFG1 homolog)	
Q8WWL7	G2/mitotic-specific cyclin-B3	
Q8WXE9	Stonin-2 (Stoned B)	
Q8WXF1	Paraspeckle component 1 (Paraspeckle protein 1)	

Entry	Protein names	Pathway
Q8WXX0	Dynein axonemal heavy chain 7 (Axonemal beta dynein heavy chain 7) (Ciliary dynein heavy chain 7) (Dynein heavy chain-like protein 2) (hDHC2)	
Q8WXX5	DnaJ homolog subfamily C member 9 (HDJC9) (DnaJ protein SB73)	
Q8WZ42	Titin (EC 2.7.11.1) (Connectin) (Rhabdomyosarcoma antigen MU-RMS-40.14)	
Q93097	Protein Wnt-2b (Protein Wnt-13)	
Q96CF2	Charged multivesicular body protein 4c (Chromatin-modifying protein 4c) (CHMP4c) (SNF7 homolog associated with Alix 3) (SNF7-3) (hSnf7-3) (Vacuolar protein sorting-associated protein 32-3) (Vps32-3) (hVps32-3)	
Q96ED9	Protein Hook homolog 2 (h-hook2) (hHK2)	
Q96HA1	Nuclear envelope pore membrane protein POM 121 (Nuclear envelope pore membrane protein POM 121A) (Nucleoporin Nup121) (Pore membrane protein of 121 kDa)	
Q96HP0	Dedicator of cytokinesis protein 6	
Q96MW7	Tigger transposable element-derived protein 1	
Q96P44	Collagen alpha-1(XXI) chain	
Q96RT1	Erbin (Densin-180-like protein) (Erbb2-interacting protein) (Protein LAP2)	
Q96RW7	Hemicentin-1 (Fibulin-6) (FIBL-6)	

Entry	Protein names	Pathway
Q99466	Neurogenic locus notch homolog protein 4 (Notch 4) (hNotch4) [Cleaved into: Notch 4 extracellular truncation; Notch 4 intracellular domain]	
Q99558	Mitogen-activated protein kinase kinase kinase 14 (EC 2.7.11.25) (NF-kappa-beta-inducing kinase) (HsNIK) (Serine/threonine-protein kinase NIK)	
Q99941	Cyclic AMP-dependent transcription factor ATF-6 beta (cAMP-dependent transcription factor ATF-6 beta) (Activating transcription factor 6 beta) (ATF6-beta) (Protein G13) (cAMP response element-binding protein-related protein) (Creb-rp) (cAMP-responsive element-binding protein-like 1) [Cleaved into: Processed cyclic AMP-dependent transcription factor ATF-6 beta]	
Q9BQ67	Glutamate-rich WD repeat-containing protein 1	
Q9BQE3	Tubulin alpha-1C chain (Alpha-tubulin 6) (Tubulin alpha-6 chain) [Cleaved into: Detyrosinated tubulin alpha-1C chain]	
Q9BSL1	Ubiquitin-associated domain-containing protein 1 (UBA domain-containing protein 1) (E3 ubiquitin-protein ligase subunit KPC2) (Glialblastoma cell differentiation-related protein 1) (Kip1 ubiquitination-promoting complex protein 2)	PATHWAY: Protein modification; protein ubiquitination.

Entry	Protein names	Pathway
Q9BXW6	Oxysterol-binding protein-related protein 1 (ORP-1) (OSBP-related protein 1)	
Q9H169	Stathmin-4 (Stathmin-like protein B3) (RB3)	
Q9H2Y7	Zinc finger protein 106 (Zfp-106) (Zinc finger protein 474)	
Q9H4K7	Mitochondrial ribosome-associated GTPase 2 (GTP-binding protein 5) (Protein obg homolog 1) (ObgH1)	
Q9H7E2	Tudor domain-containing protein 3	
Q9HCK8	Chromodomain-helicase-DNA-binding protein 8 (CHD-8) (EC 3.6.4.12) (ATP-dependent helicase CHD8) (Helicase with SNF2 domain 1)	
Q9NSI8	SAM domain-containing protein SAMSN-1 (Hematopoietic adaptor containing SH3 and SAM domains 1) (Nash1) (SAM domain, SH3 domain and nuclear localization signals protein 1) (SH3-SAM adaptor protein)	
Q9NUB1	Acetyl-coenzyme A synthetase 2-like, mitochondrial (EC 6.2.1.1) (Acetate--CoA ligase 2) (Acetyl-CoA synthetase 2) (AceCS2) (Acyl-CoA synthetase short-chain family member 1) (Propionate--CoA ligase) (EC 6.2.1.17)	

Entry	Protein names	Pathway
Q9NUV7	Serine palmitoyltransferase 3 (EC 2.3.1.50) (Long chain base biosynthesis protein 2b) (LCB2b) (Long chain base biosynthesis protein 3) (LCB 3) (Serine-palmitoyl-CoA transferase 3) (SPT 3)	PATHWAY: Lipid metabolism; sphingolipid metabolism. {ECO:0000269|PubMed:19416851, ECO:0000269|PubMed:19648650}.
Q9NVE7	4'-phosphopantetheine phosphatase (EC 3.1.3.-) (Inactive pantothenic acid kinase 4) (hPanK4)	
Q9NWF9	E3 ubiquitin-protein ligase RNF216 (EC 2.3.2.27) (RING finger protein 216) (RING-type E3 ubiquitin transferase RNF216) (Triad domain-containing protein 3) (Ubiquitin-conjugating enzyme 7-interacting protein 1) (Zinc finger protein inhibiting NF-kappa-B)	PATHWAY: Protein modification; protein ubiquitination.
Q9NYZ2	Mitoferrin-1 (Mitochondrial iron transporter 1) (Mitochondrial solute carrier protein) (Solute carrier family 25 member 37)	
Q9NZC7	WW domain-containing oxidoreductase (EC 1.1.1.-) (Fragile site FRA16D oxidoreductase) (Short chain dehydrogenase/reductase family 41C member 1)	
Q9P227	Rho GTPase-activating protein 23 (Rho-type GTPase-activating protein 23)	
Q9P2D1	Chromodomain-helicase-DNA-binding protein 7 (CHD-7) (EC 3.6.4.12) (ATP-dependent helicase CHD7)	

Entry	Protein names	Pathway
Q9P2P6	StAR-related lipid transfer protein 9 (START domain-containing protein 9) (StARD9)	
Q9UBW7	Zinc finger MYM-type protein 2 (Fused in myeloproliferative disorders protein) (Rearranged in atypical myeloproliferative disorder protein) (Zinc finger protein 198)	
Q9UGR2	Zinc finger CCCH domain-containing protein 7B (Rotavirus 'X'-associated non-structural protein) (RoXaN)	
Q9UJ70	N-acetyl-D-glucosamine kinase (N-acetylglucosamine kinase) (EC 2.7.1.59) (GlcNAc kinase)	PATHWAY: Amino-sugar metabolism; N-acetylneuraminate degradation. {ECO:0000269|PubMed:22692205}.
Q9UK17	Potassium voltage-gated channel subfamily D member 3 (Voltage-gated potassium channel subunit Kv4.3)	
Q9UKK3	Protein mono-ADP-ribosyltransferase PARP4 (EC 2.4.2.-) (193 kDa vault protein) (ADP-ribosyltransferase diphtheria toxin-like 4) (ARTD4) (PARP-related/IalphaI-related H5/proline-rich) (PH5P) (Poly [ADP-ribose] polymerase 4) (PARP-4) (Vault poly(ADP-ribose) polymerase) (VPARP)	
Q9UKT8	F-box/WD repeat-containing protein 2 (F-box and WD-40 domain-containing protein 2) (Protein MD6)	
Q9ULM3	YEATS domain-containing protein 2	
Q9ULP9	TBC1 domain family member 24	

Entry	Protein names	Pathway
Q9UN86	Ras GTPase-activating protein-binding protein 2 (G3BP-2) (GAP SH3 domain-binding protein 2)	
Q9UQ53	Alpha-1,3-mannosyl-glycoprotein 4-beta-N-acetylglucosaminyltransferase B (EC 2.4.1.145) (N-glycosyl-oligosaccharide-glycoprotein N-acetylglucosaminyltransferase IVb) (GlcNAc-T IVb) (GnT-IVb) (N-acetylglucosaminyltransferase IVb) (UDP-N-acetylglucosamine: alpha-1,3-D-mannoside beta-1,4-N-acetylglucosaminyltransferase IVb)	PATHWAY: Protein modification; protein glycosylation.
Q9UQC9	Calcium-activated chloride channel regulator 2 (EC 3.4.-.-) (Calcium-activated chloride channel family member 2) (hCLCA2) (Calcium-activated chloride channel protein 3) (CaCC-3) (hCaCC-3) [Cleaved into: Calcium-activated chloride channel regulator 2, 109 kDa form; Calcium-activated chloride channel regulator 2, 35 kDa form]	
Q9Y235	C->U-editing enzyme APOBEC-2 (EC 3.5.4.36) (mRNA(cytosine(6666)) deaminase 2)	
Q9Y283	Inversin (Inversion of embryo turning homolog) (Nephrocystin-2)	

Entry	Protein names	Pathway
Q9Y3M8	StAR-related lipid transfer protein 13 (46H23.2) (Deleted in liver cancer 2 protein) (DLC-2) (Rho GTPase-activating protein) (START domain-containing protein 13) (StARD13)	
Q9Y4B5	Microtubule cross-linking factor 1 (Coiled-coil domain-containing protein 165) (PAR-1-interacting protein) (SOGA family member 2)	
Q9Y4U1	Cyanocobalamin reductase / alkylcobalamin dealkylase (Alkylcobalamin:glutathione S-alkyltransferase) (EC 2.5.1.151) (CblC) (Cyanocobalamin reductase (cyanide-eliminating)) (EC 1.16.1.6) (Methylmalonic aciduria and homocystinuria type C protein) (MMACHC)	
Q9Y615	Actin-like protein 7A (Actin-like-7-alpha)	
A5D8V6	Vacuolar protein sorting-associated protein 37C (hVps37C) (ESCRT-I complex subunit VPS37C)	
A5PLK6	Regulator of G-protein signaling protein-like	
A6ND36	Protein FAM83G (Protein associated with SMAD1)	
A6NGB9	WAS/WASL-interacting protein family member 3 (Corticosteroids and regional expression protein 16 homolog)	
A6PVS8	Leucine-rich repeat and IQ domain-containing protein 3 (Leucine-rich repeat-containing protein 44)	
F5H4A9	Uncharacterized membrane protein C3orf80	

Entry	Protein names	Pathway
O14744	Protein arginine N-methyltransferase 5 (PRMT5) (EC 2.1.1.320) (72 kDa ICln-binding protein) (Histone-arginine N-methyltransferase PRMT5) (Jak-binding protein 1) (Shk1 kinase-binding protein 1 homolog) (SKB1 homolog) (SKB1Hs) [Cleaved into: Protein arginine N-methyltransferase 5, N-terminally processed]	
O14757	Serine/threonine-protein kinase Chk1 (EC 2.7.11.1) (CHK1 checkpoint homolog) (Cell cycle checkpoint kinase) (Checkpoint kinase-1)	
O14893	Gem-associated protein 2 (Gemin-2) (Component of gems 2) (Survival of motor neuron protein-interacting protein 1) (SMN-interacting protein 1)	
O15547	P2X purinoceptor 6 (P2X6) (ATP receptor) (P2XM) (Purinergic receptor) (Purinergic receptor P2X-like 1)	
O43824	Putative GTP-binding protein 6 (Pseudoautosomal GTP-binding protein-like)	
O43852	Calumenin (Crocalbin) (IEF SSP 9302)	
O60306	RNA helicase aquarius (EC 3.6.4.13) (Intron-binding protein of 160 kDa) (IBP160)	
O75153	Clustered mitochondria protein homolog	
O75487	Glypican-4 (K-glypican) [Cleaved into: Secreted glypican-4]	

Entry	Protein names	Pathway
O75531	Barrier-to-autointegration factor (Breakpoint cluster region protein 1) [Cleaved into: Barrier-to-autointegration factor, N-terminally processed]	
O75947	ATP synthase subunit d, mitochondrial (ATPase subunit d) (ATP synthase peripheral stalk subunit d)	
O76038	Secretagogin	
O95755	Ras-related protein Rab-36	
O95785	Protein Wiz (Widely-interspaced zinc finger-containing protein) (Zinc finger protein 803)	
P00441	Superoxide dismutase [Cu-Zn] (EC 1.15.1.1) (Superoxide dismutase 1) (hSod1)	
P01042	Kininogen-1 (Alpha-2-thiol proteinase inhibitor) (Fitzgerald factor) (High molecular weight kininogen) (HMWK) (Williams-Fitzgerald-Flaujeac factor) [Cleaved into: Kininogen-1 heavy chain; T-kinin (Ile-Ser-Bradykinin); Bradykinin (Kallidin I); Lysyl-bradykinin (Kallidin II); Kininogen-1 light chain; Low molecular weight growth-promoting factor]	
P01699	Immunoglobulin lambda variable 1-44 (Ig lambda chain V-I region MEM) (Ig lambda chain V-I region VOR)	

Entry	Protein names	Pathway
P02647	Apolipoprotein A-I (Apo-AI) (ApoA-I) (Apolipoprotein A1) [Cleaved into: Proapolipoprotein A-I (ProapoA-I); Truncated apolipoprotein A-I (Apolipoprotein A-I(1-242))]	
P04632	Calpain small subunit 1 (CSS1) (Calcium-activated neutral proteinase small subunit) (CANP small subunit) (Calcium-dependent protease small subunit) (CDPS) (Calcium-dependent protease small subunit 1) (Calpain regulatory subunit)	
P05386	60S acidic ribosomal protein P1 (Large ribosomal subunit protein P1)	
P05387	60S acidic ribosomal protein P2 (Large ribosomal subunit protein P2) (Renal carcinoma antigen NY-REN-44)	
P09429	High mobility group protein B1 (High mobility group protein 1) (HMG-1)	
P10253	Lysosomal alpha-glucosidase (EC 3.2.1.20) (Acid maltase) (Aglucosidase alfa) [Cleaved into: 76 kDa lysosomal alpha-glucosidase; 70 kDa lysosomal alpha-glucosidase]	
P10599	Thioredoxin (Trx) (ATL-derived factor) (ADF) (Surface-associated sulphydryl protein) (SASP) (allergen Hom s Trx)	

Entry	Protein names	Pathway
P10909	Clusterin (Aging-associated gene 4 protein) (Apolipoprotein J) (Apo-J) (Complement cytolysis inhibitor) (CLI) (Complement-associated protein SP-40,40) (Ku70-binding protein 1) (NA1/NA2) (Sulfated glycoprotein 2) (SGP-2) (Testosterone-repressed prostate message 2) (TRPM-2) [Cleaved into: Clusterin beta chain (ApoJalpha) (Complement cytolysis inhibitor a chain); Clusterin alpha chain (ApoJbeta) (Complement cytolysis inhibitor b chain)]	
P11388	DNA topoisomerase 2-alpha (EC 5.6.2.2) (DNA topoisomerase II, alpha isozyme)	
P12035	Keratin, type II cytoskeletal 3 (65 kDa cytokeratin) (Cytokeratin-3) (CK-3) (Keratin-3) (K3) (Type-II keratin Kb3)	
P13747	HLA class I histocompatibility antigen, alpha chain E (MHC class I antigen E) [Cleaved into: Soluble HLA class I histocompatibility antigen, alpha chain E (sHLA-E)]	
P14373	Zinc finger protein RFP (EC 2.3.2.27) (RING finger protein 76) (RING-type E3 ubiquitin transferase TRIM27) (Ret finger protein) (Tripartite motif-containing protein 27)	

Entry	Protein names	Pathway
P15144	Aminopeptidase N (AP-N) (hAPN) (EC 3.4.11.2) (Alanyl aminopeptidase) (Aminopeptidase M) (AP-M) (Microsomal aminopeptidase) (Myeloid plasma membrane glycoprotein CD13) (gp150) (CD antigen CD13)	
P16401	Histone H1.5 (Histone H1a) (Histone H1b) (Histone H1s-3)	
P17066	Heat shock 70 kDa protein 6 (Heat shock 70 kDa protein B')	
P17181	Interferon alpha/beta receptor 1 (IFN-R-1) (IFN-alpha/beta receptor 1) (Cytokine receptor class-II member 1) (Cytokine receptor family 2 member 1) (CRF2-1) (Type I interferon receptor 1)	
P17643	5,6-dihydroxyindole-2-carboxylic acid oxidase (DHICA oxidase) (EC 1.14.18.-) (Catalase B) (Glycoprotein 75) (Melanoma antigen gp75) (Tyrosinase-related protein 1) (TRP) (TRP-1) (TRP1)	PATHWAY: Pigment biosynthesis; melanin biosynthesis. {ECO:0000269|PubMed:16704458, ECO:0000269|PubMed:22556244, ECO:0000269|PubMed:23504663}.
P19838	Nuclear factor NF-kappa-B p105 subunit (DNA-binding factor KBF1) (EBP-1) (Nuclear factor of kappa light polypeptide gene enhancer in B-cells 1) [Cleaved into: Nuclear factor NF-kappa-B p50 subunit]	

Entry	Protein names	Pathway
P20809	Interleukin-11 (IL-11) (Adipogenesis inhibitory factor) (AGIF) (Oprelvekin)	
P23109	AMP deaminase 1 (EC 3.5.4.6) (AMP deaminase isoform M) (Myoadenylate deaminase)	PATHWAY: Purine metabolism; IMP biosynthesis via salvage pathway; IMP from AMP: step 1/1.
P25398	40S ribosomal protein S12 (Small ribosomal subunit protein eS12)	
P26583	High mobility group protein B2 (High mobility group protein 2) (HMG-2)	
P30405	Peptidyl-prolyl cis-trans isomerase F, mitochondrial (PPIase F) (EC 5.2.1.8) (Cyclophilin D) (CyP-D) (CypD) (Cyclophilin F) (Mitochondrial cyclophilin) (CyP-M) (Rotamase F)	
P31948	Stress-induced-phosphoprotein 1 (STI1) (Hsc70/Hsp90-organizing protein) (Hop) (Renal carcinoma antigen NY-REN-11) (Transformation-sensitive protein IEF SSP 3521)	
P34931	Heat shock 70 kDa protein 1-like (Heat shock 70 kDa protein 1L) (Heat shock 70 kDa protein 1-Hom) (HSP70-Hom)	
P35573	Glycogen debranching enzyme (Glycogen debrancher) [Includes: 4-alpha-glucanotransferase (EC 2.4.1.25) (Oligo-1,4-1,4-glucantransferase); Amylo-alpha-1,6-glucosidase (Amylo-1,6-glucosidase) (EC 3.2.1.33) (Dextrin 6-alpha-D-glucosidase)]	

Entry	Protein names	Pathway
P35580	Myosin-10 (Cellular myosin heavy chain, type B) (Myosin heavy chain 10) (Myosin heavy chain, non-muscle IIb) (Non-muscle myosin heavy chain B) (NMMHC-B) (Non-muscle myosin heavy chain IIb) (NMMHC II-b) (NMMHC-IIB)	
P38646	Stress-70 protein, mitochondrial (75 kDa glucose-regulated protein) (GRP-75) (Heat shock 70 kDa protein 9) (Mortalin) (MOT) (Peptide-binding protein 74) (PBP74)	
P40121	Macrophage-capping protein (Actin regulatory protein CAP-G)	
P48741	Putative heat shock 70 kDa protein 7 (Heat shock 70 kDa protein B)	
P56524	Histone deacetylase 4 (HD4) (EC 3.5.1.98)	
P62879	Guanine nucleotide-binding protein G(I)/G(S)/G(T) subunit beta-2 (G protein subunit beta-2) (Transducin beta chain 2)	
P83916	Chromobox protein homolog 1 (HP1Hsbeta) (Heterochromatin protein 1 homolog beta) (HP1 beta) (Heterochromatin protein p25) (M31) (Modifier 1 protein) (p25beta)	
P84074	Neuron-specific calcium-binding protein hippocalcin (Calcium-binding protein BDR-2)	
P98187	Cytochrome P450 4F8 (EC 1.14.14.1) (CYPIVF8)	PATHWAY: Lipid metabolism; fatty acid metabolism. {ECO:0000269|PubMed:10791960}.

Entry	Protein names	Pathway
Q00341	Vigilin (High density lipoprotein-binding protein) (HDL-binding protein)	
Q01130	Serine/arginine-rich splicing factor 2 (Protein PR264) (Splicing component, 35 kDa) (Splicing factor SC35) (SC-35) (Splicing factor, arginine/serine-rich 2)	
Q07021	Complement component 1 Q subcomponent-binding protein, mitochondrial (ASF/SF2-associated protein p32) (Glycoprotein gC1qBP) (C1qBP) (Hyaluronan-binding protein 1) (Mitochondrial matrix protein p32) (gC1q-R protein) (p33) (SF2AP32)	
Q07890	Son of sevenless homolog 2 (SOS-2)	
Q12860	Contactin-1 (Glycoprotein gp135) (Neural cell surface protein F3)	
Q13561	Dynactin subunit 2 (50 kDa dynein-associated polypeptide) (Dynactin complex 50 kDa subunit) (DCTN-50) (p50 dynamitin)	
Q13813	Spectrin alpha chain, non-erythrocytic 1 (Alpha-II spectrin) (Fodrin alpha chain) (Spectrin, non-erythroid alpha subunit)	
Q14126	Desmoglein-2 (Cadherin family member 5) (HDGC)	
Q14554	Protein disulfide-isomerase A5 (EC 5.3.4.1) (Protein disulfide isomerase-related protein)	
Q14863	POU domain, class 6, transcription factor 1 (Brain-specific homeobox/POU domain protein 5) (Brain-5) (Brn-5) (mPOU homeobox protein)	

Entry	Protein names	Pathway
Q15034	Probable E3 ubiquitin-protein ligase HERC3 (EC 2.3.2.26) (HECT domain and RCC1-like domain-containing protein 3) (HECT-type E3 ubiquitin transferase HERC3)	PATHWAY: Protein modification; protein ubiquitination.
Q15365	Poly(rC)-binding protein 1 (Alpha-CP1) (Heterogeneous nuclear ribonucleoprotein E1) (hnRNP E1) (Nucleic acid-binding protein SUB2.3)	
Q15746	Myosin light chain kinase, smooth muscle (MLCK) (smMLCK) (EC 2.7.11.18) (Kinase-related protein) (KRP) (Telokin) [Cleaved into: Myosin light chain kinase, smooth muscle, deglutamylated form]	
Q15916	Zinc finger and BTB domain-containing protein 6 (Zinc finger protein 482) (Zinc finger protein with interaction domain)	
Q16890	Tumor protein D53 (hD53) (Tumor protein D52-like 1)	
Q1XH10	SKI/DACH domain-containing protein 1 (Protein DLN-1)	
Q2TAC2	Coiled-coil domain-containing protein 57	
Q2WEN9	Carcinoembryonic antigen-related cell adhesion molecule 16 (Carcinoembryonic antigen-like 2)	

Entry	Protein names	Pathway
Q4LDE5	Sushi, von Willebrand factor type A, EGF and pentraxin domain-containing protein 1 (CCP module-containing protein 22) (Polydom) (Selectin-like osteoblast-derived protein) (SEL-OB) (Serologically defined breast cancer antigen NY-BR-38)	
Q5JU67	Cilia- and flagella-associated protein 157	
Q5JWR5	Protein dopey-1	
Q5K4L6	Solute carrier family 27 member 3 (EC 6.2.1.-) (Arachidonate--CoA ligase) (EC 6.2.1.15) (Long-chain fatty acid transport protein 3) (FATP-3) (Fatty acid transport protein 3) (Very long-chain acyl-CoA synthetase homolog 3) (VLCS-3)	
Q5NDL2	EGF domain-specific O-linked N-acetylglucosamine transferase (EC 2.4.1.255) (Extracellular O-linked N-acetylglucosamine transferase)	
Q5SQ80	Putative ankyrin repeat domain-containing protein 20A2 (Ankyrin repeat domain-containing protein 20A2 pseudogene)	
Q5VST9	Obscurin (EC 2.7.11.1) (Obscurin-RhoGEF) (Obscurin-myosin light chain kinase) (Obscurin-MLCK)	
Q5VTJ3	Kelch domain-containing protein 7A	
Q6BDS2	UHRF1-binding protein 1 (ICBP90-binding protein 1) (Ubiquitin-like containing PHD and RING finger domains 1-binding protein 1)	
Q6IQ22	Ras-related protein Rab-12	

Entry	Protein names	Pathway
Q6R2W3	SCAN domain-containing protein 3 (Transposon-derived Buster4 transposase-like protein) (Zinc finger BED domain-containing protein 9)	
Q70IA8	MOB kinase activator 3C (Mob1 homolog 2C) (Mps one binder kinase activator-like 2C)	
Q7KZI7	Serine/threonine-protein kinase MARK2 (EC 2.7.11.1) (EC 2.7.11.26) (ELKL motif kinase 1) (EMK-1) (MAP/microtubule affinity-regulating kinase 2) (PAR1 homolog) (PAR1 homolog b) (Par-1b) (Par1b)	
Q7Z333	Probable helicase senataxin (EC 3.6.4.-) (Amyotrophic lateral sclerosis 4 protein) (SEN1 homolog) (Senataxin)	
Q86SQ4	Adhesion G-protein coupled receptor G6 (Developmentally regulated G-protein-coupled receptor) (G-protein coupled receptor 126) (Vascular inducible G protein-coupled receptor) [Cleaved into: ADGRG6 N-terminal fragment (ADGRG6-NTF); ADGRG6 C-terminal fragment (ADGRG6-CTF)]	
Q86V21	Acetoacetyl-CoA synthetase (EC 6.2.1.16) (Acyl-CoA synthetase family member 1) (Protein sur-5 homolog)	

Entry	Protein names	Pathway
Q86V81	THO complex subunit 4 (Tho4) (Ally of AML-1 and LEF-1) (Aly/REF export factor) (Transcriptional coactivator Aly/REF) (bZIP-enhancing factor BEF)	
Q86VH2	Kinesin-like protein KIF27	
Q8IZJ6	Inactive L-threonine 3-dehydrogenase, mitochondrial (Short chain dehydrogenase/reductase family 14E member 1 pseudogene)	
Q8N3F8	MICAL-like protein 1 (Molecule interacting with Rab13) (MIRab13)	
Q8NET6	Carbohydrate sulfotransferase 13 (EC 2.8.2.5) (Chondroitin 4-O-sulfotransferase 3) (Chondroitin 4-sulfotransferase 3) (C4ST-3) (C4ST3)	
Q8NF91	Nesprin-1 (Enaptin) (KASH domain-containing protein 1) (KASH1) (Myocyte nuclear envelope protein 1) (Myne-1) (Nuclear envelope spectrin repeat protein 1) (Synaptic nuclear envelope protein 1) (Syne-1)	
Q8TA86	Retinitis pigmentosa 9 protein (Pim-1-associated protein) (PAP-1)	
Q8TBY9	Cilia- and flagella-associated protein 251 (WD repeat-containing protein 66)	

Entry	Protein names	Pathway
Q8TC36	SUN domain-containing protein 5 (Sad1 and UNC84 domain-containing protein 5) (Sperm-associated antigen 4-like protein) (Testis and spermatogenesis-related gene 4 protein)	
Q8TC59	Piwi-like protein 2 (EC 3.1.26.-) (Cancer/testis antigen 80) (CT80)	
Q8WTR7	Zinc finger protein 473 (Zinc finger protein 100 homolog) (Zfp-100)	
Q92508	Piezo-type mechanosensitive ion channel component 1 (Membrane protein induced by beta-amyloid treatment) (Mib) (Protein FAM38A)	
Q92844	TRAF family member-associated NF-kappa-B activator (TRAF-interacting protein) (I-TRAF)	
Q96BA8	Cyclic AMP-responsive element-binding protein 3-like protein 1 (cAMP-responsive element-binding protein 3-like protein 1) (Old astrocyte specifically-induced substance) (OASIS) [Cleaved into: Processed cyclic AMP-responsive element-binding protein 3-like protein 1]	
Q96GT9	X antigen family member 2 (XAGE-2) (Cancer/testis antigen 12.2) (CT12.2) (G antigen family D member 3)	
Q96JG6	Syndetin (Coiled-coil domain-containing protein 132) (EARP/GARPII complex subunit VPS50)	

Entry	Protein names	Pathway
Q96M11	Hydrolethalus syndrome protein 1	
Q96MI9	Cytosolic carboxypeptidase 4 (EC 3.4.17.-) (EC 3.4.17.24) (ATP/GTP-binding protein-like 1) (Protein deglutamylase CCP4)	
Q96MR6	Cilia- and flagella-associated protein 57 (WD repeat-containing protein 65)	
Q96MY7	Protein FAM161B	
Q96PE2	Rho guanine nucleotide exchange factor 17 (164 kDa Rho-specific guanine-nucleotide exchange factor) (p164-RhoGEF) (p164RhoGEF) (Tumor endothelial marker 4)	
Q96PX1	E3 ubiquitin ligase RNF157 (EC 2.3.2.27) (RING finger protein 157) (RING-type E3 ubiquitin transferase RNF157)	
Q96Q91	Anion exchange protein 4 (AE 4) (Anion exchanger 4) (Sodium bicarbonate cotransporter 5) (Solute carrier family 4 member 9)	
Q9BRL6	Serine/arginine-rich splicing factor 8 (Pre-mRNA-splicing factor SRP46) (Splicing factor SRp46) (Splicing factor, arginine/serine-rich 2B)	
Q9BRS8	La-related protein 6 (Acheron) (Achn) (La ribonucleoprotein domain family member 6)	

Entry	Protein names	Pathway
Q9BRZ2	E3 ubiquitin-protein ligase TRIM56 (EC 2.3.2.27) (RING finger protein 109) (Tripartite motif-containing protein 56)	PATHWAY: Protein modification; protein ubiquitination. {ECO:0000269|PubMed:21289118, ECO:0000269|PubMed:29426904}.
Q9BT09	Protein canopy homolog 3 (CTG repeat protein 4a) (Expanded repeat-domain protein CAG/CTG 5) (Protein associated with TLR4) (Trinucleotide repeat-containing gene 5 protein)	
Q9BXS5	AP-1 complex subunit mu-1 (AP-mu chain family member mu1A) (Adaptor protein complex AP-1 subunit mu-1) (Adaptor-related protein complex 1 subunit mu-1) (Clathrin assembly protein complex 1 mu-1 medium chain 1) (Clathrin coat assembly protein AP47) (Clathrin coat-associated protein AP47) (Golgi adaptor HA1/AP1 adaptin mu-1 subunit) (Mu-adaptin 1) (Mu1A-adaptin)	
Q9BY60	Gamma-aminobutyric acid receptor-associated protein-like 3 (GABA(A) receptor-associated protein-like 3)	
Q9BZ29	Dedicator of cytokinesis protein 9 (Cdc42 guanine nucleotide exchange factor zizimin-1) (Zizimin-1)	

Entry	Protein names	Pathway
Q9C0I1	Myotubularin-related protein 12 (Inactive phosphatidylinositol 3-phosphatase 12) (Phosphatidylinositol 3 phosphate 3-phosphatase adapter subunit) (3-PAP) (3-phosphatase adapter protein)	
Q9H3T3	Semaphorin-6B (Semaphorin-Z) (Sema Z)	
Q9H920	RING finger protein 121	
Q9HCJ5	Zinc finger SWIM domain-containing protein 6	
Q9NRD9	Dual oxidase 1 (EC 1.11.1.-) (EC 1.6.3.1) (Large NOX 1) (Long NOX 1) (NADPH thyroid oxidase 1) (Thyroid oxidase 1)	PATHWAY: Hormone biosynthesis; thyroid hormone biosynthesis.
Q9NRX4	14 kDa phosphohistidine phosphatase (EC 3.9.1.3) (Phosphohistidine phosphatase 1) (PHPT1) (Protein histidine phosphatase) (PHP) (Protein janus-A homolog)	
Q9NUD5	Zinc finger CCHC domain-containing protein 3	
Q9NVR2	Integrator complex subunit 10 (Int10)	
Q9NVX2	Notchless protein homolog 1	
Q9NYQ6	Cadherin EGF LAG seven-pass G-type receptor 1 (Cadherin family member 9) (Flamingo homolog 2) (hFmi2)	
Q9NZR2	Low-density lipoprotein receptor-related protein 1B (LRP-1B) (Low-density lipoprotein receptor-related protein-deleted in tumor) (LRP-DIT)	

Entry	Protein names	Pathway
Q9P0L0	Vesicle-associated membrane protein-associated protein A (VAMP-A) (VAMP-associated protein A) (VAP-A) (33 kDa VAMP-associated protein) (VAP-33)	
Q9P281	BAH and coiled-coil domain-containing protein 1 (Bromo adjacent homology domain-containing protein 2) (BAH domain-containing protein 2)	
Q9P2E9	Ribosome-binding protein 1 (180 kDa ribosome receptor homolog) (RRp) (ES/130-related protein) (Ribosome receptor protein)	
Q9UBV7	Beta-1,4-galactosyltransferase 7 (Beta-1,4-GalTase 7) (Beta4Gal-T7) (b4Gal-T7) (EC 2.4.1.-) (Proteoglycan UDP-galactose:beta-xylose beta1,4-galactosyltransferase I) (UDP-Gal:beta-GlcNAc beta-1,4-galactosyltransferase 7) (UDP-galactose:beta-N-acetylglucosamine beta-1,4-galactosyltransferase 7) (UDP-galactose:beta-xylose beta-1,4-galactosyltransferase) (XGPT) (XGalT-1) (Xylosylprotein 4-beta-galactosyltransferase) (EC 2.4.1.133) (Xylosylprotein beta-1,4-galactosyltransferase)	PATHWAY: Protein modification; protein glycosylation.
Q9UG01	Intraflagellar transport protein 172 homolog	

Entry	Protein names	Pathway
Q9UGK8	Secretion-regulating guanine nucleotide exchange factor (Deafness locus-associated putative guanine nucleotide exchange factor) (DelGEF) (Guanine nucleotide exchange factor-related protein)	
Q9UHD1	Cysteine and histidine-rich domain-containing protein 1 (CHORD domain-containing protein 1) (CHORD-containing protein 1) (CHP-1) (Protein morgana)	
Q9UJT1	Tubulin delta chain (Delta-tubulin)	
Q9UJU6	Drebrin-like protein (Cervical SH3P7) (Cervical mucin-associated protein) (Drebrin-F) (HPK1-interacting protein of 55 kDa) (HIP-55) (SH3 domain-containing protein 7)	
Q9ULM2	Zinc finger protein 490	
Q9UMN6	Histone-lysine N-methyltransferase 2B (Lysine N-methyltransferase 2B) (EC 2.1.1.364) (Myeloid/lymphoid or mixed-lineage leukemia protein 4) (Trithorax homolog 2) (WW domain-binding protein 7) (WBP-7)	
Q9UNP9	Peptidyl-prolyl cis-trans isomerase E (PPIase E) (EC 5.2.1.8) (Cyclophilin E) (Cyclophilin-33) (Rotamase E)	
Q9UPS6	Histone-lysine N-methyltransferase SETD1B (EC 2.1.1.364) (Lysine N-methyltransferase 2G) (SET domain-containing protein 1B) (hSET1B)	
Q9Y490	Talin-1	

Entry	Protein names	Pathway
Q9Y5Z4	Heme-binding protein 2 (Placental protein 23) (PP23) (Protein SOUL)	
Q9Y6N8	Cadherin-10 (T2-cadherin)	

Table S3: Total unique proteins and their associated Uniprot pathways for 100 mM H_2O_2 that were modified by FPOP

Layer	Master Protein Accession
Outer	A1KZ92
Outer	A2A2Z9
Outer	A6NGB9
Outer	O00458
Outer	O14745
Outer	O43169
Outer	O43324
Outer	O43399
Outer	O43707
Outer	O43852
Outer	O60506
Outer	O60664
Outer	O75153
Outer	P00352
Outer	P00441

Layer	Master Protein Accession
Outer	P00558
Outer	P02533
Outer	P02545
Outer	P04350
Outer	P05387
Outer	P05783
Outer	P06733
Outer	P07108
Outer	P07437
Outer	P07900
Outer	P08238
Outer	P08727
Outer	P0DMV9
Outer	P10809
Outer	P11142
Outer	P12111
Outer	P13073
Outer	P13598
Outer	P13645
Outer	P14625
Outer	P16401
Outer	P16989
Outer	P17677
Outer	P18859
Outer	P20929
Outer	P22626
Outer	P27797
Outer	P29692
Outer	P30101

Layer	Master Protein Accession
Outer	P30405
Outer	P30459
Outer	P31948
Outer	P38646
Outer	P41229
Outer	P50402
Outer	P50995
Outer	P51858
Outer	P52815
Outer	P54727
Outer	P54819
Outer	P61978
Outer	P62805
Outer	P62841
Outer	P68371
Outer	Q01844
Outer	Q02952
Outer	Q09666
Outer	Q11128
Outer	Q13838
Outer	Q13885
Outer	Q14103
Outer	Q14847
Outer	Q15111
Outer	Q15185
Outer	Q3ZCM7
Outer	Q53H47
Outer	Q5D1E8
Outer	Q5JU67

Layer	Master Protein Accession
Outer	Q5JUR7
Outer	Q76B58
Outer	Q7Z7H5
Outer	Q8N100
Outer	Q8N8V2
Outer	Q8NFI4
Outer	Q8TEQ0
Outer	Q8WXI7
Outer	Q8WXX5
Outer	Q92766
Outer	Q92993
Outer	Q96HE7
Outer	Q96JB2
Outer	Q99497
Outer	Q9BUF5
Outer	Q9BVK6
Outer	Q9BWQ6
Outer	Q9BYX2
Outer	Q9H892
Outer	Q9HCN8
Outer	Q9NQC3
Outer	Q9NS69
Outer	Q9UFH2
Outer	Q9UNP9
Outer	Q9Y3X0
Outer	Q9Y4B5
Outer	Q9Y5S9
Outer	Q9Y6I3
Outer	O14950

Layer	Master Protein Accession
Outer	O15381
Outer	O60814
Outer	O75531
Outer	O75582
Outer	P00338
Outer	P00403
Outer	P04075
Outer	P04406
Outer	P04632
Outer	P05787
Outer	P06576
Outer	P06744
Outer	P06748
Outer	P07237
Outer	P07305
Outer	P08708
Outer	P08779
Outer	P09429
Outer	P0CG39
Outer	P11021
Outer	P11166
Outer	P12109
Outer	P13639
Outer	P13667
Outer	P13804
Outer	P14314
Outer	P15311
Outer	P15531
Outer	P16070

Layer	Master Protein Accession
Outer	P17066
Outer	P18669
Outer	P21817
Outer	P23528
Outer	P25705
Outer	P26583
Outer	P27348
Outer	P30040
Outer	P30041
Outer	P31942
Outer	P31946
Outer	P31947
Outer	P31949
Outer	P34932
Outer	P35579
Outer	P35613
Outer	P37802
Outer	P40926
Outer	P42126
Outer	P60174
Outer	P60709
Outer	P61981
Outer	P62258
Outer	P62306
Outer	P62847
Outer	P63104
Outer	P63208
Outer	P67809
Outer	Q03924

Layer	Master Protein Accession
Outer	Q04917
Outer	Q06830
Outer	Q07955
Outer	Q13123
Outer	Q13285
Outer	Q13509
Outer	Q14697
Outer	Q15149
Outer	Q16629
Outer	Q562R1
Outer	Q5TH69
Outer	Q8WXD9
Outer	Q92597
Outer	Q96T17
Outer	Q99525
Outer	Q9C037
Outer	Q9H4B7
Outer	Q9H5V8
Outer	Q9H799
Outer	Q9NP08
Outer	Q9NUJ1
Outer	Q9NZ71
Outer	Q9P0K7
Outer	Q9P0L0
Outer	Q9UQ80
Outer	Q9Y281
Inner	A0A096LP55
Inner	A2RUQ5
Inner	A8MWX3

Layer	Master Protein Accession
Inner	O15078
Inner	O43865
Inner	O60287
Inner	O60765
Inner	O75150
Inner	O75165
Inner	P00738
Inner	P07478
Inner	P08574
Inner	P10809
Inner	P11532
Inner	P16104
Inner	P16234
Inner	P17066
Inner	P17568
Inner	P17677
Inner	P21333
Inner	P38646
Inner	P43007
Inner	P48736
Inner	P55011
Inner	P68032
Inner	P78524
Inner	P83436
Inner	Q01546
Inner	Q13506
Inner	Q13885
Inner	Q14050
Inner	Q14204

Layer	Master Protein Accession
Inner	Q14839
Inner	Q5T0D9
Inner	Q6WRI0
Inner	Q6ZMI0
Inner	Q6ZVD7
Inner	Q7L5N7
Inner	Q7Z589
Inner	Q86U44
Inner	Q8IWA4
Inner	Q8N143
Inner	Q8N1F8
Inner	Q8N5H7
Inner	Q8NC74
Inner	Q8NCM8
Inner	Q8NDH2
Inner	Q8NF91
Inner	Q8TD26
Inner	Q8WW12
Inner	Q92614
Inner	Q92766
Inner	Q96DN5
Inner	Q96JB1
Inner	Q96QE3
Inner	Q99576
Inner	Q9H892
Inner	Q9HCL3
Inner	Q9NQS7
Inner	Q9NVD7
Inner	Q9NXE4

Layer	Master Protein Accession
Inner	Q9NXS2
Inner	Q9P2R6
Inner	Q9UBG3
Inner	Q9UFH2
Inner	Q9ULB4
Inner	Q9ULH0
Inner	Q9UP38
Inner	Q9Y253
Inner	Q9Y4L1
Inner	P14314
Inner	Q13509
Inner	P50502
Inner	P00558
Inner	P14625
Inner	P51991
Inner	P27816
Inner	P0DMV9
Inner	Q9H910
Inner	P11021
Inner	P30041
Inner	Q02952
Inner	P02545
Inner	P51572
Inner	P00403
Inner	O75947
Inner	P11142
Inner	P52272
Inner	P43243
Inner	Q13435

Layer	Master Protein Accession
Inner	P54819
Inner	P07900
Inner	P13667
Inner	Q99497
Inner	P33176
Inner	Q92597
Inner	Q92688
Inner	Q15365
Inner	P20700
Inner	E9PAV3
Inner	O60506
Inner	P30405
Inner	Q14847
Inner	P06576
Inner	P08962
Inner	P54727
Inner	P05534
Inner	P62258
Inner	Q96HE7
Inner	P23497
Inner	P0DP25
Inner	P08727
Inner	P07437
Inner	P07237
Inner	Q13263
Inner	Q6FI13
Inner	O43707
Inner	P00441
Inner	Q9NQC3

Layer	Master Protein Accession
Inner	P04350
Inner	P08708
Inner	P37802
Inner	P04406
Inner	P06733
Inner	Q9Y224
Inner	Q00059
Inner	O00193
Inner	Q15366
Inner	O60814
Inner	O95202
Inner	P22626
Inner	P52815
Inner	O43399
Inner	P15311
Inner	P35579
Inner	P13073
Inner	Q8NC51
Inner	P12277
Inner	P09455
Inner	P20674
Inner	P05787
Inner	P09429
Inner	P25705
Inner	P08238
Inner	P62937
Inner	P30101
Inner	O43852
Inner	P62805

Layer	Master Protein Accession
Inner	P60709
Inner	Q07021
Inner	P35613
Inner	Q06830
Inner	P18669
Inner	P18859
Inner	O60664
Inner	P07305
Inner	P08865
Inner	Q14103
Inner	Q9Y3U8
Inner	P26583
Inner	P04075
Inner	P30044
Inner	P05386
Inner	P62841
Inner	Q9BUF5
Inner	P30443
Inner	P08779
Inner	P06899
Inner	Q29960
Inner	P68371
Inner	P10319
Inner	P16188
Inner	P10316
Inner	A0A0J9YXQ4
Inner	A6NHL2
Inner	O14737
Inner	O14950

Layer	Master Protein Accession
Inner	O15061
Inner	O15511
Inner	O60888
Inner	O75093
Inner	O75369
Inner	O75400
Inner	O75475
Inner	O75969
Inner	O95169
Inner	P00338
Inner	P01042
Inner	P04264
Inner	P04632
Inner	P04792
Inner	P05783
Inner	P06744
Inner	P06748
Inner	P07108
Inner	P07195
Inner	P07355
Inner	P09172
Inner	P09211
Inner	P09972
Inner	P11055
Inner	P11166
Inner	P11177
Inner	P12270
Inner	P12532
Inner	P12814

Layer	Master Protein Accession
Inner	P13674
Inner	P13693
Inner	P13804
Inner	P13929
Inner	P15531
Inner	P15924
Inner	P16422
Inner	P18754
Inner	P19338
Inner	P20290
Inner	P20810
Inner	P20936
Inner	P21912
Inner	P22061
Inner	P23434
Inner	P23528
Inner	P27544
Inner	P27797
Inner	P27824
Inner	P28066
Inner	P30085
Inner	P30456
Inner	P30837
Inner	P31942
Inner	P31943
Inner	P31947
Inner	P31948
Inner	P32242
Inner	P32322

Layer	Master Protein Accession
Inner	P34931
Inner	P34932
Inner	P35609
Inner	P36551
Inner	P40926
Inner	P41567
Inner	P43652
Inner	P46777
Inner	P46782
Inner	P48643
Inner	P49411
Inner	P49792
Inner	P50402
Inner	P51858
Inner	P54709
Inner	P55010
Inner	P55209
Inner	P55795
Inner	P60174
Inner	P60660
Inner	P61758
Inner	P61978
Inner	P62306
Inner	P62854
Inner	P63104
Inner	P63208
Inner	P67809
Inner	P68104
Inner	P78563

Layer	Master Protein Accession
Inner	P84090
Inner	Q01844
Inner	Q02790
Inner	Q02818
Inner	Q03252
Inner	Q04837
Inner	Q07955
Inner	Q09666
Inner	Q13099
Inner	Q13428
Inner	Q13451
Inner	Q14126
Inner	Q15417
Inner	Q15459
Inner	Q15637
Inner	Q16629
Inner	Q3ZCM7
Inner	Q4VC31
Inner	Q562R1
Inner	Q58FF7
Inner	Q5JU67
Inner	Q5K4E3
Inner	Q5T4F4
Inner	Q5TCZ1
Inner	Q5TG30
Inner	Q5TH69
Inner	Q5VUJ5
Inner	Q6IBS0
Inner	Q6UW63

Layer	Master Protein Accession
Inner	Q6UY14
Inner	Q76I76
Inner	Q7Z3B4
Inner	Q7Z3H0
Inner	Q7Z7H5
Inner	Q8IZP2
Inner	Q8N4N3
Inner	Q8NFA0
Inner	Q8NFP9
Inner	Q8TB52
Inner	Q8TCC7
Inner	Q8TF45
Inner	Q8WTR4
Inner	Q8WTT2
Inner	Q8WUH2
Inner	Q8WX94
Inner	Q8WXD9
Inner	Q8WZ42
Inner	Q92945
Inner	Q96LW1
Inner	Q96NW7
Inner	Q96T17
Inner	Q9BVK6
Inner	Q9BWQ6
Inner	Q9BX26
Inner	Q9H299
Inner	Q9H307
Inner	Q9H3H3
Inner	Q9H5V8

Layer	Master Protein Accession
Inner	Q9H9P8
Inner	Q9HAV7
Inner	Q9NQT5
Inner	Q9NS69
Inner	Q9NUD5
Inner	Q9NWU2
Inner	Q9NX55
Inner	Q9NZJ4
Inner	Q9P0U1
Inner	Q9P2E9
Inner	Q9UBS4
Inner	Q9UHD9
Inner	Q9UHV9
Inner	Q9UIG8
Inner	Q9UJZ1
Inner	Q9UKK3
Inner	Q9UKY7
Inner	Q9UMX0
Inner	Q9Y281
Inner	Q9Y3C1
Core	A0A0B4J1Y8
Core	A0JNW5
Core	A6NC97
Core	A6ND36
Core	A6NDG6
Core	A6NGB9
Core	O00151
Core	O00193
Core	O00217

Layer	Master Protein Accession
Core	O00264
Core	O00515
Core	O14561
Core	O14737
Core	O14757
Core	O14950
Core	O14974
Core	O15061
Core	O15347
Core	O15355
Core	O15511
Core	O43314
Core	O43399
Core	O43707
Core	O43768
Core	O43852
Core	O60281
Core	O60290
Core	O60506
Core	O60664
Core	O60749
Core	O60814
Core	O75190
Core	O75369
Core	O75380
Core	O75400
Core	O75461
Core	O75475
Core	O75487

Layer	Master Protein Accession
Core	O75531
Core	O75582
Core	O75781
Core	O75822
Core	O75874
Core	O75947
Core	O76050
Core	O94826
Core	O94986
Core	O94992
Core	O95169
Core	O95292
Core	O95396
Core	P00338
Core	P00352
Core	P00441
Core	P00558
Core	P01892
Core	P02042
Core	P02545
Core	P04350
Core	P04406
Core	P04632
Core	P04792
Core	P05067
Core	P05386
Core	P05387
Core	P05534
Core	P05556

Layer	Master Protein Accession
Core	P05783
Core	P05787
Core	P06576
Core	P06733
Core	P06748
Core	P07108
Core	P07237
Core	P07305
Core	P07437
Core	P07602
Core	P07900
Core	P07942
Core	P07951
Core	P08238
Core	P08708
Core	P08727
Core	P08758
Core	P08779
Core	P08962
Core	P09211
Core	P09382
Core	P09429
Core	P09455
Core	P0DMV9
Core	P0DP25
Core	P10316
Core	P10319
Core	P10599
Core	P10636

Layer	Master Protein Accession
Core	P10809
Core	P11021
Core	P11142
Core	P11308
Core	P12270
Core	P12277
Core	P12429
Core	P13073
Core	P13639
Core	P13646
Core	P13667
Core	P13693
Core	P14314
Core	P14618
Core	P14625
Core	P15531
Core	P16070
Core	P16118
Core	P16189
Core	P16422
Core	P16473
Core	P17677
Core	P17931
Core	P17980
Core	P18206
Core	P18669
Core	P18859
Core	P19338
Core	P20290

Layer	Master Protein Accession
Core	P20674
Core	P20700
Core	P20810
Core	P21912
Core	P22061
Core	P22105
Core	P22307
Core	P22392
Core	P22626
Core	P23434
Core	P23497
Core	P23511
Core	P24534
Core	P24844
Core	P25092
Core	P25705
Core	P26447
Core	P26583
Core	P27348
Core	P27708
Core	P27797
Core	P27816
Core	P27824
Core	P28066
Core	P29692
Core	P29966
Core	P30040
Core	P30101
Core	P30405

Layer	Master Protein Accession
Core	P30443
Core	P30519
Core	P30533
Core	P30626
Core	P30837
Core	P31946
Core	P31947
Core	P31948
Core	P32322
Core	P34932
Core	P35221
Core	P35579
Core	P35637
Core	P35998
Core	P37198
Core	P37802
Core	P38646
Core	P41208
Core	P41567
Core	P43007
Core	P43243
Core	P43307
Core	P43652
Core	P46060
Core	P46937
Core	P48960
Core	P49006
Core	P49321
Core	P49454

Layer	Master Protein Accession
Core	P50213
Core	P50402
Core	P50502
Core	P50552
Core	P50990
Core	P51572
Core	P51858
Core	P51991
Core	P52630
Core	P53539
Core	P53597
Core	P53999
Core	P54709
Core	P54727
Core	P54819
Core	P55010
Core	P55209
Core	P60174
Core	P60660
Core	P60709
Core	P61088
Core	P61129
Core	P61204
Core	P61604
Core	P61970
Core	P61978
Core	P61981
Core	P62191
Core	P62258

Layer	Master Protein Accession
Core	P62306
Core	P62805
Core	P62841
Core	P62937
Core	P62942
Core	P63104
Core	P63220
Core	P67809
Core	P67936
Core	P68032
Core	P68104
Core	P68133
Core	P68371
Core	P69905
Core	P80303
Core	P82909
Core	P82979
Core	P83916
Core	Q00059
Core	Q00610
Core	Q00688
Core	Q00839
Core	Q01130
Core	Q01628
Core	Q01844
Core	Q02818
Core	Q02952
Core	Q03252
Core	Q04760

Layer	Master Protein Accession
Core	Q04917
Core	Q06323
Core	Q06830
Core	Q07021
Core	Q07866
Core	Q08462
Core	Q08AF3
Core	Q09666
Core	Q12874
Core	Q13029
Core	Q13123
Core	Q13185
Core	Q13263
Core	Q13315
Core	Q13404
Core	Q13428
Core	Q13435
Core	Q13509
Core	Q13541
Core	Q13561
Core	Q13573
Core	Q13813
Core	Q13838
Core	Q13873
Core	Q13885
Core	Q14050
Core	Q14103
Core	Q14126
Core	Q14157

Layer	Master Protein Accession
Core	Q14247
Core	Q14444
Core	Q14697
Core	Q15056
Core	Q15185
Core	Q15293
Core	Q15365
Core	Q15366
Core	Q15417
Core	Q15424
Core	Q15459
Core	Q15637
Core	Q15642
Core	Q15819
Core	Q15836
Core	Q16543
Core	Q16595
Core	Q16629
Core	Q16658
Core	Q16890
Core	Q16891
Core	Q29960
Core	Q2VIQ3
Core	Q32MZ4
Core	Q4VC31
Core	Q53H47
Core	Q562R1
Core	Q58FF7
Core	Q5JRA6

Layer	Master Protein Accession
Core	Q5JU67
Core	Q5JUR7
Core	Q5S007
Core	Q5TG30
Core	Q5TH69
Core	Q5VZF2
Core	Q68DQ2
Core	Q6N021
Core	Q6TFL3
Core	Q6WKZ4
Core	Q6XUX3
Core	Q7Z434
Core	Q7Z4H3
Core	Q7Z7H5
Core	Q86UD1
Core	Q86UE4
Core	Q86V81
Core	Q86V85
Core	Q86Y82
Core	Q8IWA4
Core	Q8IWW8
Core	Q8IYI8
Core	Q8N0Y7
Core	Q8NBS9
Core	Q8NC51
Core	Q8NDA2
Core	Q8NFA0
Core	Q8NFI4
Core	Q8NFP9

Layer	Master Protein Accession
Core	Q8NFU3
Core	Q8NHQ9
Core	Q8TEX9
Core	Q8TF27
Core	Q8WU90
Core	Q8WWI1
Core	Q8WXB4
Core	Q8WXD9
Core	Q8WXI9
Core	Q8WXX5
Core	Q92520
Core	Q92597
Core	Q92766
Core	Q92804
Core	Q92945
Core	Q96AE4
Core	Q96PE2
Core	Q99439
Core	Q99497
Core	Q99729
Core	Q99961
Core	Q99996
Core	Q9BRB3
Core	Q9BRK5
Core	Q9BTT0
Core	Q9BUF5
Core	Q9BUL8
Core	Q9BVK6
Core	Q9BXY4

Layer	Master Protein Accession
Core	Q9BY11
Core	Q9BYJ4
Core	Q9C0C2
Core	Q9H169
Core	Q9H299
Core	Q9H2Z4
Core	Q9H307
Core	Q9H4B7
Core	Q9H910
Core	Q9HAV7
Core	Q9HAZ1
Core	Q9HC07
Core	Q9NQC3
Core	Q9NQC7
Core	Q9NR48
Core	Q9NS69
Core	Q9NTJ3
Core	Q9NUD5
Core	Q9NUV7
Core	Q9NX40
Core	Q9NX58
Core	Q9NYF8
Core	Q9NYL9
Core	Q9NZC3
Core	Q9P013
Core	Q9P032
Core	Q9P0J7
Core	Q9P0L0
Core	Q9P0U1

Layer	Master Protein Accession
Core	Q9P275
Core	Q9P2E9
Core	Q9UBU3
Core	Q9UGP4
Core	Q9UHV7
Core	Q9UHV9
Core	Q9UII2
Core	Q9UJZ1
Core	Q9UKK3
Core	Q9UKY7
Core	Q9ULJ1
Core	Q9ULV0
Core	Q9UMS4
Core	Q9UMX0
Core	Q9UPM8
Core	Q9UPS6
Core	Q9Y224
Core	Q9Y253
Core	Q9Y266
Core	Q9Y2B0
Core	Q9Y2W1
Core	Q9Y3C1
Core	Q9Y3D0
Core	Q9Y3U8
Core	Q9Y4B5
Core	Q9Y4C4
Core	Q9Y4J8
Core	Q9Y4W6
Core	Q9Y520

Layer	Master Protein Accession
Core	Q9Y566
Core	Q9Y597
Core	Q9Y5S9
Core	Q9Y608
Core	Q9Y639
Core	Q9Y6I3
Core	Q9Y6K8
Core	Q9Y6M1
Core	Q9YNA8

Table S4: Total unique proteins modified by FPOP using 200 mM H_2O_2

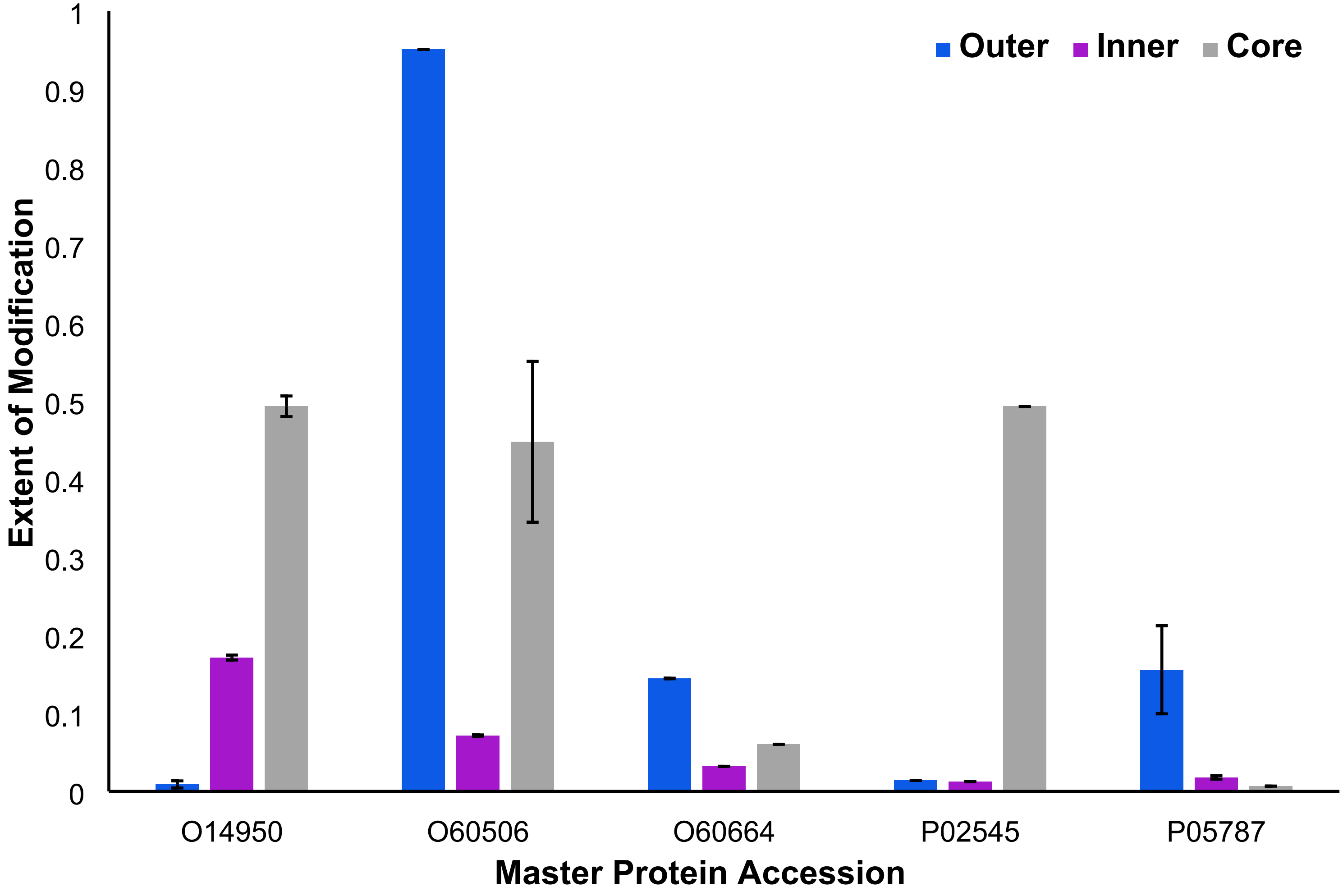

Figure S5: Global Extent of Modification for 5 Proteins Modified in All Spheroid Layers.

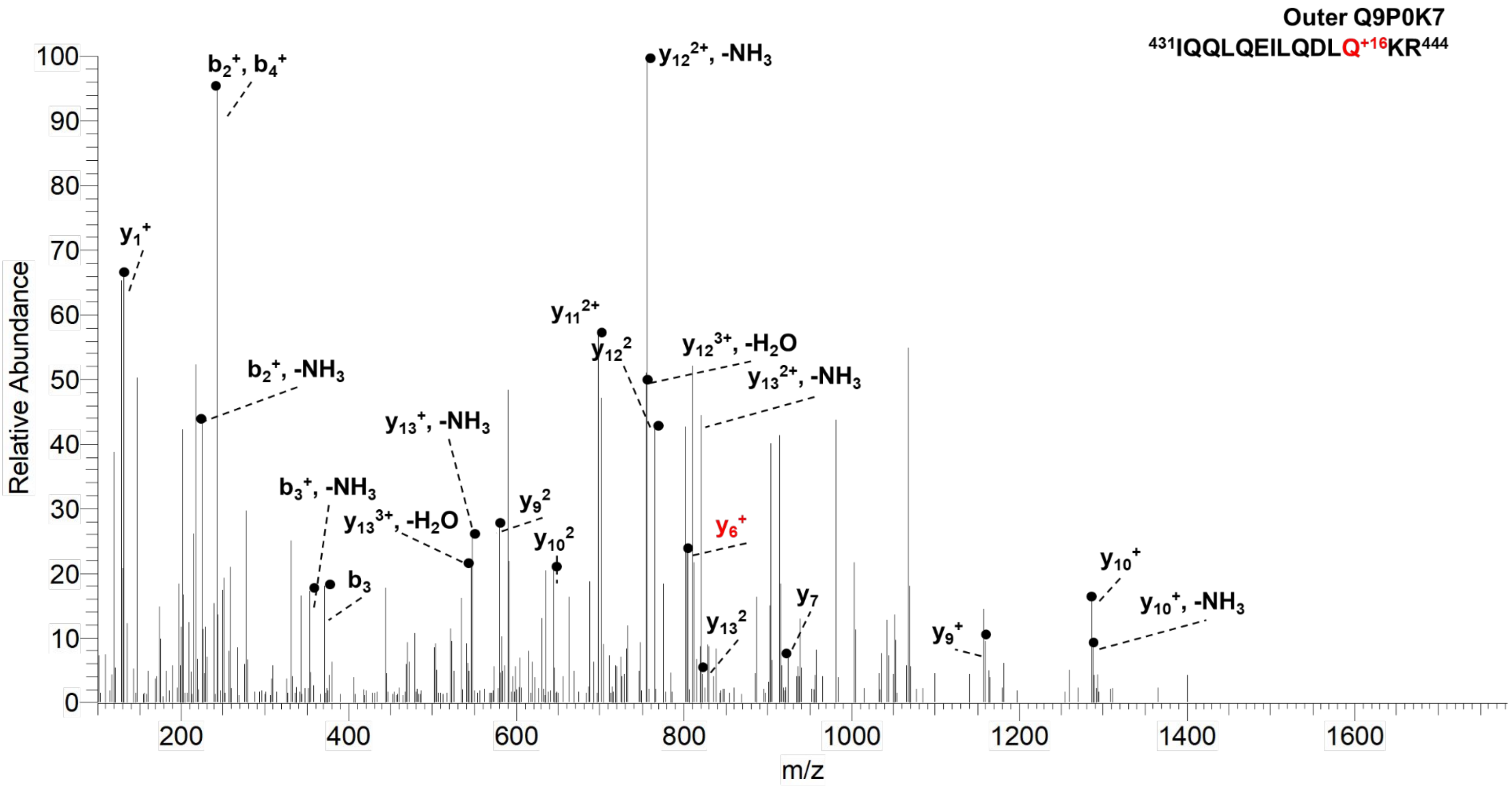

Figure S6: Tandem Mass Spectra of a Modified Peptide on Protein Q9P0K7 from the Outer Layer. B- and y- ions are visualized along with a +16 FPOP Modification on Glutamine.

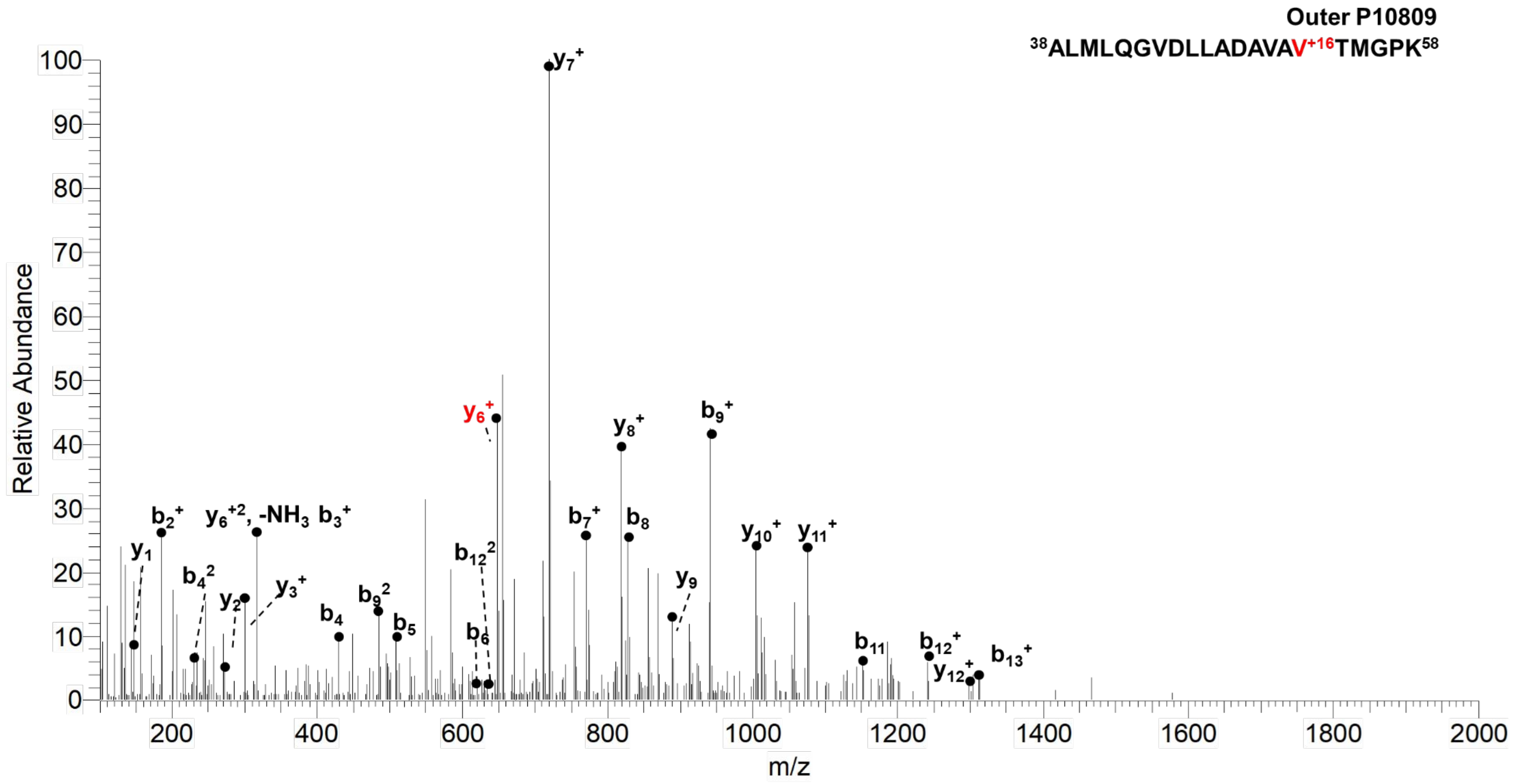

Figure S7: Tandem Mass Spectra of a Modified Peptide on Protein P10809 from the Outer Layer. B- and y- ions are visualized along with a +16 FPOP Modification on Valine.

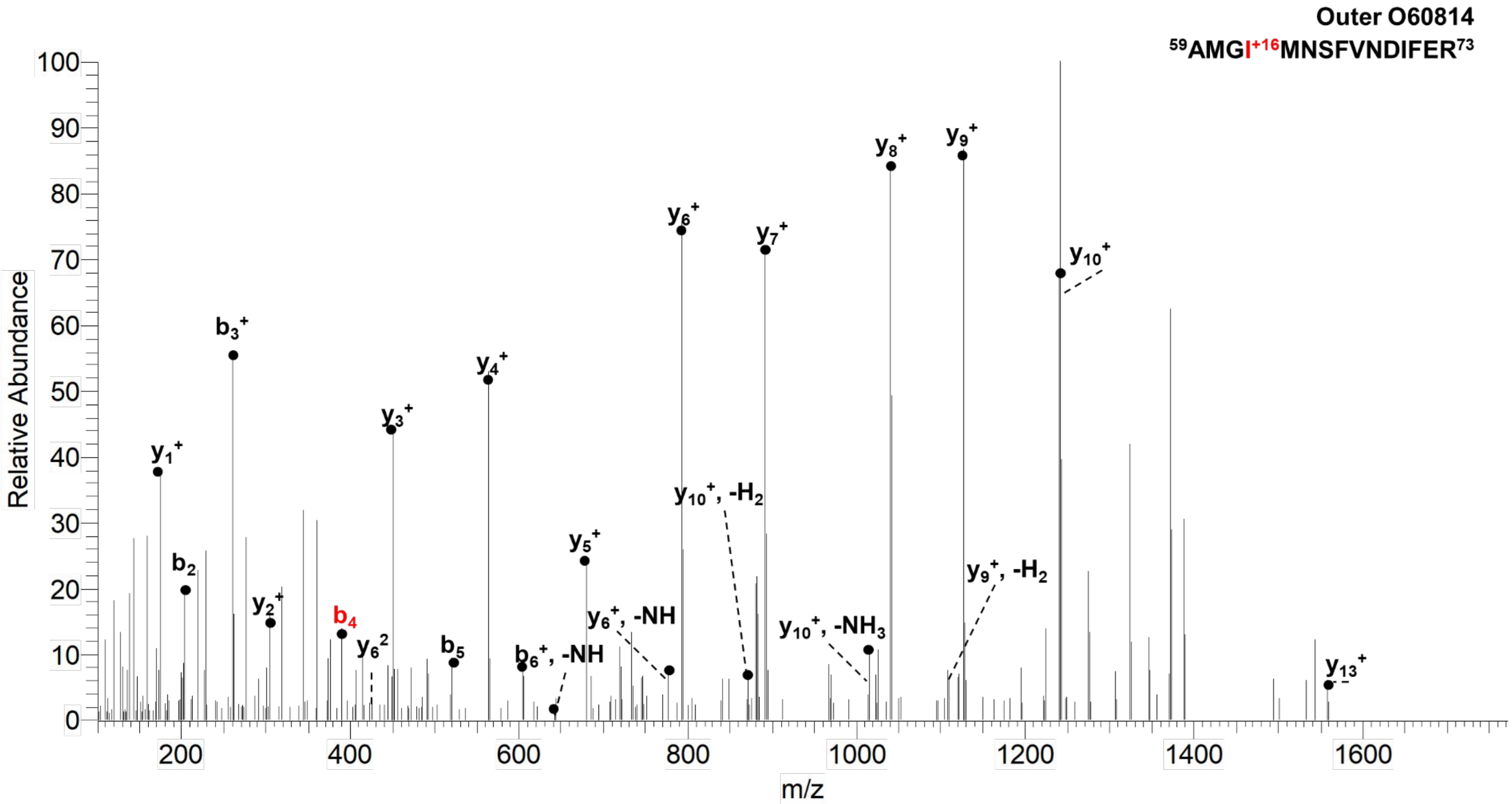

Figure S8: Tandem Mass Spectra of a Modified Peptide on Protein O60814 from the Outer Layer. B- and y- ions are visualized along with a +16 FPOP Modification on Isoleucine.

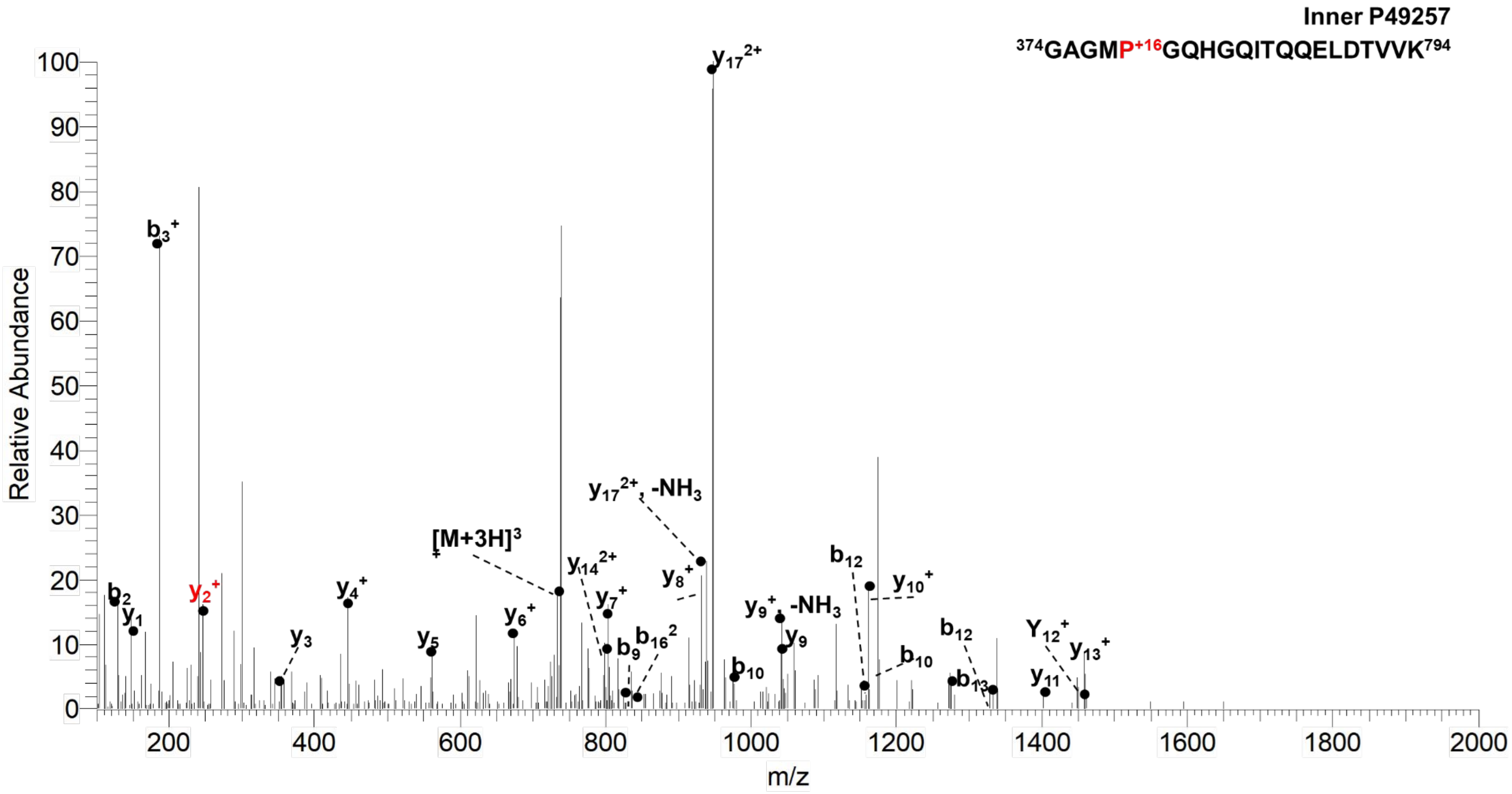

Figure S9: Tandem Mass Spectra of a Modified Peptide on Protein P49257 from the Inner Layer. B- and y- ions are visualized along with a +16 FPOP Modification on Proline.

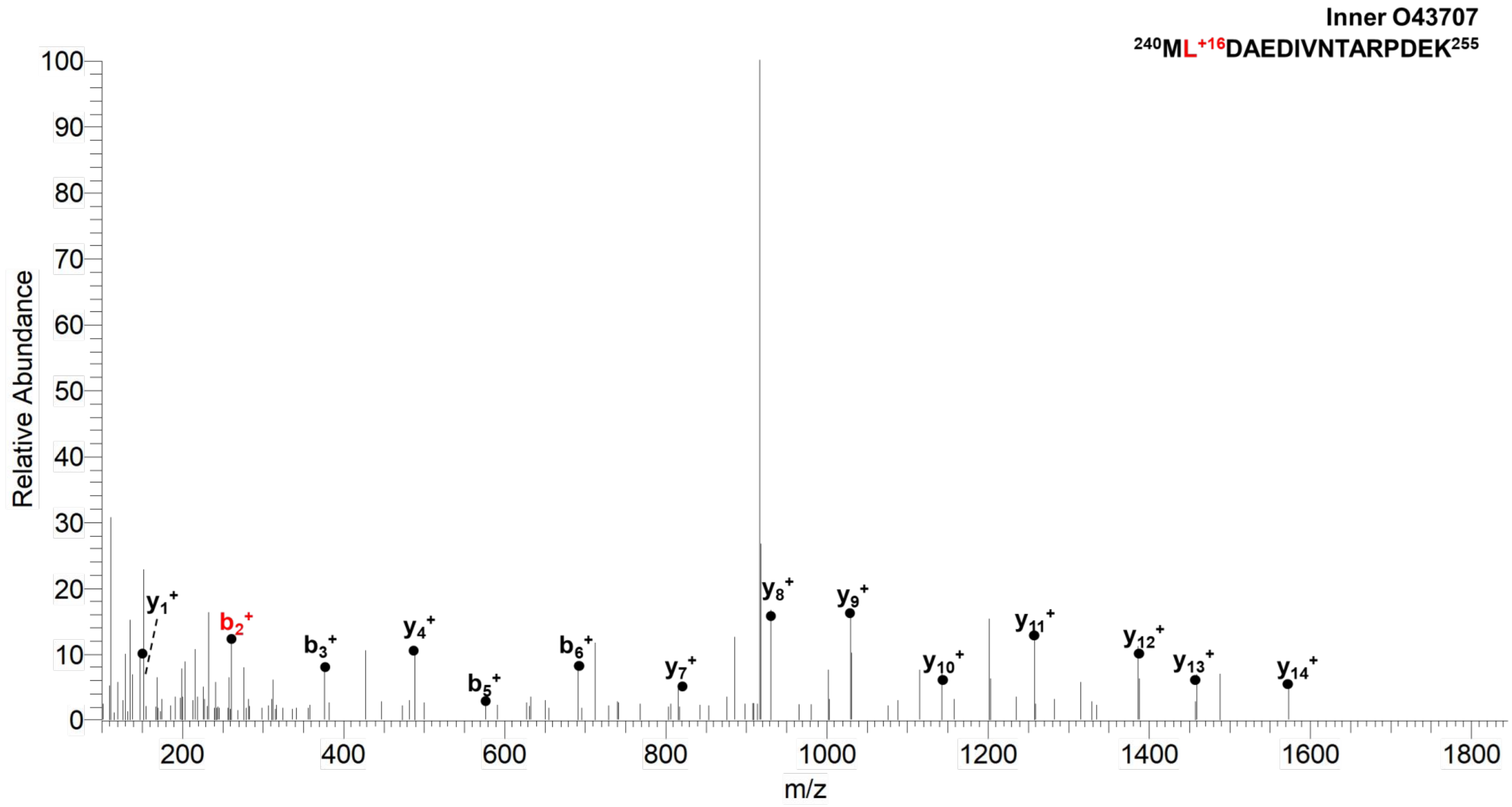

Figure S10: Tandem Mass Spectra of a Modified Peptide on Protein O43707 from the Inner Layer. B- and y- ions are visualized along with a +16 FPOP Modification on Leucine.

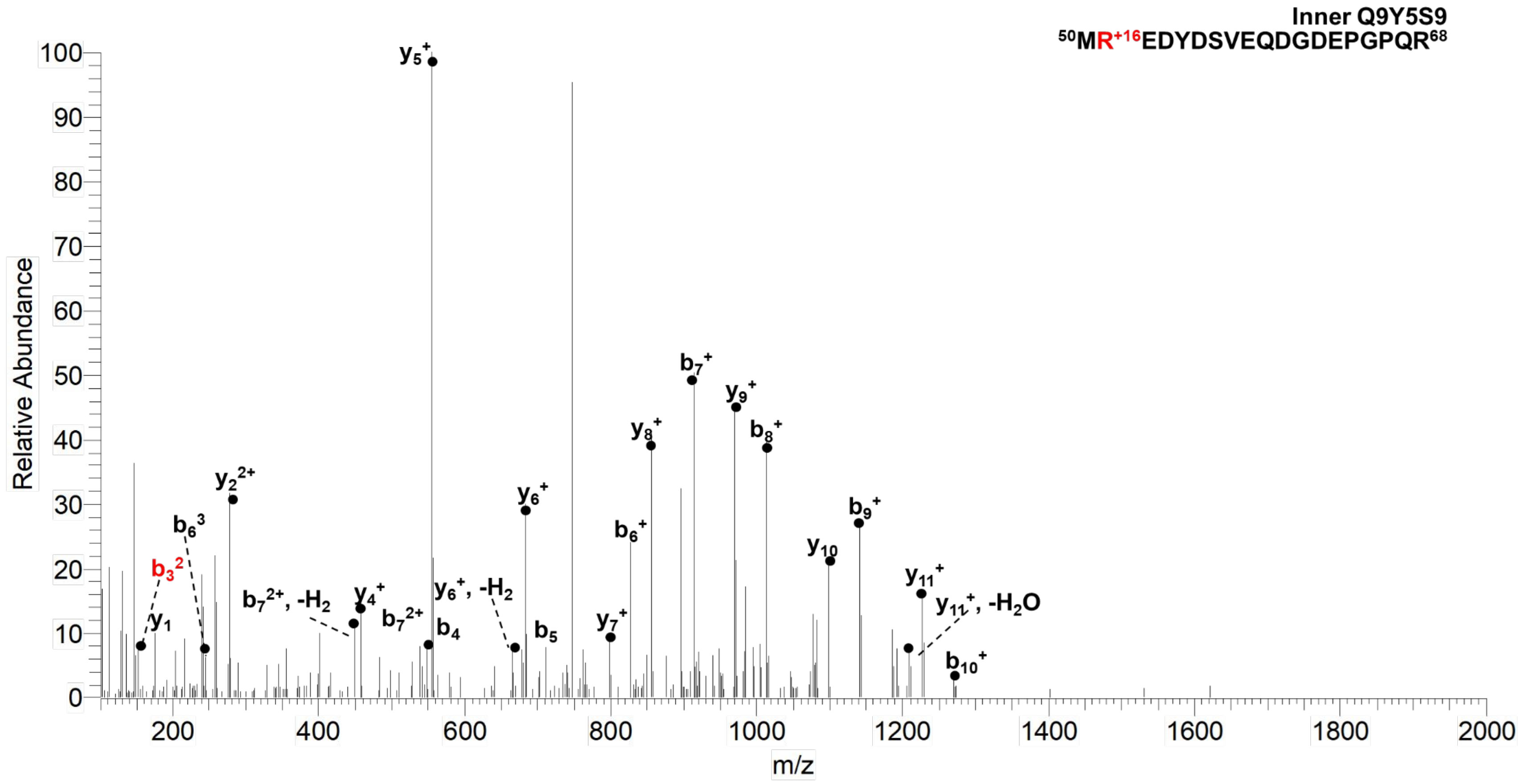

Figure S11: Tandem Mass Spectra of a Modified Peptide on Protein Q9Y5S9 from the Inner Layer. B- and y- ions are visualized along with a +16 FPOP Modification on Arginine.

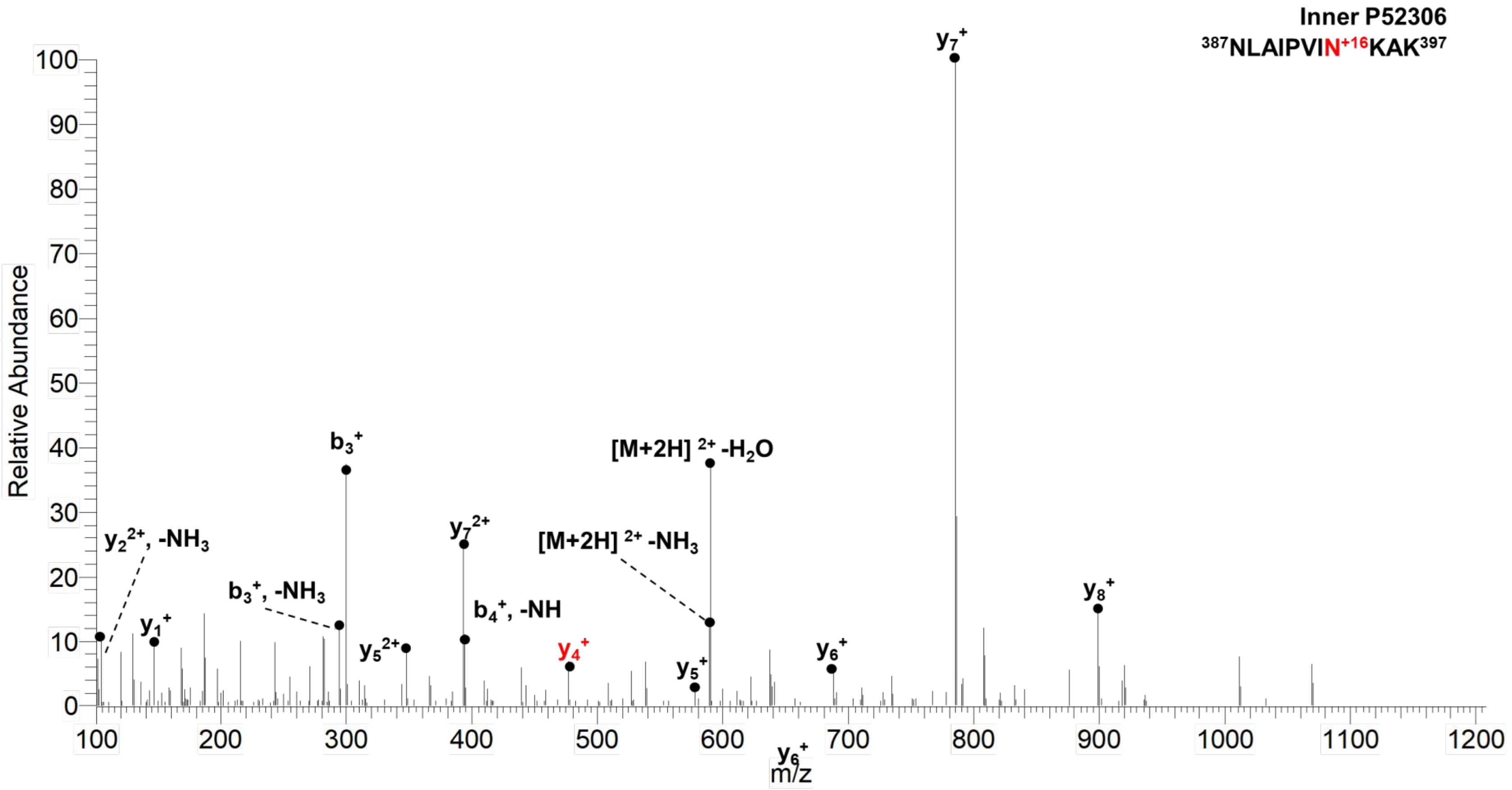

Figure S12: Tandem Mass Spectra of a Modified Peptide on Protein P52306 from the Inner Layer. B- and y- ions are visualized along with a +16 FPOP Modification on Asparagine.

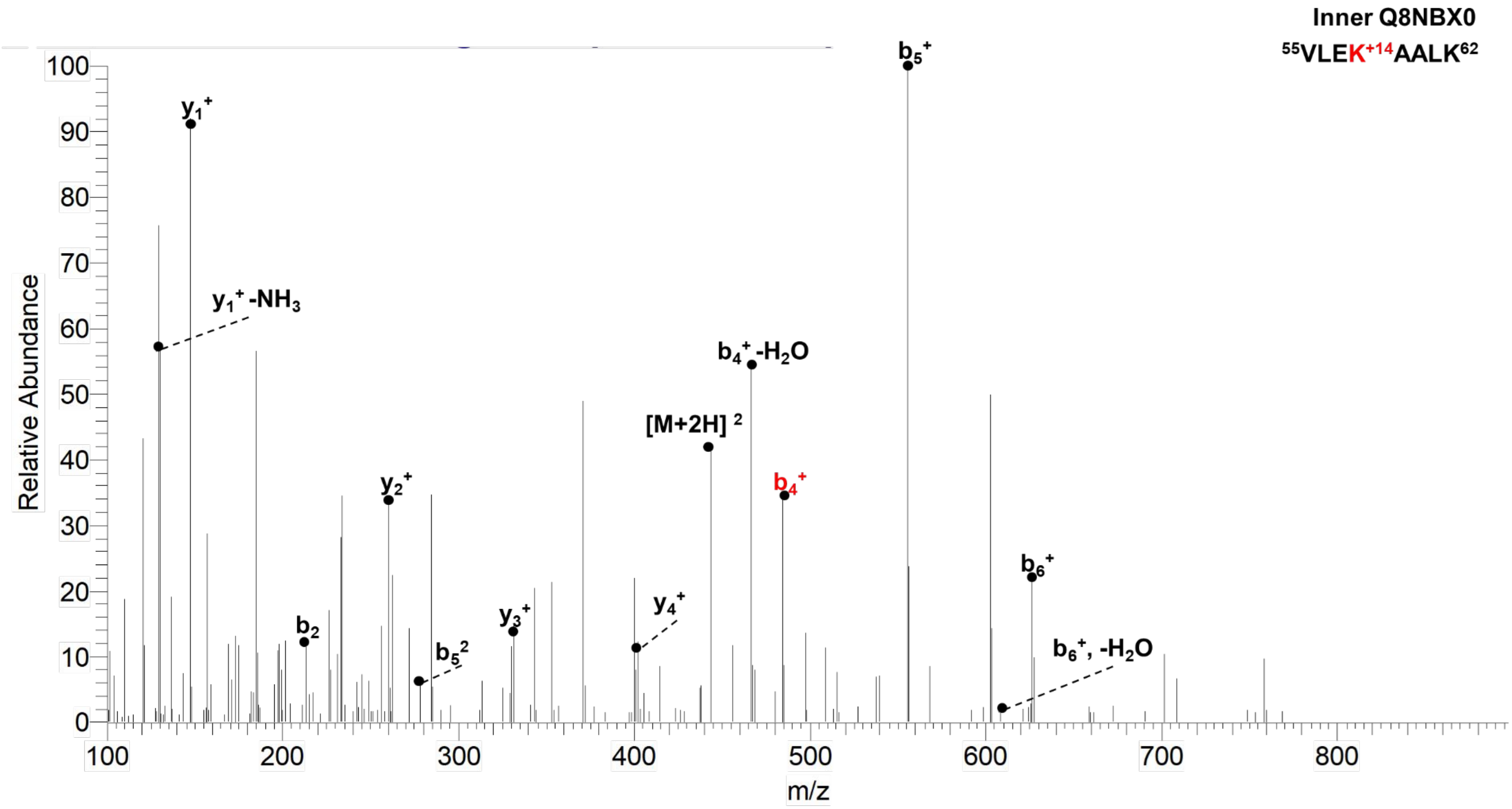

Figure S13: Tandem Mass Spectra of a Modified Peptide on Protein Q8NBX0 from the Inner Layer. B- and y- ions are visualized along with a +14 FPOP Modification on Lysine.

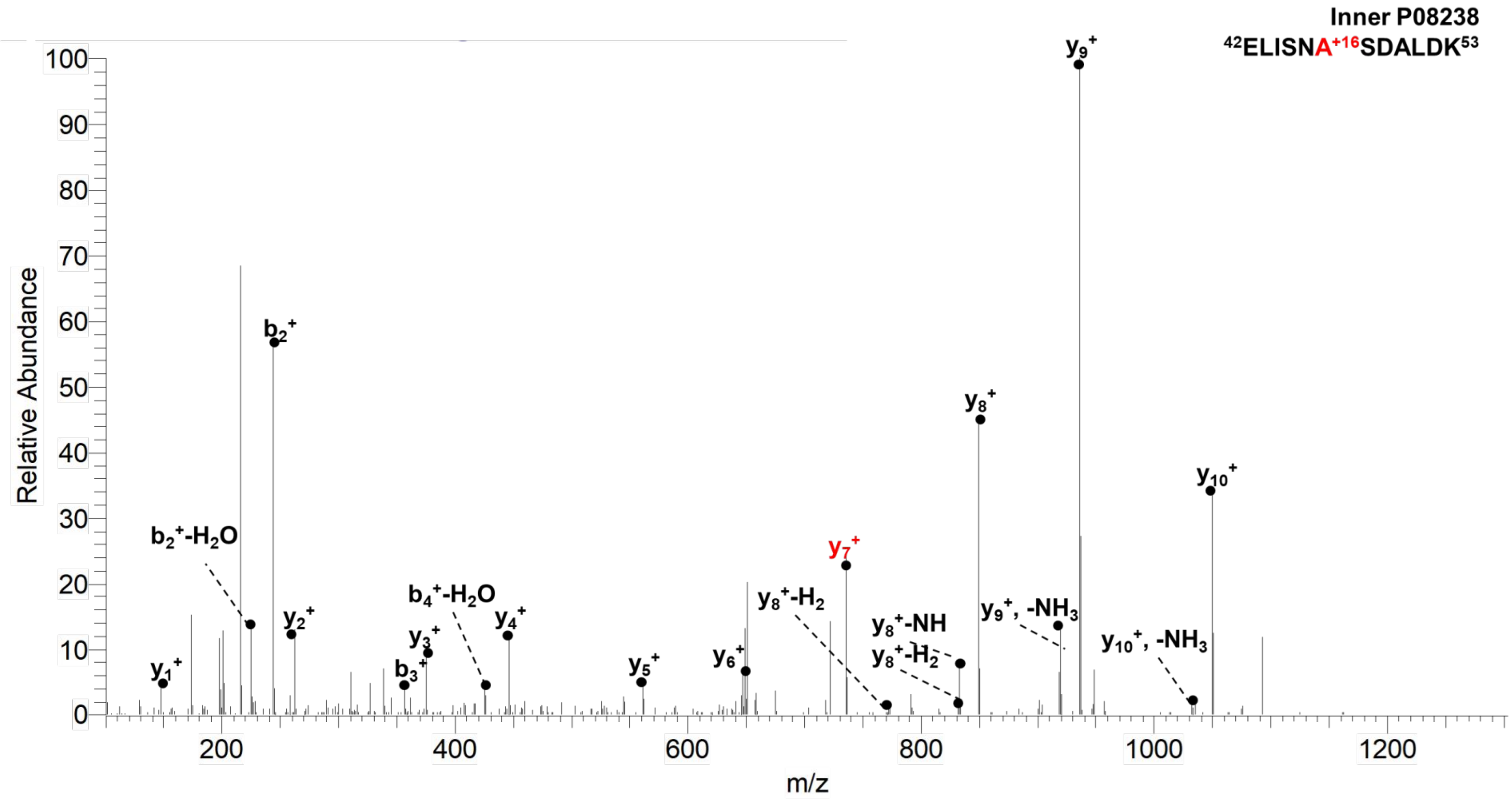

Figure S14: Tandem Mass Spectra of a Modified Peptide on Protein P08238 from the Inner Layer. B- and y- ions are visualized along with a +16 FPOP Modification on Alanine.

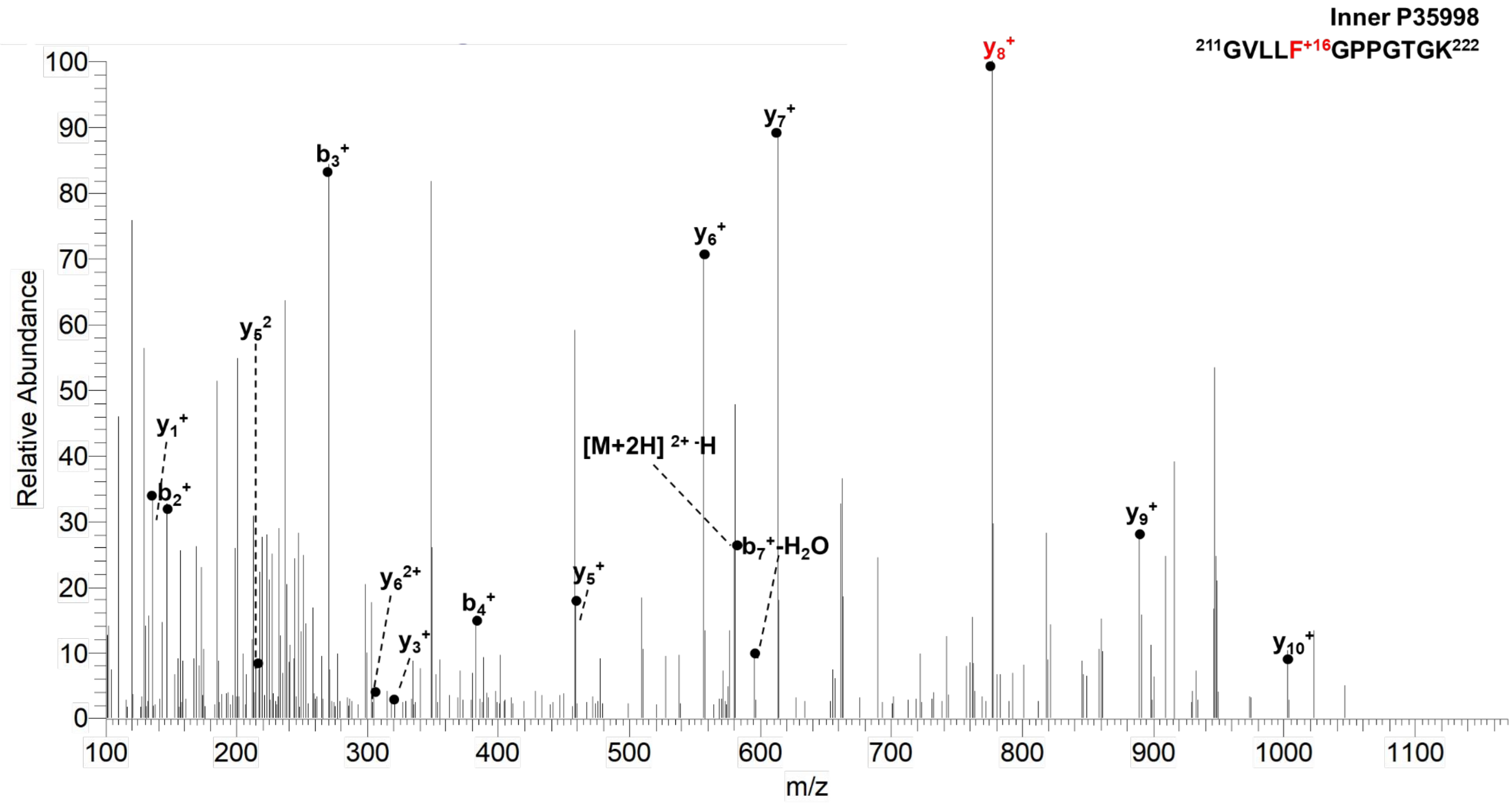

Figure S15: Tandem Mass Spectra of a Modified Peptide on Protein P35998 from the Inner Layer. B- and y- ions are visualized along with a +16 FPOP Modification on Phenylalanine.

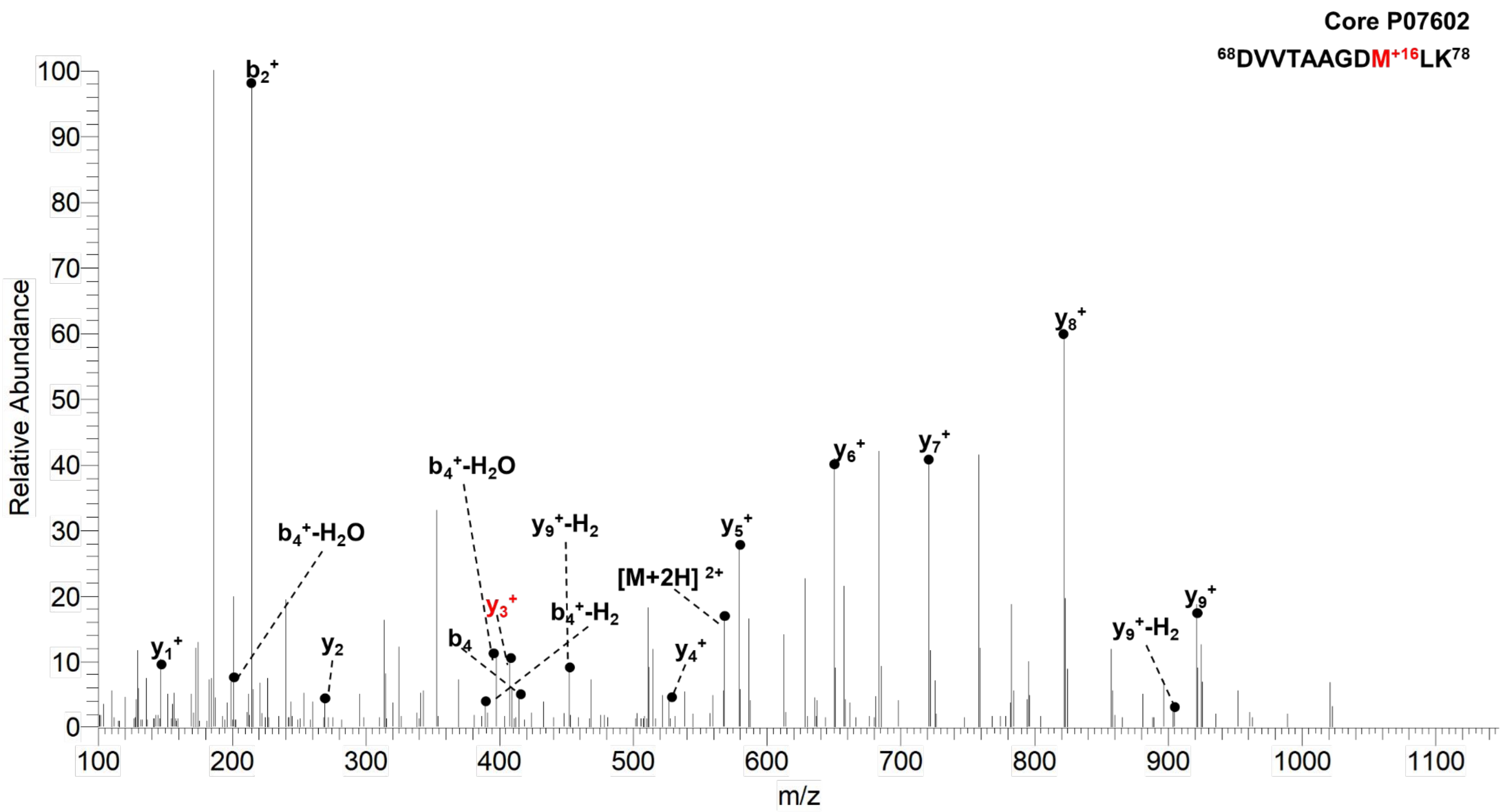

Figure S16: Tandem Mass Spectra of a Modified Peptide on Protein P07602 from the Core Layer. B- and y- ions are visualized along with a +16 FPOP Modification on Methionine.

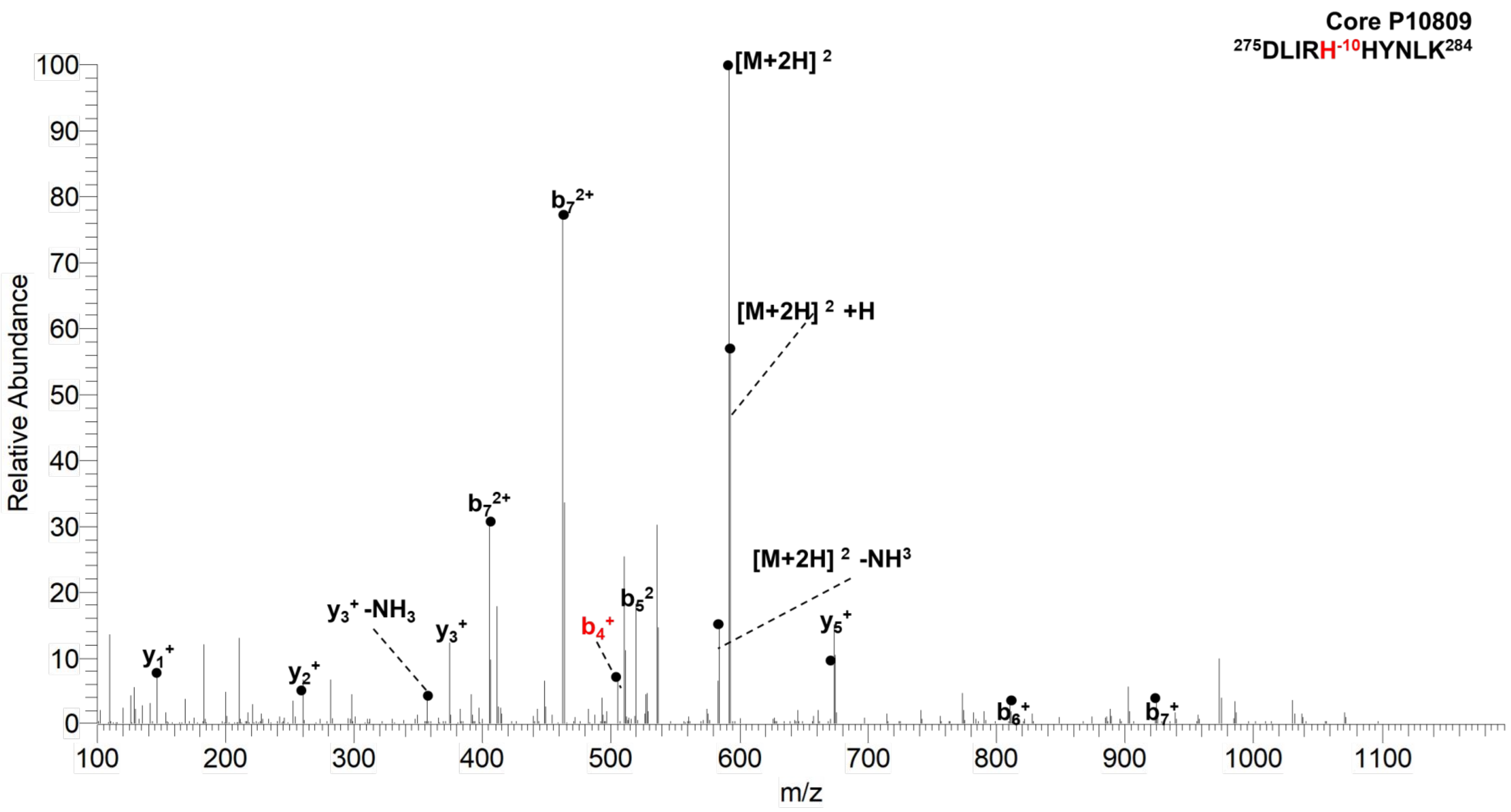

Figure S17: Tandem Mass Spectra of a Modified Peptide on Protein P10809 from the Core Layer. B- and y- ions are visualized along with a -10 FPOP Modification on Histidine.

.

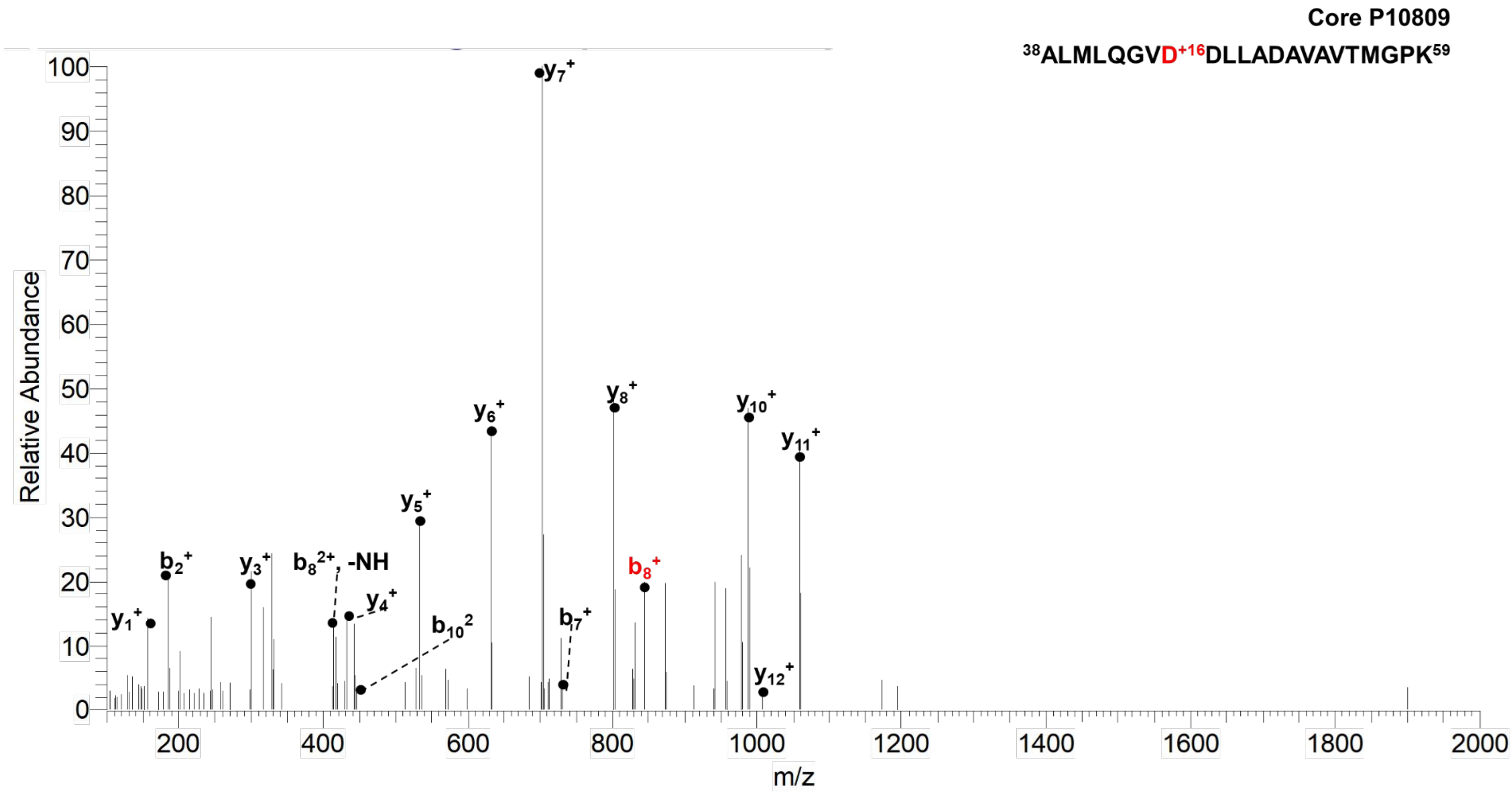

Figure S18: Tandem Mass Spectra of a Modified Peptide on Protein P10809 from the Core Layer. B- and y- ions are visualized along with a +16 FPOP Modification on Aspartic Acid

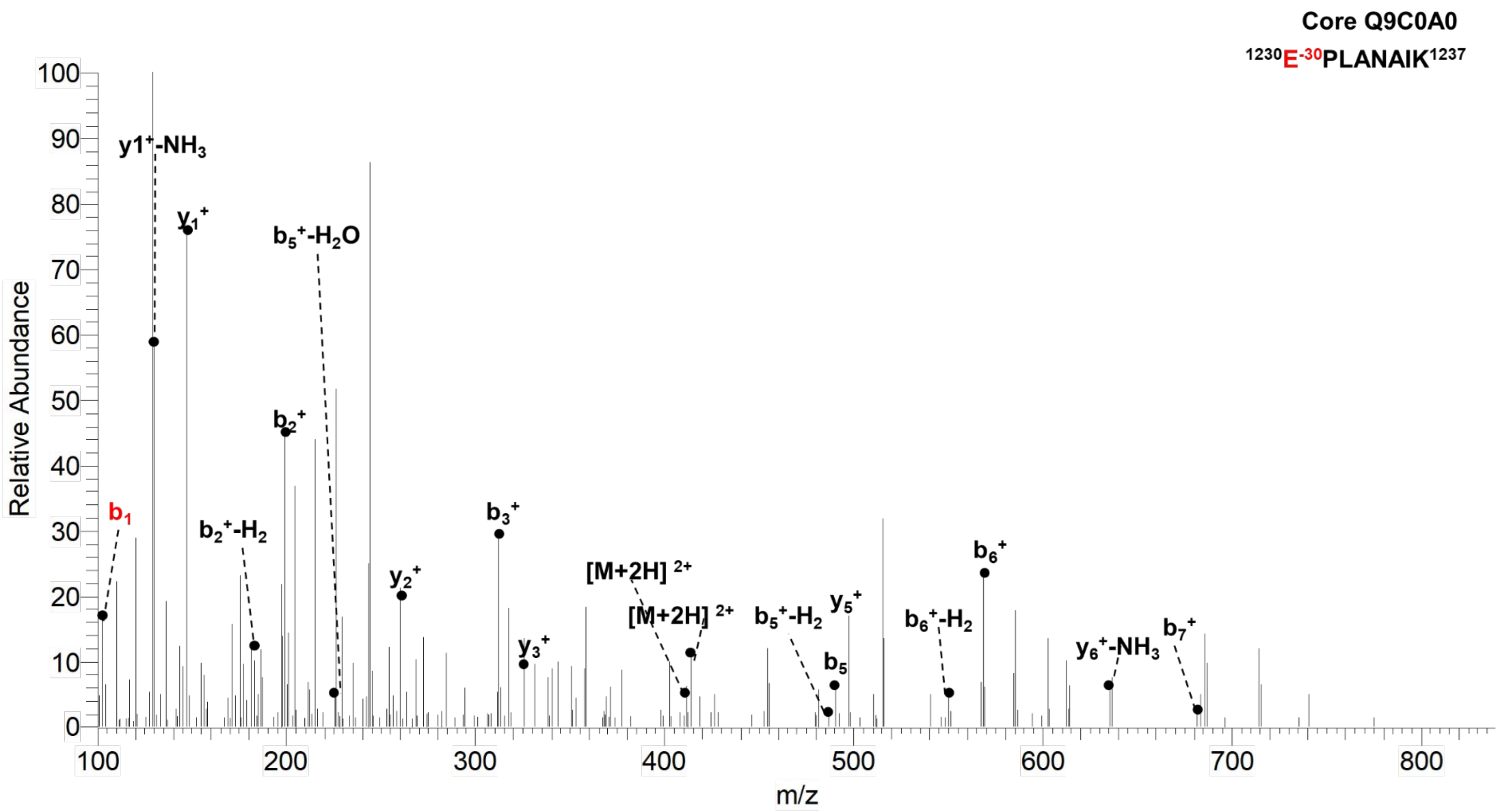

Figure S19: Tandem Mass Spectra of a Modified Peptide on Protein P10809 from the Core Layer. B- and y- ions are visualized along with a -30 FPOP Modification on Glutamic Acid.
